# DNA vaccine priming for seasonal influenza vaccine in children and adolescents 6 to 17 years of age: A phase 1 randomized clinical trial

**DOI:** 10.1371/journal.pone.0206837

**Published:** 2018-11-02

**Authors:** Katherine V. Houser, Galina V. Yamshchikov, Abbie R. Bellamy, Jeanine May, Mary E. Enama, Uzma Sarwar, Brenda Larkin, Robert T. Bailer, Richard Koup, Myeisha Paskel, Kanta Subbarao, Edwin Anderson, David I. Bernstein, Buddy Creech, Harry Keyserling, Paul Spearman, Peter F. Wright, Barney S. Graham, Julie E. Ledgerwood, the VRC 702 study team

**Affiliations:** 1 Vaccine Research Center, National Institute of Allergy and Infectious Diseases, National Institutes of Health, Bethesda, MD, United States of America; 2 The Emmes Corporation, Rockville, MD, United States of America; 3 Laboratory of Infectious Diseases, National Institute of Allergy and Infectious Diseases, National Institutes of Health, Bethesda, MD, United States of America; 4 Department of Internal Medicine, Saint Louis University, Saint Louis, MO, United States of America; 5 Cincinnati Children’s Hospital Medical Center, University of Cincinnati College of Medicine, Cincinnati, OH, United States of America; 6 Vanderbilt Vaccine Research Program, Department of Pediatrics, Vanderbilt University School of Medicine, Nashville, TN, United States of America; 7 Department of Pediatrics, Emory University School of Medicine, Atlanta, GA, United States of America; 8 Department of Pediatrics, Geisel School of Medicine, Dartmouth College, Lebanon, NH, United States of America; National Institute of Animal Biotechnology, INDIA

## Abstract

**Background:**

Children are susceptible to severe influenza infections and facilitate community transmission. One potential strategy to improve vaccine immunogenicity in children against seasonal influenza involves a trivalent hemagglutinin DNA prime-trivalent inactivated influenza vaccine (IIV3) boost regimen.

**Methods:**

Sites enrolled adolescents, followed by younger children, to receive DNA prime (1 mg or 4 mg) intramuscularly by needle-free jet injector (Biojector), followed by split virus 2012/13 seasonal IIV3 boost by needle and syringe approximately 18 weeks later. A comparator group received IIV3 prime and boost at similar intervals. Primary study objectives included evaluation of the safety and tolerability of the vaccine regimens, with secondary objectives of measuring antibody responses at four weeks post boost by hemagglutination inhibition (HAI) and neutralization assays.

**Results:**

Seventy-five children ≥6 to ≤17 years old enrolled. Local reactogenicity was higher after DNA prime compared to IIV3 prime (p<0.001 for pain/tenderness, redness, or swelling), but symptoms were mild to moderate in severity. Systemic reactogenicity was similar between vaccines. Overall, antibody responses were similar among groups, although HAI antibodies revealed a trend towards higher responses following 4 mg DNA-IIV3 compared to IIV3-IIV3. The fold increase of HAI antibodies to A/California/07/2009 [A(H1N1)pdm09] was significantly greater following 4 mg DNA-IIV3 (10.12 fold, 5.60–18.27 95%CI) compared to IIV3-IIV3 (3.86 fold, 2.32–6.44 95%CI). Similar neutralizing titers were observed between regimens, with a trend towards increased response frequencies in 4 mg DNA-IIV3. However, significant differences in fold increase, reported as geometric mean fold ratios, were detected against the H1N1 viruses within the neutralization panel: A/New Caledonia/20/1999 (1.41 fold, 1.10–1.81 95%CI) and A/South Carolina/1/1918 (1.55 fold, 1.27–1.89 95%CI).

**Conclusions:**

In this first pediatric DNA vaccine study conducted in the U.S., the DNA prime-IIV3 boost regimen was safe and well tolerated. In children, the 4 mg DNA-IIV3 regimen resulted in antibody responses comparable to the IIV3-IIV3 regimen.

## Introduction

Each influenza season children have an increased burden of influenza infection [1] and facilitate disease transmission to others in their communities [2]. Children between 2 and 17 years of age have the highest rates of influenza-positive influenza-like illness (ILI) in outpatient clinics [3], and school-age children are typically the main source of transmission in household settings [4, 5]. Severe disease requiring hospitalization is also substantially higher in children under 5 years of age [6]. In addition, outpatient clinic visits and days missed from school or work (for children and parents) can result in a significant economic and public health impact [7].

Vaccination remains the most effective way of preventing both influenza infection and disease in children and adults, although vaccine efficacy needs to be improved [8]. The vaccine predominantly administered to children each year is an inactivated influenza vaccine (IIV) preparation that is updated annually and has an overall vaccine efficacy of 59–64% [9]. This efficacy can be lower when the selected vaccine strains are antigenically distinct from those currently circulating in the community [8]. Even during years where the vaccine strains closely match those circulating, IIVs only reduce outpatient medical visits caused by circulating influenza viruses by 50 to 75% [10]. Additional disadvantages to the current vaccine strategies exist, including a long production time and a dependence on embryonated eggs [11].

Various strategies, including the use of adjuvants or DNA vaccines, have been suggested as ways of improving vaccine immunogenicity in children while avoiding the aforementioned limitations of the licensed vaccines [11–13]. DNA vaccines are a particularly appealing strategy since this platform has been shown to be safe and immunogenic in healthy adults against multiple viruses [14–21] without requiring eggs for production or preservatives in the final vaccine preparation [22]. Also, since DNA vaccines require shorter time for development and production compared to inactivated vaccines, vaccination of vulnerable populations with DNA vaccines may start earlier in a pandemic situation while inactivated vaccines are still being manufactured [22].

In healthy adults, studies with DNA influenza vaccines against emerging subtypes of avian origin (including H5 and H7) administered as a prime injection prior to an inactivated boost improved the overall antibody titers [19–21]. These studies also revealed that the optimal responses occurred with a prime-boost interval between 12 and 24 weeks [20]. In some cases, the DNA prime was found to induce hemagglutinin (HA) stem-specific neutralizing antibodies, which could improve the breadth of the antibody response by eliciting a response against a stem domain that is highly conserved across multiple subtypes [19–21]. However, there were no significant increases in antibody responses as assessed by hemagglutination inhibition (HAI) in adults primed with either seasonal trivalent HA DNA vaccine or placebo followed by a boost with seasonal trivalent IIV (IIV3) [23]. The possibility that pre-existing immunity in the adult population limited the effect of the DNA prime warranted further evaluation in expanded age groups, including children and adolescents. In this first in the U.S. study of a DNA vaccine in a pediatric population, we compared the safety and immunogenicity of a seasonal influenza trivalent HA DNA vaccine prime-IIV3 boost (DNA-IIV3) regimen to a IIV3 prime-IIV3 boost (IIV3-IIV3) regimen.

## Materials and methods

The clinical trial protocol and CONSORT checklist are available in supporting information.

### Ethics statement

The trial was conducted at five clinical sites in the United States between June 2012 and July 2013. The protocol was reviewed for scientific, regulatory, and ethical requirements and was approved by the institutional review board at each site, including Cincinnati Children’s Hospital Medical Center, Vanderbilt University Medical Center, Saint Louis University, Emory Children’s Center, and Dartmouth Hitchcock Medical Center.

Written informed consent was obtained during enrollment from a parent or legal guardian with assent obtained from the minor child. The study followed guidelines for conducting clinical research with human subjects in accordance with 45 CFR Part 46 from the US Department of Health and Human Services [24], and US Food and Drug Administration regulations for investigational products, and principals expressed in the Declaration of Helsinki.

### Study design

The clinical trial was a Phase I, dose-escalation study in healthy children and adolescents to evaluate the safety, tolerability, and immunogenicity of a prime-boost regimen of the 2012/13 seasonal influenza trivalent HA DNA vaccine followed by a boost with licensed split virus 2012/13 trivalent inactivated influenza vaccine (IIV3). The comparator group received licensed 2012/13 IIV3 for both prime and boost.

**Fig 1:**
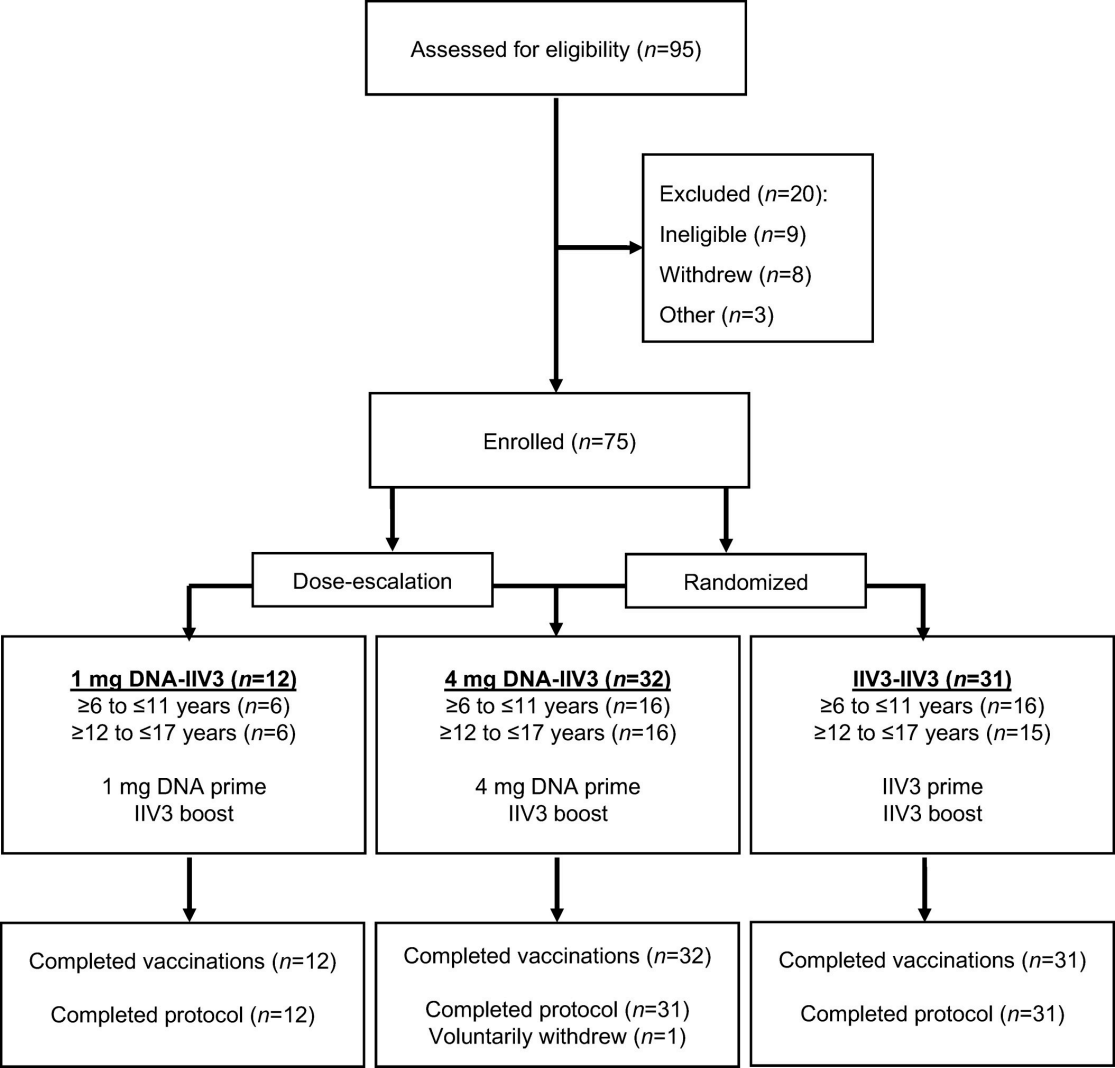
CONSORT diagram of study participants. All 75 study participants completed the scheduled vaccinations and 74 subjects completed the scheduled visits and were included in the analysis. The remaining subject withdrew after completing the vaccination schedule but prior to the four week post boost time point. This subject was analyzed for safety but was not included in the immunological end point analysis.

Seventy-five healthy adolescents and children ≥6 and ≤17 years of age were enrolled in the study between June and October 2012 (Fig 1). Participants were stratified by age (≥6 to ≤11 or ≥12 to ≤17 years old), with the older age group receiving each dose (1 mg or 4 mg) of the DNA vaccine first. Inclusion criteria required the participant to be in good general health with hematological and biochemical parameters within normal institutional limits and willing to have blood drawn throughout the trial. Exclusion criteria included weight less than 20 kg, recent receipt of immune-modulating medical products, prior receipt of 2012/13 influenza vaccine, contraindication to receiving influenza vaccine, or history of serious reactions to vaccination. After completion of the dose-escalation, the participants were randomized to receive either 4 mg DNA or IIV3 for the prime. All injections were given intramuscularly in the deltoid. DNA vaccines were administered using the needle-free jet injection device, Biojector 2000 (Bioject; Tualatin, OR, USA), while IIV3 was administered by needle and syringe.

Each participant received an IIV3 boost 18 (±2) weeks after the prime, with exceptions occurring in instances of local community outbreaks of influenza, only for participants who received DNA prime. Under this allowance, six participants in the DNA-IIV3 regimen had the IIV3 boost administered at either 14 (n = 5) or 15 weeks (n = 1). Participants with shortened intervals were included in the analysis.

Solicited local and systemic reactogenicity were assessed for seven days after each vaccination with the use of a diary card; adverse events were recorded for 28 days; and serious adverse events (SAE), new chronic medical conditions, or influenza-like illnesses (ILI) were monitored for the duration of the trial. No laboratory testing was done to distinguish influenza infections from illnesses with other etiologies. All adverse events were coded using the Medical Dictionary for Regulatory Activities.

The trial is registered with clinicaltrials.gov (NCT01609998).

### Vaccines

The investigational 2012/13 seasonal trivalent HA DNA vaccine (VRC-FLUDNA063-00-VP) consisted of three closed-circular plasmid DNA macromolecules (VRC-9328, VRC-3027, and VRC-2722) in equal amounts by weight, that express complete influenza HA sequences (A/California/04/2009 [A(H1N1)pdm09], A/Victoria/361/2011 (H3N2), and B/Wisconsin/1/2010, respectively) designed to closely match the 2012/13 licensed IIV3 (Table 1). The plasmid DNA was manufactured at the VRC Vaccine Pilot Plant operated by Leidos Biomedical Research, Inc. under current Good Manufacturing Practices, and formulated in phosphate buffered saline (PBS) to a final concentration of 4 mg/mL.

**Table 1:** Influenza antigens contained within the vaccines.

Vaccine	A(H1N1)pdm09	H3N2	B
**2012/13 trivalent HA DNA** **(prime only)**	A/California/04/2009	A/Victoria/361/2011	B/Wisconsin/1/2010
**2012/13 IIV3** **(prime and boost)**	A/California/07/2009	A/Victoria/361/2011-like	B/Wisconsin/1/2010-like: B/Texas/6/2011

The licensed split virus 2012/13 seasonal IIV3, Fluzone (Sanofi Pasteur, Inc., Swiftwater, PA, USA), contained the three influenza strains (A/California/07/2009 [A(H1N1)pdm09], A/Victoria/361/2011-like (H3N2), and B/Wisconsin/1/2010–like: B/Texas/6/2011) approved for the 2012/13 influenza season (Table 1).

### Randomization

Participants were randomized to the 4 mg DNA-IIV3 or IIV3 groups with equal allocation stratified by age and site. The randomization sequence was generated by the trial statistician in SAS using permuted blocked randomization with randomly selected block sizes of two or four. Upon enrollment, each participant’s randomized assignment was displayed in the electronic data entry system and vaccinations were administered in an open-label fashion.

### Immunogenicity assays

Blood samples were collected before each vaccination, at four weeks after each vaccination, and at 24 weeks after the boost to be tested for antibody responses by HAI and neutralization assays. Antibody titers were evaluated by HAI for six influenza strains, three each included in the 2012/13 and previous 2011/12 seasonal influenza vaccines to analyze both homologous and heterologous antibody responses (S1 Table). HAI assays were conducted at Southern Research, Inc. (Birmingham, AL) using validated methods [23]. Viruses were supplied by the CDC and amplified in embryonated eggs. Serum samples were incubated with three volumes of receptor destroying enzyme (RDE) and incubated at 37°C for approximately 18 hours, followed by 30–60 minutes ar 56°C. Dulbecco’s phosphate-buffered saline (DPBS) was then added to yield a 1:10 dilution of the original serum. Samples were tested in duplicate in V-bottom 96-well plates. Twenty-five μL of pretreated serum was added to the first row of the plate, and diluted 1:2 fold, to a final dilution of 1:1280. Twenty-five μL of virus, adjusted to 4 (hemagglutination untis) HAU/25 μL, was added to all the wells and incubated at room temperature for 30–60 minutes. Fifty μL of 0.5% turkey red blood cells was then added and plates were incubated for 30–60 minutes at room temperature. The titer was determined to be the last well that showed no agglutination and was expressed as the reciprocal of that dilution.

To study the neutralizing activity and breadth of the antibodies induced by vaccination, a panel of ten viruses comprised of influenza A subtypes (H1N1, H2N2, H3N2, H5N1, H7N9, and H9N2) and one influenza B virus was generated (S1 Table). The neutralization assays were run as previously described [19], using replication incompetent HA-pseudotyped viruses produced in 293T cells. The pseudotyped viruses expressed the HA antigen along with a luciferase reporter gene. Virus was incubated with sera samples for 45–90 minutes at 37°C before being added to the cells. Neutralization was quantitated by the relative decrease of the luciferase activity in infected 293A cells as compared to control sera, measured at 48–54 hours after infection. The dilution that resulted in 80% neutralization (ID_80_) was calculated relative to signal in the absence of sera, using five-parameter curve fitting. Assay controls included both neutralizing and non-neutralizing antibodies run concurrently with clinical samples. Neutralizing antibody responses against influenza B strains were further examined through a microneutralization assay. Both B/Brisbane/60/2008 and B/Wisconsin/1/2010 were analyzed (S1 Table). Each virus stock was grown in embryonated eggs, standardized to 10^3.3(± 0.08)^ 50% tissue culture infective doses (TCID_50_), and added to a microtiter plate. The starting concentration for the heat inactivated serum was 1:10, and two fold dilutions were made to a final dilution of 1:10240. The virus-serum mix was incubated at room temperature for 60 minutes and added to confluent MDCK cells. Samples were plated in quadruplicate and incubated at 37°C. Cytopathic effect was monitored and recorded at 72 and 96 hours. Neutralizing titer was defined as the reciprocal of the highest dilution of serum that completely neutralized infectivity of of the virus tested.

### Statistical analysis

The primary objective of the study was to assess the safety and tolerability of the DNA prime (at both 1 mg and 4 mg dosages) followed by an IIV3 boost in both children (≥6 to ≤11 years) and adolescents (≥12 to ≤17 years). The sample size was selected to obtain preliminary estimates of safety and immunogenicity in a pediatric population. The planned sample sizes provide over 90% chance to observe at least one event if the true rate is at least 0.21 in the 1 mg DNA-IIV3 group (n = 10) or 0.074 in the 4 mg DNA-IIV3 or IIV3-IIV3 groups (n = 30). The samples size of n = 30 provides 80% power to detect an absolute difference of 33%, assuming the reference group had a response rate of 50%.

The secondary objectives were to assess the seroconversion and magnitude of immune responses at four weeks post boost by HAI and neutralization assays. The 1 mg DNA prime group was included in the study primarily for safety and dose-escalation, and therefore the analysis of the antibody response focused on comparing the 4 mg DNA prime group to the IIV3 prime group. The HAI antibody titers for each group were displayed as the geometric mean titer (GMT). The seroconversion rates for HAI were determined per the FDA criteria of positivity as either a baseline (Day 0) titer < 1:10 and a post boost titer ≥ 1:40 or a baseline titer ≥ 1:10 and a minimum four-fold rise after boost [25], and the positive response rate for the neutralization assay was based on a four-fold rise in titer from baseline. The fold increase in HAI antibody titer was calculated by comparing the titer at four weeks post boost to the titer at baseline. Additionally, for neutralizing antibodies against each strain, the baseline corrected geometric mean fold ratio (GMFR) between groups and associated p-value was estimated using a linear regression model for log transformed titers, adjusting for baseline titer (log-transformed) under the assumption of heteroscedasticity. HAI or neutralizing antibody titers below the limit of detection for the assay (<10) were imputed for statistical purposes using a value of 5. Comparisons were made for each virus strain tested, and within each age stratum, using Fisher’s exact test for seroconversion rates, and t-test for response magnitude using log-transformed HAI titers or neutralization titers. Statistical significance was considered at a level of alpha = 0.05 without adjustment for multiple comparisons. Statistical analyses were performed in SAS 9.3 or higher (SAS Institute, Cary, NC).

Both nonparametric tests and t-tests were performed to analyze the results from this clinical trial. Both analyses resulted in consistent outcomes with one exception: a difference was observed in the GMFR comparison, due to the non-parametric tests not accounting for the differences in baseline titers (see S5 Table). The t-test comparisons were included in this publication since these were the originally planned analyses and also since these more accurately accounted for the baseline differences observed between groups.

## Results

### Study population

Seventy-five participants were enrolled in the study and all completed their vaccination regimens of either DNA prime followed by IIV3 boost (DNA-IIV3, n = 44) or IIV3 prime followed by IIV3 boost (IIV3-IIV3, n = 31). Overall, 74 participants (99%) completed the study protocol and one participant voluntarily withdrew from the study early (Fig 1). In total, there were 39 male (52%) and 36 female (48%) participants ≥6 to ≤17 years old (Table 2).

**Table 2:** Baseline demographics of participants by group and overall.

Characteristics	Treatment Group						Overall
	1 mg DNA-IIV3		4 mg DNA-IIV3		IIV3-IIV3		
	≥12 to ≤17 yrs (n = 6)	≥6 to ≤11 yrs (n = 6)	≥12 to ≤17 yrs (n = 16)	≥6 to ≤11 yrs (n = 16)	≥12 to ≤17 yrs (n = 16)	≥6 to ≤11 yrs (n = 15)	≥6 to ≤17 yrs (n = 75)
**Sex–no. (%)**							
**Male**	2 (33)	3 (50)	9 (56)	8 (50)	7 (44)	10 (67)	39 (52)
**Female**	4 (67)	3 (50)	7 (44)	8 (50)	9 (56)	5 (33)	36 (48)
**Age—years**^**a**^							
**Mean (S.D.)**	14.7 (1.2)	8.2 (1.5)	14.2 (1.5)	9.2 (1.3)	14.8 (1.9)	9.1 (1.6)	11.8 (3.2)
**Median [Range]**	15 [13,16]	9 [6,10]	14 [12,17]	9 [7,11]	15 [12,17]	9 [7,12]	12 [6,17]
**Race–no. (%)**							
**Black or African American**	2 (33)	0 (0)	2 (12)	0 (0)	4 (25)	4 (27)	12 (16)
**White**	4 (67)	4 (67)	11 (69)	15 (94)	11 (69)	11 (73)	56 (75)
**Multiracial**	0 (0)	2 (33)	3 (19)	1 (6)	1 (6)	0 (0)	7 (9)
**Ethnicity–no. (%)**							
**Non-Hispanic/Latino**	6 (100)	5 (83)	16 (100)	15 (94)	16 (100)	15 (100)	73 (97)
**Hispanic/Latino**	0 (0)	1 (17)	0 (0)	1 (6)	0 (0)	0 (0)	2 (3)
**Body Mass Index (BMI)**^**a**^							
**Mean (S.D.)**	21.6 (4.3)	17.3 (3.0)	24.1 (4.7)	18.0 (2.8)	23.4 (5.6)	18.2 (3.7)	20.7 (5.0)
**Range**	[18.3, 27.1]	[13.8, 22.7]	[18.0, 34.2]	[14.0, 24.5]	[16.2, 38.4]	[13.6, 28.2]	[13.6, 38.4]
**Influenza vaccinations in the previous 5 years–no. (%)**							
**>5 times**	0 (0)	0 (0)	3 (19)	3 (19)	4 (25)	2 (13)	12 (16)
**3–5 times**	3 (50)	1 (17)	4 (25)	12 (75)	4 (25)	9 (60)	33 (44)
**1–2 times**	1 (17)	3 (50)	8 (50)	0 (0)	5 (31)	3 (20)	20 (27)
**0 times**	2 (33)	2 (33)	1 (6)	1 (6)	3 (19)	1 (7)	10 (13)

^a^Age, along with height and weight (used for BMI), measured at date of prime vaccination.

Across all participants, 60% were immunized with seasonal influenza vaccine at least 3 times and 27% of participants 1–2 times in the five years prior to the start of the trial (Table 2). For the ≥12 to ≤17 years of age strata, 47% of participants received influenza vaccination at least 3 times and 37% received vaccine 1–2 times; while the majority (73%) of ≥6 to ≤11 years of age children received influenza vaccination at least 3 times, and 16% received vaccine 1–2 times.

### Vaccine reactogenicity and safety

The DNA-IIV3 regimen was safe and well tolerated. Three SAEs were reported; however, all were assessed as not related to study vaccinations. Overall, the investigational DNA vaccine was found to be associated with an increased frequency of local reactogenicity, with a significant difference in all solicited local reactogenicity symptoms between DNA prime and IIV3 prime (p<0.001 for pain/tenderness, swelling, and redness) (Table 3). These symptoms were treated with analgesics (ibuprofen or acetaminophen) if desired. However, all reactogenicity was mild or moderate in severity and resolved without sequelae, confirming safety of the DNA prime-IIV3 boost regimen.

**Table 3:** Summary of solicited reactogenicity.

Symptoms Intensity	DNA Prime (n = 44)	IIV3 Prime (n = 31)	IIV3 Boost^a^ (n = 75)
**Local reactogenicity, participants (%)**			
**PAIN/TENDERNESS**			
**None**	3 (6.8%)	13 (41.9%)	30 (40.0%)
**Mild**	41 (93.2%)	16 (51.6%)	42 (56.0%)
**Moderate**	0 (0.0%)	2 (6.5%)	3 (4.0%)
**SWELLING**			
**None**	29 (65.9%)	31 (100.0%)	73 (97.3%)
**Mild**	11 (25.0%)	0 (0.0%)	1 (1.3%)
**Moderate**	4 (9.1%)	0 (0.0%)	1 (1.3%)
**REDNESS**			
**None**	21 (47.7%)	26 (83.9%)	65 (86.7%)
**Mild**	22 (50.0%)	4 (12.9%)	7 (9.3%)
**Moderate**	1 (2.3%)	1 (3.2%)	3 (4.0%)
**ANY LOCAL SYMPTOM**			
**None**	3 (6.8%)	12 (38.7%)	29 (38.7%)
**Mild**	37 (84.1%)	16 (51.6%)	40 (53.3%)
**Moderate**	4 (9.1%)	3 (9.7%)	6 (8.0%)
**Systemic reactogenicity, participants (%)**			
**MALAISE**			
**None**	36 (81.8%)	25 (80.6%)	57 (76.0%)
**Mild**	8 (18.2%)	6 (19.4%)	13 (17.3%)
**Moderate**	0 (0.0%)	0 (0.0%)	5 (6.7%)
**MYALGIA**			
**None**	37 (84.1%)	28 (90.3%)	68 (90.7%)
**Mild**	7 (15.9%)	3 (9.7%)	6 (8.0%)
**Moderate**	0 (0.0%)	0 (0.0%)	1 (1.3%)
**HEADACHE**			
**None**	31 (70.5%)	25 (80.6%)	59 (78.7%)
**Mild**	11 (25.0%)	5 (16.1%)	13 (17.3%)
**Moderate**	2 (4.5%)	1 (3.2%)	3 (4.0%)
**CHILLS**			
**None**	42 (95.5%)	29 (93.5%)	70 (93.3%)
**Mild**	2 (4.5%)	1 (3.2%)	5 (6.7%)
**Moderate**	0 (0.0%)	1 (3.2%)	0 (0.0%)
**NAUSEA**			
**None**	40 (90.9%)	31 (100.0%)	67 (89.3%)
**Mild**	4 (9.1%)	0 (0.0%)	6 (8.0%)
**Moderate**	0 (0.0%)	0 (0.0%)	2 (2.7%)
**TEMPERATURE**			
**None**	43 (97.7%)	31 (100.0%)	73 (97.3%)
**Mild**	0 (0.0%)	0 (0.0%)	0 (0.0%)
**Moderate**	1 (2.3%)	0 (0.0%)	1 (1.3%)
**Severe**	0 (0.0%)	0 (0.0%)	1 (1.3%)
**ANY SYSTEMIC SYMPTOM**			
**None**	27 (61.4%)	21 (67.7%)	49 (65.3%)
**Mild**	15 (34.1%)	9 (29.0%)	19 (25.3%)
**Moderate**	2 (4.5%)	1 (3.2%)	6 (8.0%)
**Severe**	0 (0.0%)	0 (0.0%)	1 (1.3%)

Solicited reactogenicity was collected for 7 days after each vaccination. Each vaccine recipient was counted once at worst severity for any local and systemic parameter.

^a^IIV3 boost symptoms were consolidated for all vaccine regimens, as no differences were observed between groups.

**Fig 2:**
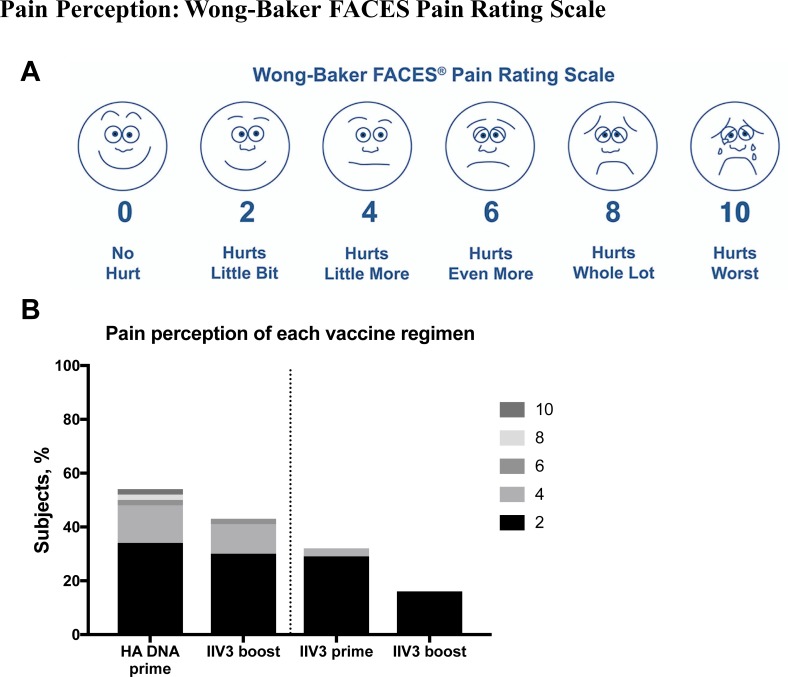
Overall pain perception following administration of vaccines. (A) The Wong-Baker Faces Pain Rating Scale for evaluation of pain perception following vaccination was shown to participants the same day (within an hour) and again at seven days post vaccination. (B) Overall pain perception of participants following both prime and boost. Age groups are combined and pain scores of 0 are not displayed.

To evaluate the pain perception in children, we used the Wong-Baker FACES Pain Rating Scale [26] (Fig 2A). Overall, the results demonstrated that administration of the DNA vaccine using a Biojector was associated with higher pain perception than administration of IIV3 via needle and syringe (Fig 2B). The younger children (≥6 to ≤11 years) chose higher pain readings than the older group (≥12 to ≤17 years) following DNA prime (p = 0.02), and this higher perception of pain was also projected on the IIV3 boost (p<0.001).

The frequency of participants with at least one unsolicited AE recorded following vaccination was similar across groups. The most frequent AEs were ILI and injection site bruising. Bruising occurred following DNA prime vaccination in three participants. Cumulatively, there were 17 participants with 19 total ILI cases recorded. Two unsolicited AEs assessed as possibly related to a study injection included a Grade 1 diarrhea with onset one day after a 1 mg DNA prime and a Grade 1 upper respiratory infection with onset four days after IIV3 boost. One ILI with onset at five days after IIV3 boost was recorded as severe because of self-reported fever being 104.1°F; however, the illness resolved within a day and did not meet criteria for reporting as an SAE. The incidence and severity of reported systemic reactogenicity was otherwise similar across all groups (Table 3).

### Immune response

Prior to vaccination, 61%-71% of children had a pre-existing HAI titer ≥ 1:10 and 36–48% had a pre-existing HAI titer ≥ 1:40 to both A/California/07/2009 [A(H1N1)pdm09] and A/Victoria/361/2011 (H3N2) while the pre-existing responses to B/Wisconsin/1/2010 were lower (23–26% of participants had an HAI titer of ≥ 1:10 and 10–13% of participants had an HAI titer ≥ 1:40) (S2 Table). These trends were similar for the 2011/12 vaccine strains tested (S2 Table).

The 1 mg DNA group was included in the trial primarily for safety, and therefore the antibody responses described are from the 4 mg DNA group. After prime, the IIV3 group displayed higher antibody responses by HAI than the DNA group (S2 and S3 Tables), and this was similar to previously published data on DNA influenza vaccines [27].

**Fig 3:**
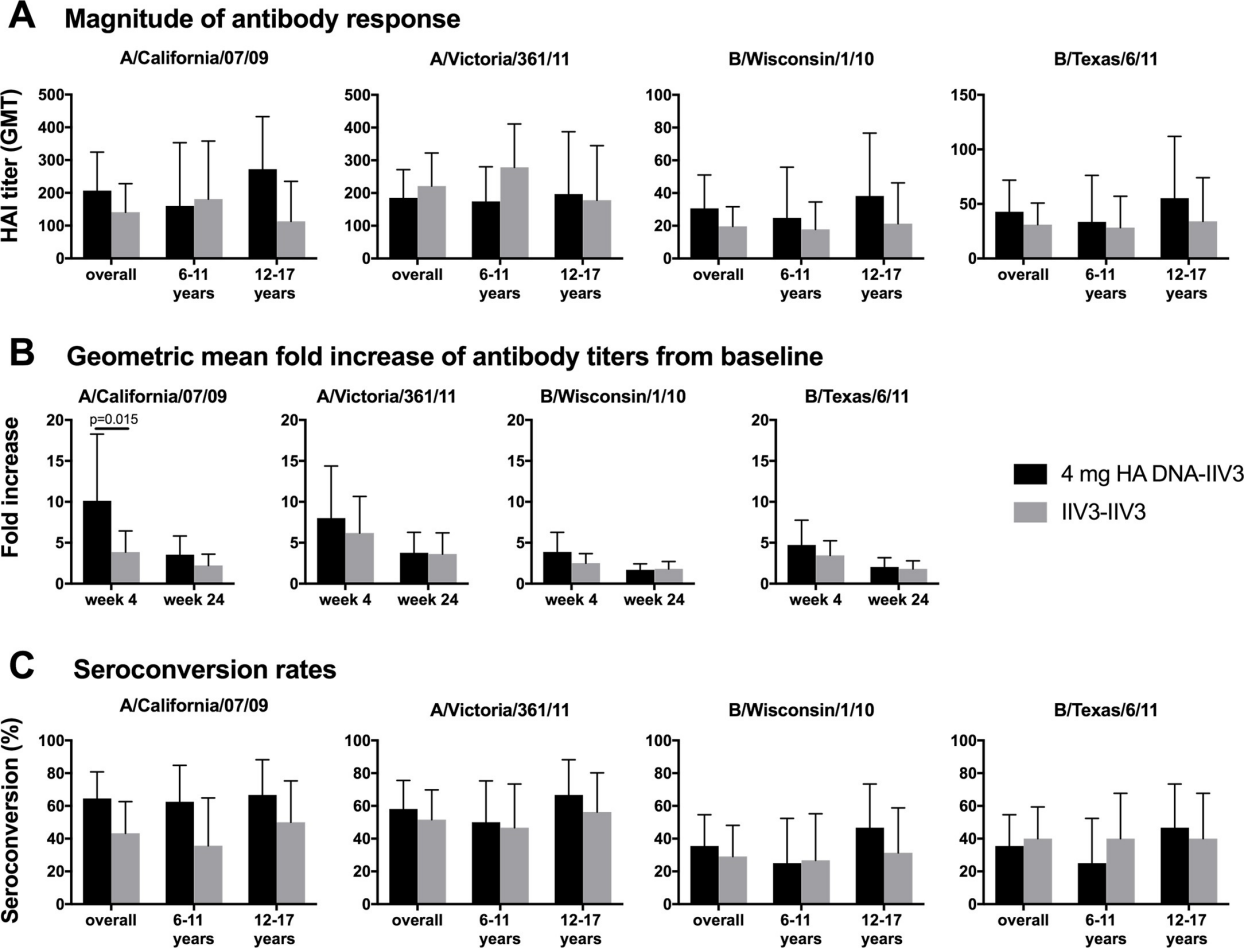
Antibody responses to the vaccine strains by age and group, as determined by HAI. The antibody responses for the 4 mg DNA-IIV3 and the IIV3-IIV3 regimens are displayed as group means based on the (A) magnitude of response at 4 weeks post boost, (B) geometric mean fold increase of titers from baseline at both 4 and 24 weeks post boost, and (C) seroconversion rates at 4 weeks post boost. DNA-IIV3 values shown in black, and IIV3-IIV3 in grey. Comparisons were made between the DNA-IIV3 and IIV3-IIV3 groups using Fisher’s exact test for serconversion rates, and t-test for response magnitude using log-transformed HAI titers.

The HAI antibody titers following the boost are summarized in S3 Table and Fig 3A. The 4 mg DNA-IIV3 regimen resulted in higher GMTs than the IIV3-IIV3 regimen at four weeks post boost for the H1 and influenza B strains, although the differences were not statistically significant. Higher fold increases of GMT compared to baseline titers were observed following 4 mg DNA-IIV3 for all tested influenza strains (Fig 3B, S4 Table). However, only the increase for A/California/07/2009 [A(H1N1)pdm09] was significant, with the 4 mg DNA-IIV3 group exhibiting an increase of 10.12 fold (95% CI = 5.60–18.27) compared to 3.86 fold (95% CI = 2.32–6.44) for the IIV3-IIV3 group (p = 0.015).

The seroconversion rates of participants at four weeks post boost by HAI are summarized in S2 Table and Fig 3C. The seroconversion rates were higher for the 4 mg DNA-IIV3 group compared to the IIV3-IIV3 group for all tested influenza strains except B/Texas/6/2011; however, the increase was not statistically significant between the groups for any of the influenza strains tested.

We were also interested in evaluating the neutralizing activity and breadth of the antibody responses following each vaccination regimen. At four weeks post boost, neutralizing antibody GMTs were similar between the 4 mg DNA-IIV3 and IIV3-IIV3 groups for all strains analyzed (S5 Table). Additional microneutralization assays were completed for influenza B, due to the high baseline and post boost GMTs observed in both groups against B/Brisbane/60/2008 in the pseudoneutralization assay (S5 Table). After adjusting for variable baseline titers the geometric mean fold increase, reported by geometric fold ratio (GMFR), was significantly higher at four weeks post boost in the 4 mg DNA-IIV3 compared with the IIV3-IIV3 groups for both H1N1 strains tested: A/New Caledonia/20/1999 (GMFR = 1.41, 95% CI = 1.10–1.81, p = 0.008) and A/South Carolina/1/1918 strains (GMFR = 1.55, 95% CI = 1.27–1.89, p<0.001) (Table 4). Seroconversion rates were also the same or higher in the 4 mg DNA-IIV3 group compared to the IIV3-IIV3 group across all strains tested (excluding A/Canada/720/2005), although these increases were not significant (Table 4).

**Table 4:** Baseline corrected geometric mean fold ratio and frequency of response by neutralization assays at four weeks post boost.

Treatment Group	Baseline Corrected Geometric Mean Fold Ratio (GMFR) (95% CI)	p-value for GMFR	Frequency of response (%) relative to baseline (95% CI)^b^
***A/New Caledonia/20/1999 (H1N1)***			
**DNA-IIV3**^**a**^	1.41 (1.10–1.81)	p = 0.008	9.7 (2.0–25.8)
**IIV3-IIV3**			0.0 (0.0–11.2)
***A/South Carolina/1/1918 (H1N1)***			
**DNA-IIV3**^**a**^	1.55 (1.27–1.89)	p<0.001	35.5 (19.2–54.6)
**IIV3-IIV3**			19.4 (7.5–37.5)
***A/Canada/720/2005 (H2N2)***			
**DNA-IIV3**^**a**^	0.96 (0.70–1.33)	p = 0.808	3.2 (0.1–16.7)
**IIV3-IIV3**			16.1 (5.5–33.7)
***A/Beijing/353/1989 (H3N2)***			
**DNA-IIV3**^**a**^	1.23 (0.87–1.73)	p = 0.239	32.3 (16.7–51.4)
**IIV3-IIV3**			16.1 (5.5–33.7)
***A/Hong Kong/1/1968 (H3N2)***			
**DNA-IIV3**^**a**^	1.21 (0.88–1.66)	p = 0.241	22.6 (9.6–41.1)
**IIV3-IIV3**			19.4 (7.5–37.5)
***A/Indonesia/05/2005 (H5N1)***			
**DNA-IIV3**^**a**^	0.96 (0.83–1.11)	p = 0.559	0.0 (0.0–11.2)
**IIV3-IIV3**			0.0 (0.0–11.2)
***A/Vietnam/1203/2004 (H5N1)***			
**DNA-IIV3**^**a**^	1.21 (0.88–1.66)	p = 0.227	12.9 (3.6–29.8)
**IIV3-IIV3**			6.5 (0.8–21.4)
***A/Anhui/1/2013 (H7N9)***			
**DNA-IIV3**^**a**^	1.04 (0.78–1.39)	p = 0.783	3.2 (0.1–16.7)
**IIV3-IIV3**			3.2 (0.1–16.7)
***A/Hong Kong/1073/1999 (H9N2)***			
**DNA-IIV3**^**a**^	1.28 (0.88–1.88)	p = 0.191	16.1 (5.5–33.7)
**IIV3-IIV3**			9.7 (2.0–25.8)
***B/Brisbane/60/2008***			
**DNA-IIV3**^**a**^	1.01 (0.74–1.38)	p = 0.928	16.1 (5.5–33.7)
**IIV3-IIV3**			16.1 (5.5–33.7)
***B/Brisbane/60/2008—microneutralization assay***			
**DNA-IIV3**^**a**^	0.94 (0.66–1.35)	p = 0.738	9.7 (2.0–25.8)
**IIV3-IIV3**			9.7 (2.0–25.8)
***B/Wisconsin/1/2010—microneutralization assay***			
**DNA-IIV3**^**a**^	1.18 (0.78–1.78)	p = 0.421	54.8 (36.0–72.7)
**IIV3-IIV3**			41.9 (24.5–60.9)

p values for GMFR comparison between regimens were determined by pairwise t-test.

^a^DNA injection at 4 mg.

^b^Positive response rate defined as four-fold increase over baseline.

In addition, in this small study we did not detect any statistically significant differences in the HAI or neutralization assays in immune responses between the age groups or between participants with different history of influenza vaccinations within age groups.

## Discussion

In previous influenza vaccine trials involving healthy adults, priming with a DNA vaccine and following with a monovalent inactivated boost proved more effective than an inactivated prime-boost regimen at establishing an antibody response against emerging subtypes of avian origin, including H5 and H7 [19, 21]. In a separate trial in healthy adults involving a DNA prime-IIV3 boost regimen with seasonal strains of influenza (H1, H3, and B), no significant improvement was observed compared to a placebo prime-IIV3 boost group, presumably due to pre-existing immune responses [23]. Therefore, we evaluated the DNA prime-IIV3 boost regimen in children and adolescents in an attempt to examine the safety and immunogenicity of the DNA prime-IIV3 boost in a more naïve population. Overall, the trivalent HA DNA prime-IIV3 boost regimen was safe and well tolerated. To our knowledge, this clinical trial is the first Phase I study in the U.S. involving a DNA vaccine in a healthy pediatric population.

To date, few studies have examined DNA vaccines in juvenile populations. One previous study conducted in Italy evaluated a therapeutic HIV DNA vaccine in HIV infected children between 6 and 16 years of age on highly active antiretroviral therapy (HAART). In the Phase II study, 20 children were enrolled and randomized to receive four intramuscular injections with the HIV DNA vaccine along with HAART, or to only receive continued HAART. The vaccine was reported as well tolerated with limited local reactogenicity and no severe systemic reactions [28, 29], further confirming DNA vaccines are a feasible strategy in pediatric populations.

One explanation for the difference in immune response observed between studies in healthy adults against avian subtypes of influenza where a significantly improved response was observed following priming with DNA [19, 21], and the single study investigating seasonal strains of influenza where no difference in titers was observed [23], was the high levels of pre-existing immunity to the seasonal strains in the adult population. This led to the possibility that an improved response following DNA-IIV3 vaccination could be observed in the more naïve pediatric population. However, in this pediatric trial we observed an immune response comparable to an IIV3-IIV3 regimen in our small number of participants. Moreover, high levels of baseline immunity against the seasonal vaccine strains were detected in this pediatric population with the majority of children having received at least one seasonal influenza immunization within the last five years (66% of ≥12 to ≤17 year olds, and 75% of ≥6 to ≤11 year olds). Baseline HAI titers were observed in 60–70% of participants for both H1 and H3 strains. Although these levels were lower than those observed in healthy adults [23], they could explain the lack of improvement compared to IIV3. The possibility exists that improved response would occur in children younger than this trial enrolled who are more naïve to seasonal influenza antigens.

Children under 5 years of age typically experience the most severe disease following influenza infection [6]. Vaccination of this more naïve age group with a DNA prime could be beneficial and provide protection against severe disease. However, performing a clinical trial with a novel vaccine platform in extremely young children or infants requires additional precautions. One such precaution includes conducting age de-escalation studies to first prove the safety of the vaccine in adults and adolescents [30]. Once such trials are completed, vaccination of this younger age group can be assessed in future trials.

While the majority of the antibody responses analyzed were similar among regimens, there was a trend towards increased responses against H1 viruses in the 4 mg DNA prime group. A significant fold increase in GMT was observed against the A(H1N1)pdm09 antigen, A/California/07/2009, by HAI. Significantly higher neutralizing fold increases were also detected against the two H1N1 viruses included in the panel (A/New Caledonia/20/1999 and A/South Carolina/1/1918). The lack of significance observed for the H3N2 and influenza B strains included in the vaccines could be attributable to the antigenic drifting of the seasonal H3N2 strain, A/Victoria/361/2011 that occurred during vaccine production [31], and the influenza B strain differing between the DNA prime and the 2012/13 IIV3 (B/Wisconsin/1/2010 vs B/Texas/6/2011). These mismatches between the DNA prime and IIV3 boost could have resulted in suboptimal responses for the DNA-IIV3 regimen. Although not analyzed in this study, DNA vaccines may also induce T cell responses that would not occur in IIV protein-based vaccine recipients, and could provide an additional benefit during future infections, even in cases of mismatched antigens [22].

The primary source of reactogenicity in this pediatric influenza vaccine trial may have originated from the administration method used for the DNA prime. This needle-free jet injection device (Biojector 2000) resulted in significantly higher reports of pain, swelling, and redness compared to needle and syringe administration in both age groups. This needle-free device has previously been linked to higher reports of pain in healthy adults [23]. However, the Biojector 2000 device used in this clinical study is an older version of needle-free technology that requires a CO_2_ cartridge. Newer, spring-powered versions of this technology exist that could possibly reduce, or possibly eliminate, these differences in reactogenicity [32] to make DNA vaccines even more acceptable in this age group. Non-invasive dermal patches are another novel possible route of administration for DNA vaccines that could be analyzed in future studies. The microneedle patches deliver vaccine antigens into the dermis of the skin before dissolving completely [33]. In a recent phase I study involving an IIV3 the microneedles were well tolerated and immunogenic [34].

Based on the studies in healthy children and adults to date [19–21, 23], a DNA prime-inactivated boost regimen appears most beneficial in a pandemic situation with novel subtypes, where little to no pre-existing immunity is present in the population. Based on the HAI titers following priming with DNA vaccine alone seen here and in other studies [27], an inactivated vaccine boost would still likely be required. In the event of a pandemic, DNA vaccines have the benefit of being rapidly developed and produced, allowing for quick priming of the population while an inactivated vaccine is being prepared. A DNA vaccine could therefore help with vaccine availability and rapid response rate for an emerging subtype, while remaining safe and well tolerated in all age groups.

## Supporting information

S1 CONSORT Checklist CONSORT checklist. (DOC)

S1 Table Influenza antigens used in the analysis. (DOCX)

S2 Table Seroconversion rates as measured by HAI. (DOCX)

S3 Table Magnitude of antibody responses as measured by HAI. (DOCX)

S4 Table Geometric fold increase as assessed by HAI at four weeks post boost. (DOCX)

S5 Table Geometric mean titers as measured by neutralization assay. (DOCX)

S1 Protocol VRC 702 clinical trial protocol. (PDF)
