# Supplementary material for: DNA vaccine priming for seasonal influenza vaccine in children and adolescents 6 to 17 years of age: A phase 1 randomized clinical trial
Source: PLoS One. 2018 Nov 2;13(11):e0206837. doi: 10.1371/journal.pone.0206837 (PMC6214651; doi:10.1371/journal.pone.0206837)
Supplement: S1 Table — (DOCX) [file pone.0206837.s002.docx]

**S1 Table. Influenza antigens used in the analysis**

| **HAI Assay Antigens** | **Neutralization Assay Antigens** |
| --- | --- |
| A/California/07/2009 A(H1N1)pdm09 | A/New Caledonia/20/1999 (H1N1) |
| A/Victoria/361/2011 (H3N2) | A/South Carolina/1/1918 (H1N1) |
| B/Wisconsin/1/2010 | A/Canada/720/2005 (H2N2) |
| B/Texas/6/2011 | A/Beijing/353/1989 (H3N2) |
| A/Perth/16/2009 (H3N2) | A/Hong Kong/1/1968 (H3N2) |
| B/Brisbane/60/2008 | A/Indonesia/05/2005 (H5N1) |
|  | A/Vietnam/1203/2004 (H5N1) |
|  | A/Anhui/1/2013 (H7N9) |
|  | A/Hong Kong/1073/1999 (H9N2) |
|  | B/Brisbane/60/2008^a^ |
|  | B/Wisconsin/1/2010^b^ |

^a^Antigen was analyzed in both neutralization assays.

^b^Antigen was analyzed in only the microneutralization assay.
