# Supplementary material for: DNA vaccine priming for seasonal influenza vaccine in children and adolescents 6 to 17 years of age: A phase 1 randomized clinical trial
Source: PLoS One. 2018 Nov 2;13(11):e0206837. doi: 10.1371/journal.pone.0206837 (PMC6214651; doi:10.1371/journal.pone.0206837)
Supplement: S2 Table — (DOCX) [file pone.0206837.s003.docx]

**S2 Table. Seroconversion rates as measured by HAI:  % of subjects (95% CI)**

|  | **Treatment Group** | | | | | | |
| --- | --- | --- | --- | --- | --- | --- | --- |
|  | **All ages** | | | **6-11 years** | | **12-17 years** | |
|  | **DNA-IIV3^a^ (n=31)** | **IIV3-IIV3 (n=31)** | **p value** | **DNA-IIV3^a^**  **(n=16)** | **IIV3-IIV3**  **(n=15)** | **DNA-IIV3^a^**  **(n=15)** | **IIV3-IIV3**  **(n=16)** |
| ***A/California/07/2009 A(H1N1)pdm09*** | | | | | | | |
| **≥1:10 at baseline** | 61.3  (42.2-78.2) | 71.0  (52.0-85.8) |  | 62.5  (35.4-84.8) | 60.0  (32.3-83.7) | 60.0  (32.3-83.7) | 81.3  (54.4-96.0) |
| **≥1:40 at baseline** | 35.5  (19.2-54.6) | 45.2  (27.3-64.0) |  | 37.5  (15.2-64.6) | 46.7  (21.3-73.4) | 33.3  (11.8-61.6) | 43.8  (19.8-70.1) |
| **positive pre-boost^b^** | 0.0  (0.0-11.2) | 38.7  (21.8-57.8) |  | 0.0  (0.0-20.6) | 40.0  (16.3-67.7) | 0.0  (0.0-21.8) | 37.5  (15.2-64.6) |
| **positive four weeks post-boost^b^** | 64.5  (45.4-80.8) | 43.3  (25.5-62.6) | 0.126 | 62.5  (35.4-84.8) | 35.7  (12.8- 64.9) | 66.7  (38.4-88.2) | 50.0  (24.7-75.3) |
| ***A/Victoria/361/2011 (H3N2)*** | | | | | | | |
| **≥1:10 at baseline** | 61.3  (42.2-78.2) | 71.0  (52.0-85.8) |  | 75.0  (47.6-92.7) | 73.3  (44.9-92.2) | 46.7  (21.3-73.4) | 68.8  (41.3-89.0) |
| **≥1:40 at baseline** | 38.7  (21.8-57.8) | 48.4  (30.2-66.9) |  | 37.5  (15.2-64.6) | 53.3  (26.6-78.7) | 40.0  (16.3-67.7) | 43.8  (19.8-70.1) |
| **positive pre-boost^b^** | 3.2  (0.1-16.7) | 41.9  (24.5-60.9) |  | 6.3  (0.2-30.2) | 40.0  (16.3-67.7) | 0.0  (0.0-21.8) | 43.8  (19.8-70.1) |
| **positive four weeks post-boost^b^** | 58.1  (39.1-75.5) | 51.6  (33.1-69.8) | 0.799 | 50.0  (24.7-75.3) | 46.7  (21.3-73.4) | 66.7  (38.4-88.2) | 56.3  (29.9-80.2) |
| ***B/Wisconsin/1/2010*** | | | | | | | |
| **≥1:10 at baseline** | 22.6  (9.6-41.1) | 25.8  (11.9-44.6) |  | 18.8  (4.0-45.6) | 20.0  (4.3-48.1) | 26.7  (7.8-55.1) | 31.3  (11.0-58.7) |
| **≥1:40 at baseline** | 9.7  (2.0-25.8) | 12.9  (3.6-29.8) |  | 6.3  (0.2-30.2) | 13.3  (1.7-40.5) | 13.3  (1.7-40.5) | 12.5  (1.6-38.3) |
| **positive pre-boost^b^** | 0.0  (0.0-11.2) | 22.6  (9.6-41.1) |  | 0.0  (0.0-20.6) | 26.7  (7.8-55.1) | 0.0  (0.0-21.8) | 18.8  (4.0-45.6) |
| **positive four weeks post-boost^b^** | 35.5  (19.2-54.6) | 29.0  (14.2-48.0) | 0.786 | 25.0  (7.3-52.4) | 26.7  (7.8-55.1) | 46.7  (21.3-73.4) | 31.3  (11.0-58.7) |
| ***B/Texas/6/2011*** | | | | | | | |
| **≥1:10 at baseline** | 25.8  (11.9-44.6) | 25.8  (11.9-44.6) |  | 18.8  (4.0-45.6) | 33.3  (11.8-61.6) | 33.3  (11.8-61.6) | 18.8  (4.0-45.6) |
| **≥1:40 at baseline** | 12.9  (3.6-29.8) | 12.9  (3.6-29.8) |  | 6.3  (0.2-30.2) | 13.3  (1.7-40.5) | 20.0  (4.3-48.1) | 12.5  (1.6-38.3) |
| **positive pre-boost^b^** | 0.0  (0.0-11.2) | 22.6  (9.6-41.1) |  | 0.0  (0.0-20.6) | 26.7  (7.8-55.1) | 0.0  (0.0-21.8) | 18.8  (4.0-45.6) |
| **positive four weeks post-boost^b^** | 35.5  (19.2-54.6) | 40.0  (22.7-59.4) | 0.508 | 25.0  (7.3-52.4) | 40.0  (16.3-67.7) | 46.7  (21.3-73.4) | 40.0  (16.3-67.7) |
| ***A/Perth/16/2009 (H3N2) – previous 2011/12 seasonal strain*** | | | | | | | |
| **≥1:10 at baseline** | 63.3  (43.9-80.1) | 67.7  (48.6-83.3) |  | 80.0  (51.9-95.7) | 66.7  (38.4-88.2) | 46.7  (21.3-73.4) | 68.8  (41.3-89.0) |
| **≥1:40 at baseline** | 40.0  (22.7-59.4) | 51.6  (33.1-69.8) |  | 40.0  (16.3-67.7) | 53.3  (26.6-78.7) | 40.0  (16.3-67.7) | 50.0  (24.7-75.3) |
| **positive pre-boost^b^** | 0.0  (0.0-11.6) | 45.2  (27.3-64.0) |  | 0.0  (0.0-21.8) | 46.7  (21.3-73.4) | 0.0  (0.0-21.8) | 43.8  (19.8-70.1) |
| **positive four weeks post-boost^b^** | 63.3  (43.9-80.1) | 51.6  (33.1-69.8) | 0.440 | 60.0  (32.3-83.7) | 46.7  (21.3-73.4) | 66.7  (38.4-88.2) | 56.3  (29.9-80.2) |
| ***B/Brisbane/60/2008 – previous 2011/12 seasonal strain*** | | | | | | | |
| **≥1:10 at baseline** | 29.0  (14.2-48.0) | 45.2  (27.3-64.0) |  | 25.0  (7.3-52.4) | 53.3  (26.6-78.7) | 33.3  (11.8-61.6) | 37.5  (15.2-64.6) |
| **≥1:40 at baseline** | 19.4  (7.5-37.5) | 29.0  (14.2-48.0) |  | 6.3  (0.2-30.2) | 40.0  (16.3-67.7) | 33.3  (11.8-61.6) | 18.8  (4.0-45.6) |
| **positive pre-boost^b^** | 0.0  (0.0-11.9) | 12.9  (3.6-29.8) |  | 0.0  (0.0-21.8) | 20.0  (4.3-48.1) | 0.0  (0.0-23.2) | 6.3  (0.2-30.2) |
| **positive four weeks post-boost^b^** | 20.0  (7.7-38.6) | 12.9  (3.6-29.8) | 0.795 | 18.8  (4.0-45.6) | 20.0  (4.3-48.1) | 21.4  (4.7-50.8) | 6.3  (0.2-30.2) |

**^a^DNA injection at 4 mg**

**^b^Positive immune response defined as four-fold increase if baseline titer is ≥1:10, or ≥1:40 if baseline titer is <1:10. Comparisons of antibody responses between regimens are shown for the 4 week post boost time point.**
