# Supplementary material for: DNA vaccine priming for seasonal influenza vaccine in children and adolescents 6 to 17 years of age: A phase 1 randomized clinical trial
Source: PLoS One. 2018 Nov 2;13(11):e0206837. doi: 10.1371/journal.pone.0206837 (PMC6214651; doi:10.1371/journal.pone.0206837)
Supplement: S3 Table — (DOCX) [file pone.0206837.s004.docx]

**S3 Table. Magnitude of antibody responses by age groups as measured by HAI: GMT (95% CI)**

|  | **Treatment Group** | | | | | | |
| --- | --- | --- | --- | --- | --- | --- | --- |
|  | **All ages** | | | **6-11 years** | | **12-17 years** | |
|  | **DNA-IIV3^a^**  **(n=31)** | **IIV3-IIV3 (n=31)** | **p value^b^** | **DNA-IIV3^a^**  **(n=16)** | **IIV3-IIV3**  **(n=15)** | **DNA-IIV3^a^**  **(n=15)** | **IIV3-IIV3**  **(n=16)** |
| ***A/California/07/2009 A(H1N1)pdm09*** | | | | | | | |
| **baseline** | 20.5  (12.3-34.0) | 34.2  (19.5-60.0) |  | 17.9  (9.1-35.5) | 34.0  (13.2-87.6) | 23.5  (10.2-54.4) | 34.4  (16.3-72.6) |
| **pre-boost** | 22.9  (14.4-36.3) | 109.4  (65.8-181.8) |  | 19.6  (9.6-39.9) | 130.0  (60.8-277.9) | 27.0  (13.9-52.5) | 93.1 (43.7-198.4) |
| **Four weeks post boost** | 206.9  (131.9-324.5) | 140.9  (87.1-228.0) | 0.238 | 160.0  (72.5-353.3) | 181.1  (91.5-358.3) | 272.2  (171.2-432.8) | 113.1  (54.4-235.4) |
| ***A/Victoria/361/2011 (H3N2)*** | | | | | | | |
| **baseline** | 23.1 (14.1 - 37.9) | 35.8 (20.2 - 63.5) |  | 25.4 (14.3 - 45.1) | 43.9 (16.5 - 116.9) | 20.9 (8.6 - 51.2) | 29.5 (14.2 - 61.4) |
| **pre-boost** | 27.4 (17.4 - 43.0) | 129.4 (73.9 - 226.6) |  | 30.8 (17.2 - 55.2) | 175.5 (86.4 - 356.5) | 24.1 (11.1 - 52.2) | 97.2 (38.9 - 243.3) |
| **Four weeks post boost** | 185.0 (126.0 - 271.6) | 221.3 (152.0 - 322.1) | 0.499 | 174.5 (108.6 - 280.4) | 278.6 (188.7 - 411.2) | 197.0 (100.2 - 387.2) | 178.3 (92.2 - 344.9) |
| ***B/Wisconsin/1/2010*** | | | | | | | |
| **baseline** | 7.9 (5.8 - 10.8) | 7.8 (5.8 - 10.6) |  | 7.1 (5.0 - 10.0) | 7.1 (4.7 - 10.6) | 8.9 (5.1 - 15.7) | 8.6 (5.3 - 14.0) |
| **pre-boost** | 7.6 (5.9 - 9.9) | 17.5 (11.0 - 27.9) |  | 7.1 (4.9 - 10.1) | 17.4 (9.2 - 32.9) | 8.3 (5.4 - 12.8) | 17.6 (8.3 - 37.3) |
| **Four weeks post boost** | 30.6 (18.4 - 51.0) | 19.6 (12.1 - 31.6) | 0.198 | 24.8 (11.1 - 55.8) | 17.8 (9.2 - 34.5) | 38.2 (19.1 - 76.6) | 21.3 (9.9 - 46.2) |
| ***B/Texas/6/2011*** | | | | | | | |
| **baseline** | 9.0 (6.1 - 13.4) | 8.8 (6.0 - 13.0) |  | 7.5 (4.9 - 11.7) | 9.5 (5.4 - 17.0) | 11.0 (5.4 - 22.4) | 8.2 (4.6 - 14.8) |
| **pre-boost** | 8.7 (6.3 - 12.1) | 19.8 (11.6 - 33.9) |  | 7.9 (5.0 - 12.3) | 21.4 (9.9 - 46.2) | 9.8 (5.7 - 16.7) | 18.3 (7.9 - 42.5) |
| **Four weeks post boost** | 42.8 (25.5 - 71.8) | 31.0 (19.0 - 50.8) | 0.362 | 33.6 (14.8 - 76.2) | 28.3 (14.0 - 57.1) | 55.3 (27.3 - 111.9) | 34.0 (15.6 - 74.0) |
| ***A/Perth/16/2009 (H3N2) – previous 2011/12 seasonal strain*** | | | | | | | |
| **baseline** | 23.2 (13.9 - 38.9) | 30.6 (16.9 - 55.3) |  | 26.4 (14.7 - 47.4) | 36.5 (13.4 - 99.0) | 20.5 (8.1 - 51.6) | 25.9 (11.9 - 56.4) |
| **pre-boost** | 27.4 (17.2 - 43.5) | 114.4 (69.6 - 188.1) |  | 31.5 (17.6 - 56.5) | 145.9 (75.2 - 283.0) | 23.5 (10.6 - 52.4) | 91.1 (41.2 - 201.7) |
| **Four weeks post boost** | 148.0 (102.3 - 214.1) | 167.3 (113.4 - 247.0) | 0.642 | 146.7 (91.7 - 234.7) | 211.1 (142.7 - 312.4) | 149.3 (78.6 - 283.5) | 134.5 (67.4 - 268.5) |
| ***B/Brisbane/60/2008 – previous 2011/12 seasonal strain*** | | | | | | | |
| **baseline** | 9.4 (6.5 - 13.5) | 13.4 (8.7 - 20.6) |  | 8.2 (5.1 - 13.2) | 16.6 (8.4 - 32.9) | 10.7 (5.7 - 20.0) | 10.9 (6.0 - 20.0) |
| **pre-boost** | 8.2 (5.7 - 11.7) | 16.0 (9.7 - 26.4) |  | 6.8 (4.3 - 10.6) | 21.9 (10.3 - 46.5) | 10.0 (5.4 - 18.5) | 11.9 (5.8 - 24.4) |
| **Four weeks post boost** | 16.2 (9.9 - 26.7) | 16.0 (9.8 - 26.2) | 0.964 | 13.3 (7.4 - 23.9) | 23.0 (10.5 - 50.3) | 20.5 (8.3 - 50.6) | 11.4 (6.0 - 21.7) |

**^a^DNA injection at 4 mg**

**^b^Comparisons of antibody responses between regimens for all ages are shown for the 4 week post boost time point**
