# Supplementary material for: DNA vaccine priming for seasonal influenza vaccine in children and adolescents 6 to 17 years of age: A phase 1 randomized clinical trial
Source: PLoS One. 2018 Nov 2;13(11):e0206837. doi: 10.1371/journal.pone.0206837 (PMC6214651; doi:10.1371/journal.pone.0206837)
Supplement: S4 Table — (DOCX) [file pone.0206837.s005.docx]

**S4 Table. Geometric fold increase as assessed by HAI at four weeks post boost**

| **Treatment Group** | **Geometric mean fold increase from baseline (95% CI)** | **p value** |
| --- | --- | --- |
| ***A/California/07/2009 A(H1N1)pdm09*** | | |
| **4 mg DNA-IIV3** | 10.12 (5.60,18.27) | 0.015 |
| **IIV3-IIV3** | 3.86 (2.32,6.44) |  |
| ***A/Victoria/361/2011 (H3N2)*** | | |
| **4 mg DNA-IIV3** | 8.00 (4.45,14.38) | 0.514 |
| **IIV3-IIV3** | 6.19 (3.59,10.66) |  |
| ***B/Wisconsin/1/2010*** | | |
| **4 mg DNA-IIV3** | 3.87 (2.38,6.28) | 0.155 |
| **IIV3-IIV3** | 2.50 (1.70,3.67) |  |
| ***B/Texas/6/2011*** | | |
| **4 mg DNA-IIV3** | 4.73 (2.89,7.75) | 0.318 |
| **IIV3-IIV3** | 3.44 (2.27,5.22) |  |
| ***A/Perth/16/2009 (H3N2) – previous 2011/12 seasonal strain*** | | |
| **4 mg DNA-IIV3** | 6.35 (3.83,10.53) | 0.672 |
| **IIV3-IIV3** | 5.47 (3.30,9.07) |  |
| ***B/Brisbane/60/2008 – previous 2011/12 seasonal strain*** | | |
| **4 mg DNA-IIV3** | 1.82 (1.19,2.79) | 0.154 |
| **IIV3-IIV3** | 1.20 (0.79,1.82) |  |
