# Supplementary material for: DNA vaccine priming for seasonal influenza vaccine in children and adolescents 6 to 17 years of age: A phase 1 randomized clinical trial
Source: PLoS One. 2018 Nov 2;13(11):e0206837. doi: 10.1371/journal.pone.0206837 (PMC6214651; doi:10.1371/journal.pone.0206837)
Supplement: S5 Table — (DOCX) [file pone.0206837.s006.docx]

**S5 Table. Geometric Mean Titers by Neutralization Assay as measured by neutralization assay**

| **Treatment Group** | **GMT at baseline (95% CI)** | **GMT at four weeks post boost (95% CI)** |
| --- | --- | --- |
| ***A/New Caledonia/20/1999 (H1N1)*** | | |
| **DNA-IIV3^a^** | 82.5 (55.4-123.0) | 122.8 (86.0-175.3) |
| **IIV3-IIV3** | 74.6 (57.1-97.4) | 74.1  (51.8-106.1) |
| ***A/South Carolina/1/1918 (H1N1)*** | | |
| **DNA-IIV3^a^** | 119.2 (76.1-186.7) | 427.9 (249.5-733.9) |
| **IIV3-IIV3** | 231.8 (152.3-352.7) | 448.5 (297.4-676.6) |
| ***A/Canada/720/2005 (H2N2)*** | | |
| **DNA-IIV3^a^** | 6.3 (5.4-7.5) | 7.6 (6.1-9.5) |
| **IIV3-IIV3** | 6.1 (5.1-7.3) | 7.8 (6.1-10.1) |
| ***A/Beijing/353/1989 (H3N2)*** | | |
| **DNA-IIV3^a^** | 86.5 (60.8-123.0) | 247.5 (176.1-348.0) |
| **IIV3-IIV3** | 111.3 (73.3-169.0) | 233.8 (165.3-330.5) |
| ***A/Hong Kong/1/1968 (H3N2)*** | | |
| **DNA-IIV3^a^** | 29.4 (22.1-38.9) | 59.9 (42.7-84.0) |
| **IIV3-IIV3** | 32.4 (22.6-46.4) | 57.5 (43.3-76.4) |
| ***A/Indonesia/05/2005 (H5N1)*** | | |
| **DNA-IIV3^a^** | 5.6 (4.9-6.4) | 5.6 (5.0-6.3) |
| **IIV3-IIV3** | 5.6 (5.0-6.4) | 5.6 (4.9-6.3) |
| ***A/Vietnam/1203/2004 (H5N1)*** | | |
| **DNA-IIV3^a^** | 23.3 (15.5-35.2) | 32.7 (22.0-48.7) |
| **IIV3-IIV3** | 20.5 (14.5-28.9) | 26.0 (18.6-36.3) |
| ***A/Anhui/1/2013 (H7N9)*** | | |
| **DNA-IIV3^a^** | 6.1 (4.8-7.6) | 7.9 (6.0-10.4) |
| **IIV3-IIV3** | 5.8 (5.1-6.5) | 7.3 (5.6-9.5) |
| ***A/Hong Kong/1073/1999 (H9N2)*** | | |
| **DNA-IIV3^a^** | 9.5 (7.1-12.7) | 17.3 (13.0-23.0) |
| **IIV3-IIV3** | 12.1 (9.2-15.8) | 14.6 (10.7-19.9) |
| ***B/Brisbane/60/2008*** | | |
| **DNA-IIV3^a^** | 419.3 (300.4-585.2) | 740.5 (531.5-1031.7) |
| **IIV3-IIV3** | 680.9 (470.0-986.5) | 1022.4 (766.4-1364.0) |
| ***B/Brisbane/60/2008 - microneutralization assay*** | | |
| **DNA-IIV3^a^** | 57.8 (39.2 - 85.1) | 97.5 (66.0 - 143.9) |
| **IIV3-IIV3** | 100.8 (64.2 - 158.4) | 150.1 (101.3 - 222.5) |
| ***B/Wisconsin/1/2010 - microneutralization assay*** | | |
| **DNA-IIV3^a^** | 51.1 (36.7 - 71.2) | 231.4 (160.0 - 334.7) |
| **IIV3-IIV3** | 71.1 (52.8 - 95.7) | 230.4 (171.8 - 309.0) |

**^a^DNA injection at 4 mg**
