# Supplementary material for: DNA vaccine priming for seasonal influenza vaccine in children and adolescents 6 to 17 years of age: A phase 1 randomized clinical trial
Source: PLoS One. 2018 Nov 2;13(11):e0206837. doi: 10.1371/journal.pone.0206837 (PMC6214651; doi:10.1371/journal.pone.0206837)
Supplement: S1 Protocol — (PDF) [file pone.0206837.s007.pdf]

An Open-Label, Dose-Escalation, Phase I Study of the Safety, Tolerability and Immunogenicity of the Prime-Boost Regimen of the Investigational 2012/13 Seasonal Influenza DNA Vaccine, VRC-FLUDNA063-00-VP, Followed by the 2012/2013 Seasonal Influenza Trivalent Inactivated Vaccine (TIV) Compared to TIV Prime-TIV Boost in Children and Adolescents Ages 6-17 Years

Protocol VRC 702

Sponsored by:
National Institute of Allergy and Infectious Diseases (NIAID)
Vaccine Research Center (VRC)
Bethesda, Maryland

BB-IND 15062– held by VRC, NIAID, NIH

NIAID Funding Mechanism:
Contract # HHSN272201000049I

Clinical Trial Management:
Universal Influenza Clinical Program Support Center (UNI-CPSC)
The EMMES Corporation
Rockville, MD 20850

Version 2.0
July 18, 2012

Confidentiality Statement

This document is confidential and is to be distributed for review only to investigators, potential investigators, consultants, study staff, and applicable independent ethics committees or institutional review boards. The contents of this document shall not be disclosed to others without written authorization from NIAID (or others, as applicable), unless it is necessary to obtain informed consent from potential study participants.

Statement of Compliance

The trial will be conducted in compliance with the protocol, the applicable regulatory requirements including but not limited to the U.S. Code of Federal Regulations applicable to clinical studies (45 CFR 46, 21 CFR including parts 50 and 56 concerning informed consent and IRB regulations, and 21 CFR 312 concerning Investigational New Drug (IND) application), International Conference on Harmonisation Good Clinical Practice (ICH-GCP) guidance, and the NIAID Clinical Terms of Contract Award. The site will hold a current Federal Wide Assurance (FWA) issued by OHRP for federally funded research. Completion of Protection of Human Subjects Training will be required for all study personnel in accordance with NIH policy.

TABLE OF CONTENTS

INVESTIGATOR OF RECORD PROTOCOL SIGNATURE PAGE...6

ABBREVIATIONS 7

PRÉCIS 9

1 Introduction..11

1.1 Influenza Biology, Natural History and Vaccines 11
1.2 Rationale for Seasonal Influenza DNA Vaccine Prime-Inactivated Boost 12
1.3 Rationale for Influenza DNA Vaccine Testing in Children.......................................13
1.4 Previous Human Experience with VRC DNA Vaccines 14
1.4.1 Plasmid DNA Vaccines Developed by VRC, NIAID, NIH 14
1.4.2 Influenza DNA Vaccines Developed by VRC, NIAID, NIH 14
1.5 Experience with DNA Vaccines in Children 18
1.6 Assessment of Immunogenicity...18

2 Study Vaccines...19

2.1 Formulation and Manufacturing of VRC-FLUDNA063-00-VP 19
2.2 Formulation and Manufacturing of Seasonal Trivalent Influenza Vaccine................20
2.3 Pre-Clinical Studies of HA DNA Vaccines 20

3 Study Objectives 20

3.1 Primary objective:...20
3.2 Secondary objective:...20
3.3 Exploratory Analyses:...21

4 Study Design..21

4.1 Study Population...22
4.1.1 Inclusion Criteria 22
4.1.2 Exclusion Criteria 23
4.2 Study Schedule..24
4.2.1 Pre-enrollment... 24
4.2.2 Screening Visit(s)... 24
4.2.3 Enrollment Visit... 24
4.2.4 Administration of Study Injections:... 25
4.2.5 Blood Sample Collection 26
4.2.6 Concomitant Medications and Procedures... 26
4.2.7 Protocol Considerations Related to the Licensed TIV................................. 26
4.3 Criteria for Dose Escalation..27
4.4 Criteria for Discontinuing Subject Participation...27
4.5 Pausing the Study..28
4.5.1 Criteria for Pausing the Study.. 28
4.5.2 Plan for Review of Pauses and Resuming Rules 28

5 Safety and Adverse Events 29

5.1 Adverse Events 29
5.1.1 Adverse Event (AE) Definition 29

5.1.2 Adverse Event Reporting in the Study Database 29
5.2 Serious Adverse Events 29
5.2.1 Serious Adverse Event Definition 29
5.2.2 Reporting Serious Adverse Events to the IND Sponsor 30
5.2.3 IND Sponsor Reporting to the FDA 30
5.3 Reporting to Site IRBs 31
5.4 Data and Safety Monitoring Board 31
6 Statistical Considerations and Sample Analysis 31
6.1 Overview 31
6.2 Objectives 31
6.3 Endpoints 31
6.3.1 Primary Endpoints: Safety 31
6.3.2 Secondary Endpoints: Immune Responses 32
6.3.3 Exploratory Endpoints: Immune Responses 32
6.4 Sample Size and Accrual 32
6.4.1 Power Calculations for Evaluation of Safety 33
6.4.2 Power Calculations for Evaluation of Immune Responses 34
6.4.3 Power Calculations for Immunogenicity Comparisons 35
6.5 Statistical Analysis 36
6.5.1 Analysis Variables 36
6.5.2 Baseline Characteristics 36
6.5.3 Safety Analysis 36
6.5.4 Analysis of Immune Responses 36
6.5.5 When Analyses May Be Performed 37
6.5.6 Randomization of Treatment Assignments 37
7 Pharmacy and Vaccine Administration Procedures 38
7.1 Study Agents 38
7.2 Study Agent Presentation and Storage 38
7.2.1 Study Agent Labels 38
7.2.2 Study Agent Storage 38
7.3 Preparation of Study Agent for Injection 38
7.3.1 Preparation of VRC-FLUDNA063-00-VP 39
7.3.2 Preparation of 2012-2013 TIV Injection 39
7.4 Study Agent Accountability 39
7.4.1 Documentation 39
7.4.2 Disposition 39
8 Human Subjects Protection 39
8.1 Institutional Review Board 39
8.2 Subject Recruitment and Enrollment 40
8.2.1 Participation of Children 40
8.3 Informed Consent 40
8.3.1 Minor Assent 41
8.4 Subject Confidentiality 41
8.5 Risks and Benefits 41
8.5.1 Risks of Blood Collections: 41

8.5.2 Risks of the DNA Vaccine: 41
8.5.3 Risks of the Seasonal TIV Vaccine: 42
8.5.4 Other Risks: 42
8.5.5 Benefits: 43
8.6 Plan for Use and Storage of Biological Samples 43
8.7 Compensation 43
8.8 Safety Monitoring 44
8.8.1 Protocol Safety Review Team 44
8.8.2 DSMB 44
9 Administration and Legal Obligations 44
9.1 Protocol Initiation, Amendments and Termination 44
9.2 Study Documentation and Study Records Retention 44
9.3 Data Collection and Protocol Monitoring 45
9.3.1 Data Capture Methods 45
9.3.2 Source Documents and Access to Source Data/Documents 45
9.3.3 Protocol Monitoring 45
9.4 Language 45
9.5 Policy Regarding Research-Related Injuries 45
10 References 46
Appendix I: Informed Consent Form for Parent/Guardian and Minor Assent Templates 49
Appendix II: Contact Information 61
Appendix III: Schedule of Evaluations 64
Appendix IV: Assessment of Relationship to Vaccine and Adverse Event Severity Grading 66

INVESTIGATOR OF RECORD PROTOCOL SIGNATURE PAGE
Study VRC 702

An Open-Label, Dose-Escalation, Phase I Study of the Safety, Tolerability and Immunogenicity of the Prime-Boost Regimen of the Investigational 2012/13 Seasonal Influenza DNA Vaccine, VRC-FLUDNA063-00-VP, Followed by the 2012/2013 Seasonal Influenza Trivalent Inactivated Vaccine (TIV) Compared to TIV Prime-TIV Boost in Children and Adolescents Ages 6-17 Years

Sponsored by:
Vaccine Research Center, National Institute of Allergy and Infectious Diseases
National Institutes of Health

I, the Investigator of Record for the indicated Study Site, agree to conduct this study in full accordance with the provisions of this protocol. I agree to maintain all study documentation pertaining to the conduct of this study, including CRFs, source documents, consent forms, laboratory test results, and medication inventory records, for at least 2 years following submission of a Biologics License Application, unless directed otherwise by the VRC. No study records will be destroyed without prior authorization from NIAID. Publication of the results of this study will be governed by the VRC and NIAID policies. Any presentation, abstract, or manuscript will be made available by the investigators to the VRC Leadership Group and to NIAID for review prior to submission.

I have read and understand the information in this protocol and will ensure that all associates, colleagues, and employees assisting in the conduct of the study are informed about the obligations incurred by their contribution to the study.

______________________________	______________________________
Name /Title of Investigator of Record	Study Site Name/Identifier
______________________________	______________________________
Signature of Investigator of Record	Date

ABBREVIATIONS

Abbreviation	Term
ACIP	Advisory Committee on Immunization Practices
AE	adverse event
CBC	complete blood count
CC	Clinical Center
CDC	Center for Disease Control
CMV	cytomegalovirus
CRO	Contract Research Organization
DNA	deoxyribonucleic acid
DSMB	Data and Safety Monitoring Board
EDC	electronic data capture
ELISA	enzyme-linked immunosorbent assay
ELISpot	enzyme-linked immunospot
FDA	Food and Drug Administration
GCP	Good Clinical Practices
HA	hemagglutinin
HAI	hemagglutination inhibition
HIV	human immunodeficiency virus
HLA	human leukocyte antigen
HTLV	human T-cell leukemia virus
IB	Investigator's Brochure
ICS	intracellular cytokine staining
IDES	Internet Data Entry System
IM	intramuscular
IoR	Investigator of Record
IRB	Institutional Review Board
ITT	intent-to-treat
LAIV	live attenuated influenza vaccine
LIMS	Laboratory Information Management System
LTR	long terminal repeat
MCB	master cell bank
MedDRA®	Medical Dictionary for Regulatory Activities
MIV	monovalent inactivated vaccine
NA	neuraminidase
NH	Northern Hemisphere
NIAID	National Institute of Allergy and Infectious Diseases
NIH	National Institutes of Health
NVITAL	NIAID Vaccine Immune T-Cell and Antibody Laboratory
PBMC	peripheral blood mononuclear cells
PBS	phosphate-buffered saline
PSRT	Protocol Safety Review Team
RNA	ribonucleic acid

Abbreviation	Term
RVP	reporter virus particle
SAE	serious adverse event
SH	Southern Hemisphere
S-OIV	Swine-Origin Influenza Virus
TIV	trivalent inactivated vaccine (for seasonal influenza)
TNF	tumor necrosis factor
UNI-CPSC	Universal Influenza Clinical Program Support Center
VAERS	Vaccine Adverse Events Reporting System
VIS	vaccine information statements
VPP	Vaccine Pilot Plant
VRC	Vaccine Research Center
WHO	World Health Organization
WNV	West Nile virus

PRÉCIS

VRC 702: An Open-Label, Dose-Escalation, Phase I Study of the Safety, Tolerability and Immunogenicity of the Prime-Boost Regimen of the Investigational 2012/13 Seasonal Influenza DNA Vaccine, VRC-FLUDNA063-00-VP, Followed by the 2012/2013 Seasonal Influenza Trivalent Inactivated Vaccine (TIV) Compared to TIV Prime-TIV Boost in Children and Adolescents Ages 6-17 Years

Study Design: This is a Phase I, dose escalation study in healthy adolescents and children (6-17 years) to evaluate the safety, tolerability, and immunogenicity of a prime-boost regimen of the 2012/2013 seasonal influenza DNA vaccine (HA DNA) followed by licensed 2012/2013 TIV vaccine. The comparator groups will receive licensed 2012/2013 TIV as prime and boost.

The hypothesis is that the HA DNA prime-TIV boost regimen will be safe for administration to children and adolescents and result in a broader and more durable immune response than is observed in the TIV-TIV group. The primary objective of the study is to evaluate the safety and tolerability of the HA DNA vaccine at two dosages (1 mg and 4 mg) followed by TIV at Week 18 in school-age groups of children 6-11 and adolescents 12-17 years old. Secondary objectives are the HAI response to homologous strains (frequency and magnitude) at 4 weeks after the TIV boost and breadth of neutralizing antibody response to a standardized panel of influenza strains at 4 weeks after the TIV boost. Other exploratory objectives are related to the humoral and cellular immune responses through Study Week 42.

Product: The HA DNA vaccine, VRC-FLUDNA063-00-VP, is developed by VRC, NIAID and composed of 3 closed-circular DNA plasmids that encode for hemagglutinin (HA) from the following 2012-2013 influenza strains:

- A/California/04/2009 (H1)
- A/Victoria/361/2011 (H3)
- B/Wisconsin/1/2010

HA DNA vaccine is supplied at 4 mg/mL in single use vials. Injections are administered intramuscularly (IM) in deltoid muscle using the Biojector® 2000 Needle-Free Injection Management System (Biojector). The 1 mg dosage is administered as 0.25 mL volume and the 4 mg dosage as a 1 mL volume. The TIV injections are administered in accordance with manufacturer specifications using needle and syringe.

Subjects: Target accrual is 70 healthy adolescents and children; 35 who are 12-17 years and 35 are 6-11 years. The protocol includes an allowance for over-enrollment up to 76 subjects.

Study Plan: The groups in this study are defined by age and prime vaccine administered as shown in the Schema. Enrollments will follow a dose escalation plan by age range such that the older age group is the first to receive each dosage of HA DNA vaccine. The decisions for starting each group will be based on a Data and Safety Monitoring Board (DSMB) safety review.

Group numbers 1and 2 indicate the HA DNA prime dosage of 1 mg and 4 mg, respectively. Group 3 indicates a TIV prime-TIV boost comparator group. The study groups are stratified by age; subgroups labeled “A” indicate the older age range (12-17 years) and “B” indicates the younger age range (6-11 years). Accrual of Group 1 is not

associated with randomization into the comparator group. Randomizations into Group 2 and Group 3 for a given age subgroup will occur simultaneously in a 1:1 ratio.

Throughout the study, safety data will include collection of 7-day solicited reactogenicity after each vaccination; collection of all unsolicited adverse events through 28 days after each study vaccination and collection of SAEs, influenza events and new chronic medical conditions through 24 weeks after the boost study injection. Blood draws for assessment of immunogenicity will be baseline, Week 4, Week 18, Week 22 and Week 42.

VRC 702 Schema						
**Study Group**	**Subjects / Group**	**Stratification by age**	**Age (years)**	**Subjects**	**Prime Day 0**	**Boost Week 18±2 wks**
1	10	1 A	12-17	5	HA DNA (1 mg)	TIV (2012/2013)
		1 B	6-11	5	HA DNA (1 mg)	TIV (2012/2013)
2	30	2 A	12-17	15	HA DNA (4 mg)	TIV (2012/2013)
		2 B	6-11	15	HA DNA (4 mg)	TIV (2012/2013)
3	30	3 A	12-17	15	TIV (2012/2013)	TIV (2012/2013)
		3 B	6-11	15	TIV (2012/2013)	TIV (2012/2013)
Target Accrual	70	All HA DNA injections are administered IM by Biojector, TIV administered IM by needle and syringe				
• Begin enrollment of Group 1A<br>• DSMB Review after 5 in Group 1A have 1 week follow-up; if satisfactory, begin Groups 1B and randomizations to Groups 2A and 3A.<br>• DSMB Review after 5 in Group 1B have 1 weeks follow-up; if satisfactory, begin randomizations to Group 2B and 3B						

The DNA prime-TIV boost groups (Group1 and Group 2) will require 6 clinic visits as follows: Day 0 DNA prime, Week 1, Week 4, Week 18 (TIV boost), Week 22, Week 42 and 2 telephone follow-up contacts (at 1-2 days after the prime and 7-10 days after the boost).

The TIV-TIV comparator group (Group 3) may require 7 clinic visits if the 2012/2013 TIV supply is not yet available on day of randomization, as follows: Randomization day, Day 0 TIV prime (to be scheduled when the 2012/2013 TIV is available), Week 1, Week 4, Week 18 (TIV boost), Week 22, Week 42 and 2 telephone follow-up contacts (at 7-10 days after each of the TIV injections). If TIV supplies are available, then randomization and TIV prime injection will both occur on Day 0.

Study Duration: Each subject will complete follow up through Study Week 42 (24 weeks after administration of the TIV boost vaccination). The overall study completion is estimated to take about a year to complete with a projected timeline as follows: staged dose escalation and randomizations in June-August 2012 with TIV boosts for DNA prime groups in September-December 2012. For the TIV prime group, the TIV primes will occur once TIV supplies are available and the interval of 18 weeks between prime and boost will be maintained. Follow-up for durability of immune responses is expected to be completed July 2013.

1 INTRODUCTION

The Dale and Betty Bumpers Vaccine Research Center (VRC), NIAID, NIH (Bethesda, MD) is dedicated to translating the latest knowledge of disease pathogenesis and immunology into new vaccine strategies to provide safe and effective means to prevent and control infectious diseases. The 2009-2010 H1N1 pandemic influenza reinforced awareness that rapidly preparing a vaccine for use during an influenza season and consideration of the different levels of immunity and risks present in different age groups in the population is important for public health [1]. The need for influenza vaccines that are both more immunogenic and able to induce universal immune responses effective against a broad spectrum of influenza strains is well recognized [2]. In this protocol we propose to use a DNA vaccine prime followed by boosting with traditional licensed split product trivalent influenza vaccine (TIV) to induce immune responses against influenza hemagglutinin (HA) in healthy adolescents and children (6-17 years). Investigation of the DNA prime-inactivated boost approach in adults using an H5 DNA vaccine demonstrated that the regimen is safe and that immune response to inactivated vaccine can be augmented by DNA vaccine priming administered as compared with two vaccinations with the inactivated vaccine when the prime-boost interval is 24 weeks, but not when the interval is as little as 4 weeks [3]. Additional data (Ledgerwood, manuscript in preparation) indicates that the improved response is observed when the boost interval is 16 weeks or more.

1.1 INFLUENZA BIOLOGY, NATURAL HISTORY AND VACCINES

Influenza is a negative-strand ribonucleic acid (RNA) virus with a segmented genome that belongs to the family *Orthomyxoviridae*. Of the three genera of influenza circulating in nature (influenza A, B, and C), only the first two are known to cause epidemics [4]. Influenza A viruses have 8 open reading frames that encode 10 viral proteins. They are classified on the basis of the antigenicity of their surface glycoproteins: hemagglutinin (HA) and neuraminidase (NA). Sixteen HA subtypes and nine NA subtypes are known to exist, but only three HA subtypes (H1, H2, and H3) and two NA subtypes (N1 and N2) have caused significant human epidemics [5].

The public health burden of influenza in the world is enormous. Annual influenza epidemics cause about 250,000 to 500,000 deaths worldwide [6]. Circulating viruses change quickly and re-assort with each other creating new viruses. These present an immediate threat to public health and currently require the preparation of new vaccines directed at the changing viral strains that are prevalent annually. Emerging virus strains present the potential of a pandemic when there is little or no pre-existing immunity in the population as observed in worldwide outbreaks of influenza in the last century [7]. A global pandemic caused by the spread of a novel swine-origin influenza virus (S-OIV) in the 2009-2010 influenza season is a recent example of this challenge to public health.

Vaccines are an effective way of preventing influenza infection and transmission in humans. Annually, the World Health Organization (WHO) and the U.S FDA make recommendations on the composition of the seasonal influenza vaccine, with recommendations for the Northern Hemisphere (NH) and for the Southern Hemisphere (SH) considered at different times based on epidemiology data [8]. The annually licensed influenza vaccines consist of 3 components: Influenza A (H1N1), Influenza A (H3N2), and an influenza B virus strain. These vaccines depend upon labor-intensive methods that limit manufacturing capacity and have low immunogenicity to induce type-specific responses. Currently, the vaccine composition requires

an adjustment for emerging antigenically-modified influenza strains and efficacy is limited in vulnerable populations.

For seasonal influenza infection, the rates of serious illness and death are the highest among vulnerable populations such as persons older than 65 years, children younger than 2 years, and persons of any age who have medical conditions that place them at increased risk for complications from influenza [9]. Seasonal influenza causes significantly higher morbidity and mortality in older adults than in younger adults and these adverse outcomes are attributed to an age-related decline in immune function [10].

Seasonal TIVs have demonstrated a clinical efficacy of 70% to 90% in younger adults, ~ 58% in those aged 60 to 69 years, and 30% to 40% in those aged 70 or more years [11]. When the seasonal vaccine and circulating viruses are antigenically similar, seasonal TIV prevents laboratory-confirmed influenza illness among approximately 70%-90% of healthy adults less than 65 years old in randomized controlled trials. Immunization efficacy was 47%-77% in studies conducted during influenza seasons when the vaccine strains were not an exact antigenic match to the majority of circulating influenza strains [9].

In the 2009-2010 H1N1 influenza pandemic, the CDC determined that the seasonal TIV was unlikely to provide protection against a novel pandemic influenza A (H1N1) that was an antigenically distinct influenza virus to which little or no pre-existing immunity was detected in the population [1]. The epidemiologic pattern of this novel swine-origin influenza virus (S-OIV) strain included a high prevalence (60% of infected patients) in the population ≤ 18 years old, who had not been exposed to the influenza strains that circulated in 1950-1970s that had partial antigenic similarity with the novel H1N1 strain [1, 12]. Using stored serum specimens collected in previous vaccine studies, CDC assessed the level of cross-reactive antibodies to the novel influenza A (H1N1) virus in cohorts of children and adults before and after they had been vaccinated with the 2005-2009 influenza seasonal vaccines [1]. The results indicated that 33% of adults older than 60 years and 6-9% of adults aged 18-64 years had some cross-reactive antibody to the novel influenza, and no cross-reactive antibody was found in children. The results also suggested that seasonal influenza vaccines are unlikely to provide protection against novel emerging influenza virus strains. Development of a universal influenza vaccine that will have an improved cross-reactivity and efficacy against novel emerging influenza strains, as well as high potency in the vulnerable high-risk populations is a priority for public health in the United States and worldwide [13].

Several conserved regions that may serve as broad neutralizing epitopes to afford cross-protection against a variety of influenza strains have been identified in the influenza viral protein structures [14-16]. NIAID scientists have been engaged in the investigation of immune responses to influenza and identification of the broadly-neutralizing antibodies that may lead to development of a more universal influenza vaccine [2, 16]. The DNA vaccine prime-inactivated vaccine boost strategy being evaluated in this protocol has shown evidence of improved immune response, including to epitopes conserved between influenza subtypes [3]. The rationale for and prior experience with this strategy are summarized in Sections 1.2 and 1.4 below.

1.2 Rationale for Seasonal Influenza DNA Vaccine Prime-Inactivated Boost

DNA vaccines have the potential to be manufactured rapidly. They are known to induce balanced immune responses that induce both humoral and cellular immunity. The potential and experience to date with influenza DNA vaccines warrants continued investigation. One approach

to improving immunity to influenza would be to prime the population with a DNA vaccine early in the year and to boost with TIV when the influenza season begins in order to provide better immunogenicity, especially to novel influenza strains. This may be a useful strategy for the older adult and pediatric populations for which the TIV vaccine alone has a lower efficacy than in young adults. In previous studies, the VRC has found that antibody responses are higher after the boost when there is a long prime-boost interval, such as 6 months compared to a short interval, such as 1 month [3]. For this reason, one dose DNA priming schedules with a long interval between prime and boost are being evaluated towards the goal of an influenza vaccination strategy with improved immunogenicity.

A DNA vaccine may also elicit CD8 T cell responses to conserved HA epitopes that may afford some cross-protection [17-19]. It has also been suggested that T cell responses are the important correlates of influenza vaccine protection, especially in the elderly populations immunized with the seasonal flu vaccines [20, 21].

1.3 Rationale for Influenza DNA Vaccine Testing in Children

Children have been shown to have an increased burden of influenza infection [22] and to facilitate disease transmission to others in the community [23]. Clinical diagnosis of influenza is difficult in children and although severe disease requiring hospitalization is rare, outpatient clinic visits and days missed (for child and parent) have a significant public health impact.

Vaccination against influenza remains the most effective way of preventing influenza infection in children. The CDC Advisory Committee on Immunization Practices (ACIP) recommends routine annual influenza vaccinations for all persons aged $\geq$ 6 months, with 2 doses of influenza vaccine to be administered at least 4 weeks apart for the first vaccination in children aged 6 months through 8 years. The two types of vaccine preparations are licensed by the FDA for administration in children, TIV preparations for children aged $\geq$ 6 months and preparations of live attenuated influenza vaccine (LAIV) which are indicated for children aged $\geq$ 2 years. Both types of influenza vaccine are confirmed as safe and immunogenic in children. Although ACIP specified no preference for LAIV versus TIV, there are several lines of evidence indicating that live vaccine induces more functional immune response and higher level of protection against influenza infection [24, 25].

The effectiveness of vaccine priming depends on the antigenic similarity of the priming and boosting vaccine, and the children who received one vaccine dose may require two doses the following season if the seasonal vaccine strains have been changed [26]. Also it has been suggested that using a regimen of TIV prime in the spring with TIV boost in the fall allows for earlier immune response development in naïve children in comparison to vaccine administration in the fall alone. This leads to the earlier protection of the higher proportion of children even if the antigens in priming and boosting TIVs are mismatched [27].

The immune system in children is characterized by immature B and T cell responses and this is likely among the factors that result in a greater susceptibility to severe infections and a necessity of repeated vaccinations. Factors that limit the overall efficacy of influenza vaccination include response rates, magnitude of responses, and breadth of responses. As a vulnerable population, children require special protection in vaccine research studies; however, society as a whole and children in particular may benefit the most from these studies if vaccines that improve rate, magnitude and breadth of response are developed as the result.

Various strategies, including the use of adjuvants or DNA vaccines have been suggested as ways of improving vaccine immunogenicity in children [28, 29]. In this study we plan to evaluate the DNA vaccine approach because in adults, studies with an H5 DNA influenza vaccine administered as a prime injection prior to inactivated influenza vaccine boosting has been shown to improve the overall magnitude of antibody responses and in some cases induced hemagglutinin-stem-specific neutralizing antibodies, which should improve the breadth of immune response [3]. This finding indicates potential value for DNA vaccine priming as a public health measure against influenza and warrants further evaluation in expanded age groups, including children and adolescents.

1.4 Previous Human Experience with VRC DNA Vaccines

1.4.1 Plasmid DNA Vaccines Developed by VRC, NIAID, NIH

Investigators at the VRC/NIAID/NIH in Bethesda, MD have evaluated plasmid DNA vaccine strategies targeted to several different infectious diseases since 2001 in preclinical and clinical studies. Cumulatively through 2011, more than 2000 study subjects had been vaccinated with VRC DNA vaccines. Dosages up to 8 mg have been administered, with the majority of injections being at a 4 mg dosage. Data from dose-escalation studies indicate that a 4 mg dosage offers the combination of a good safety profile, greater ease of administration than an 8 mg dosage, and reliable immunogenicity as indicated by laboratory measures of immune response. Preclinical and clinical evaluations to date of several plasmid DNA vaccines support the safety and immunogenicity of VRC DNA vaccines at the 4 mg dosage. The results of human clinical trials with VRC DNA vaccines have been published for HIV DNA vaccines [30-37], an Ebola virus DNA vaccine [38], a SARS DNA vaccine [39], West Nile virus DNA vaccines [40, 41] and H5 DNA vaccine [3]. VRC clinical trials of WNV and SARS vaccines have provided evidence that a DNA vaccine can induce neutralizing antibody as assessed by a reporter virus particle (RVP) neutralizing antibody assay [39-41]. Experience to date indicates that there may be advantages to using a Biojector for DNA vaccine delivery compared to using a standard needle and syringe [42].

1.4.2 Influenza DNA Vaccines Developed by VRC, NIAID, NIH

The VRC, NIAID, NIH has developed several investigational influenza DNA vaccine products. Clinical evaluation of the first influenza DNA product was initiated in 2006 with an avian influenza H5 DNA vaccine. Through a series of clinical trials, influenza DNA vaccines have been tested alone or in prime-boost regimens with the respective inactivated vaccines in the clinical trials as shown in **Table 1-1**. Of these studies, all are complete except VRC 701 which will be ongoing at the same time as VRC 702.

Table 1-1: Experience with VRC Influenza DNA Vaccines in Adult Population

IND Number	Vaccine(s)	Antigen(s)	Protocols
BB-IND 13197	VRC-AVIDNA036-00-VP	HA of A/Indonesia/05/2005 (H5N1)	VRC 304<br>VRC 305
BB-IND 13836	VRC-AVIDNA036-00-VP (with inactivated vaccine boost)	HA of A/Indonesia/05/2005 (H5N1)	VRC 306<br>VRC 310
BB-IND 13939	VRC-FLUDNA047-00-VP (with inactivated vaccine boost)	Trivalent HA matching the 2008-2009 seasonal strains	VRC 307
	VRC-FLUDNA056-00-VP (with inactivated vaccine boost)	Trivalent HA matching the 2009-2010 seasonal strains	VRC 309
	VRC-FLUDNA061-00-VP (with inactivated vaccine boost)	Trivalent HA matching the 2011-2012 seasonal strains	VRC 701
BB-IND 14093	VRC-FLUDNA057-00-VP (with inactivated vaccine boost)	HA of pandemic A/California/04/2009 (H1N1)	VRC 308

H5 DNA vaccine alone (BB-IND 13197) was evaluated in two Phase 1 studies. One study (VRC 304) investigated intramuscular (IM) administration of the vaccine or placebo in three groups of healthy adults 18-60 years of age who received 3 IM injections of 1 mg vaccine, 4 mg vaccine, or placebo, respectively. All injections in VRC 304 were via Biojector. The second study, VRC 305, investigated intradermal (ID) administration of the vaccine by 4 schedules: 0.5 mg ID by needle, 0.5 mg ID by Biojector, 0.5 mg ID x2 in the same arm, and 0.5 mg ID in each arm. Cumulatively, 89 subjects were enrolled. Between the two studies, 204/222 (92%) of the expected DNA vaccinations were administered to 74 vaccine recipients and 42/45 (93%) of expected phosphate buffered saline (PBS) injections were given to 15 placebo recipients.

The H5 DNA vaccine was well tolerated by both the IM and ID routes. Detailed final study reports are available in the BB-IND 13197. In VRC 304, no apparent dose effect on frequency, duration, or severity of reactogenicity was noted. There were two serious adverse events (SAE) in VRC 304: a Grade 3 leukocytosis, attributed to concomitant medications taken for a musculoskeletal injury and a Grade 3 paroxysmal hemicrania in a subject with a history of severe headaches. A neurologist assessed this as unlikely to be related to vaccine. In VRC 305, there were no SAEs.

The H5 DNA vaccine alone was not strongly immunogenic by either the IM or ID routes.

H5 DNA Vaccine in prime-boost regimens (BB-IND 13836) was evaluated in two Phase 1 studies (VRC 306 and VRC 310) in healthy adults ages 18-60 years old. Between these two studies the prime-boost regimens evaluated included two doses of monovalent inactivated vaccine (MIV, Sanofi Pasteur H5N1 A/Indonesia 05/05 avian influenza vaccine) at 4 or 24 week intervals or H5 DNA vaccine followed by MIV at varying intervals from 4 to 24 weeks. Cumulatively, 124 subjects were enrolled. Between the two studies, 114/114 (100%) of the expected H5 DNA injections were administered to 99 subjects randomized to schedules with H5 DNA primes and 147/149 (99%) of the expected H5N1 MIV injections were administered to 122 subjects.

There was no severe local or systemic reactogenicity. Mild local reactogenicity was reported in a

majority of the H5 DNA vaccine recipients (83%) while 2% reported moderate local reactogenicity. The majority of the H5 DNA vaccine recipients reported no systemic reactogenicity; 23% reported mild and 5% reported moderate systemic reactogenicity. No SAE were reported following the H5 DNA vaccinations; there was one SAE reported in the follow-up period through 48 weeks: multiple injuries unrelated to study participation in the period after the H5N1 booster. All adverse events following H5 DNA injection were mild (Grade 1) or moderate (Grade 2) except for one severe (Grade 3) case of gastroenteritis with onset 15 days after H5 DNA vaccination, which was assessed as unrelated to vaccine.

In assessing immunogenicity of H5 DNA vaccine followed by MIV, a single H5 DNA vaccine prime followed by a single MIV boost at short intervals (4-8 weeks) did not significantly improve HAI titers over that achieved with inactivated vaccine alone. However, a single H5 DNA 4 mg vaccination prime significantly improved HAI responses when the interval to the inactivated vaccine boost was 20-24 weeks, as compared with two vaccinations with the inactivated vaccine [3]. Therefore, a single dose of DNA priming may be sufficient to increase the magnitude and breadth of HA-specific antibody responses.

<u>Seasonal HA DNA vaccine studies</u>: Specifically, with regard to seasonal influenza vaccines developed by the VRC/NIAID/NIH, VRC-FLUDNA047-00-VP, VRC-FLUDNA056-00-VP, VRC-FLUDNA061-00-VP (BB-IND 13939 and BB-IND 14093) and VRC-FLUDNA063-00-VP are season-specific 3-plasmid DNA vaccines; while VRC-FLUDNA057-00-VP is a single plasmid H1 DNA based on the 2009 pandemic H1N1 influenza virus. **Table 1-2** shows the composition of these investigational influenza DNA vaccines.

Table 1-2: Composition of VRC Seasonal Influenza DNA Vaccine Products

Product/IND reference	**Plasmids and Expressed Antigens**							
	**H1**		**H3**			**B**		
	VRC-9269	VRC-9328	VRC-9270	VRC-2439	VRC-3027	VRC-9271	VRC-9323	VRC-2722
	A/Brisbane 59/07	A/California 04/09	A/Brisbane 10/07	A/Perth 16/09	A/Victoria 361/11	B Florida 4/06	B Brisbane 60/08	B Wisconsin 1/10
VRC-FLUDNA047-00-VP Seasonal 2008-09/IND 13939	X		X			X		
VRC-FLUDNA056-00-VP Seasonal 2009-10/IND 13939	X		X				X	
VRC-FLUDNA057-00-VP Pandemic H1 2009/IND 14093		X						
VRC-FLUDNA061-00-VP Seasonal 2011-12/IND 13939		X		X			X	
VRC-FLUDNA063-00-VP Seasonal 2012-13/IND TBD		X			X			X

Three clinical trials (VRC 307, VRC 308 and VRC 309) have been completed in adults with seasonal HA DNA vaccines and one ongoing clinical trial (VRC 701) completed the

administration of the blinded HA DNA (or placebo) prime injections in February 2012. VRC 307 (2008-2009 season) and VRC 309 (2009-2010 season) were Phase 1 clinical trials, and VRC 701 (2011-2012 season) is a Phase 1b clinical trial to evaluate trivalent seasonal HA DNA constructs in adults 18-70 years old. VRC 308 was a Phase 1 clinical trial to evaluate a 1-plasmid construct for the 2009 pandemic H1 influenza in adults 18-70 years old. The prime-boost interval of VRC 307 and VRC 309 was 3 or 4 weeks with matched seasonal TIV, while for VRC 308, due to delayed availability of the pandemic H1N1 inactivated vaccine, the boost was added by amendment to be offered to subjects as an option, and the boost intervals varied. The boost interval in VRC 701 is planned to be 36 weeks with a subsequent season's TIV.

In VRC 307 and VRC 309, the DNA constructs were the same except for the encoded influenza B antigen. Cumulatively, a total of 111 subjects were enrolled; 66 were randomized to a single HA DNA prime-TIV boost, 25 to a single PBS prime-TIV boost, and 20 to TIV prime-TIV boost schedules. Between the two studies 65/66 (99%) of the expected HA DNA vaccinations, 25/25 (100%) of the expected PBS injections and 126/131 (96%) of the expected TIV vaccinations were administered. The seasonal HA DNA vaccines were well tolerated. There was no severe local or systemic reactogenicity. Similar to the H5 DNA vaccine, a majority of seasonal HA DNA vaccine recipients (78%) experienced mild local reactogenicity and 2% experienced moderate local reactogenicity. The majority (66%) of HA DNA vaccine recipients reported no systemic reactogenicity, while 31% reported, mild and 3% reported moderate systemic reactogenicity. No SAE were reported in either VRC 307 or VRC 309. All adverse events were mild or moderate except for three Grade 3 events following HA DNA prime injections (urticaria at one day post vaccination, influenza at 11 days post vaccination, and increased alanine aminotransferase [ALT] at 33 days post vaccination); one Grade 3 event following PBS prime injection (neutropenia at 14 days post injection); and one Grade 3 event (gastroenteritis at 24 days post vaccination) following TIV. Based on temporal relationship, urticaria was the only Grade 3 event attributed as possibly related to DNA vaccine.

VRC 308 included 20 subjects on a schedule of three H1 DNA vaccinations at 4 week intervals; 60/60 (100%) of expected H1 DNA vaccines were administered and the vaccine was well tolerated. When the licensed H1N1 inactivated vaccine became available, 18 subjects opted to receive the boost as well. There was no severe local or systemic reactogenicity. The majority (90%) of H1 DNA vaccine recipients reported mild local reactogenicity; one subject (5%) reported moderate local reactogenicity. Over 3 injections with H1 DNA vaccine, 60% reported mild and 15% reported moderate systemic reactogenicity. There were no SAE and all adverse events were mild or moderate in severity.

VRC 701 includes 131 subjects (18-70 years) enrolled in Jan-Feb 2012 who were randomized to receive a blinded injection of 2011/12 HA DNA vaccine or placebo, which will be followed by a boost with the 2012/13 TIV in Fall 2012. Interim data as of February 27, 2012 indicates that the (still blinded) prime injections were well-tolerated. Solicited adverse events (reactogenicity) were collected for 7 days. For local reactogenicity, 41.2% reported none, 55% reported mild and 3.8% reported moderate as the maximum severity. For systemic reactogenicity, 76.3% reported none, 21.4% reported mild and 2.3% reported moderate as the maximum severity. Pain at the injection site was the most frequently reported solicited AE (55% of subjects), with headache reported by 18.2% and all other solicited symptoms each reported by fewer than 10% of subjects. Fever was the least frequently reported symptom and was recorded by 1 subject (0.8%). There have been no SAEs. Unsolicited adverse events have been reported for 34 of 131 (26%) subjects.

All were mild or moderate except for one report of a severe vasovagal reaction, which was not associated with loss of consciousness but was considered grade 3 because the systolic blood pressure was 68. Mild bruising at the injection site was the most frequently reported (6.1%). Mild macular rash was reported by 1 subject at the injection site (0.8%) and at other than injection site by 2 subjects (1.5%) within the first week after injection. No subjects have discontinued the study to date.

Summary of HA DNA safety and immunogenicity: The safety data for the VRC HA DNA vaccine trials to date shows that these vaccines were well tolerated. No severe local or systemic reactogenicity occurred nor were there any serious adverse events related to vaccine. The pattern of local and systemic reactogenicity appears to be generally mild and similar across the studies, independent of the antigens encoded by the HA DNA constructs included in any one vaccine.

Regarding immunogenicity, seasonal HA DNA priming did not significantly improve HAI response compared to TIV alone with the boost administered at a 3-4 week interval. However, data from studies of the H5 DNA vaccine using various H5N1 inactivated vaccine (MIV) boost intervals show that a longer interval is associated with a higher magnitude of immune response.

Based on experience with the H5 DNA vaccine, future development of the trivalent seasonal HA DNA is directed towards evaluation of an inactivated boost at intervals of 16 weeks or longer post-prime as a potential method of reliably inducing a strong immune response to seasonal influenza vaccine.

The immunogenicity data from the 36 week boost interval in the VRC 701 study will not be available by the time the VRC 702 study in children and adolescents is initiated.

1.5 EXPERIENCE WITH DNA VACCINES IN CHILDREN

To our knowledge, this study would be the first Phase I study of a DNA vaccine in healthy children and adolescents in the U.S. There is one published study conducted in Italy of a multiclade, multigene therapeutic HIV DNA vaccine manufactured in Sweden and evaluated at 3.8 mg dose (3 vaccinations IM) in HIV-infected children 6-16 years old on an antiretroviral treatment regimen. The vaccine was reported as well tolerated with self-limited local reactogenicity and no severe systemic reactions [43]. In this Phase II study, twenty children ages 6-16 years were enrolled and randomized to intramuscular injections with HIV DNA vaccine at weeks 0, 4, 12 and the boosting dose planned at week 36, or to control group that was continuing antiretroviral regimen. Safety data collected through three months after the last priming DNA injection at week 12 indicated that the DNA vaccine was safe and well tolerated. Solicited local and systemic reactogenicity was collected in the 7 days after each vaccination, and all reactions were reported as mild and self-limited with resolution within 72 hours. Out of total 86 solicited adverse events, 25 were attributed as likely related to vaccination. Local reactogenicity was mostly manifested as local irritation at the site of vaccination such as itching or erythema with or without swelling while systemic reactogenicity was mostly headache and/or fatigue. No severe systemic adverse events were reported, and no decrease in CD4 T-cell count or virologic failure was observed.

1.6 ASSESSMENT OF IMMUNOGENICITY

In protocol VRC 702, specimens to evaluate immunogenicity will be obtained at screening and at specified timepoints. The primary immunogenicity timepoint is 4 weeks after the boost.

Measurements of antibody, B cell and T cell response will be assessed. Hemagglutinin (HA)-specific antibody as measured by HAI assay is the traditional benchmark measure of immune response to influenza vaccines and will be conducted on stored samples from throughout the study. To evaluate durability of HA-specific immune responses, the response will be assessed at 4 and 24 weeks after the boost. Because study subjects are likely to have pre-existing immune responses to many influenza HA antigens and the effect of vaccination on other humoral and T cell responses is of interest, a variety of exploratory evaluations of immunogenicity may also be performed. HA-specific T cell responses measured by intracellular cytokine staining (ICS) assay and ELISpot, other HA-antibody assays, and assays to evaluate cross-reactivity will be performed at timepoints throughout the study as exploratory evaluations.

The detection of antibody by HAI assay is based on a validated laboratory method. The ICS assay is based upon previously published methods [44] and quantitates the frequency of $CD4^+$ and $CD8^+$ cells that produce interleukin-2, interferon-gamma, or TNF-alpha in response to pools of overlapping peptides representing HA antigens. Specific peptides will also be used to detect T cell responsiveness by an ELISpot assay, modified from a previously published method [45]. Research samples for immunogenicity assays will be processed by the NIAID Vaccine Immune T Cell and Antibody Laboratory (NVITAL) in Gaithersburg, MD, where many of the immunogenicity assays will also be performed. Some immunogenicity assays may be performed by VRC laboratories in Bethesda, MD, approved contract laboratories, or approved research collaborators.

2 STUDY VACCINES

2.1 FORMULATION AND MANUFACTURING OF VRC-FLUDNA063-00-VP

The VRC-FLUDNA063-00-VP Drug Substance consists of three closed-circular plasmid DNA macromolecules, in equal amounts by weight, that express Influenza A HA sequences for strains that meet the criteria for production of the 2012-2013 licensed seasonal influenza vaccine, as follows:

- VRC-9328: HA (Gene Bank Protein Accession # ACQ76318)-Influenza A virus (A/California/04/2009) (H1N1),
- VRC-3027: HA (EpiFlu Accession # EPI353906)-Influenza A virus (A/Victoria/361/2011) (H3N2),
- VRC-2722: HA (NCBI Accession #AET22022)-Influenza B virus (B/Wisconsin/1/2010)

The plasmid CMV/R promoter consists of translational enhancer region of the CMV immediate early region 1 enhancer (CMV-IE) substituted with the 5′-untranslated human T-cell leukemia virus type 1 (HTLV-1) R-U5 region of the human T-cell leukemia virus type 1 HTLV-1 long terminal repeat (LTR), and has been shown to increase expression of the encoded gene in comparison to the CMV promoter [46]. This promoter has been evaluated in preclinical safety studies as well as in many clinical trials.

VRC-FLUDNA063-00-VP is manufactured at the VRC/NIAID/Vaccine Pilot Plant (VPP) using plasmid DNA received from the VRC to produce clinical trial material under current Good Manufacturing Practices (cGMP). The process for manufacturing, filling, and packaging the vaccine is summarized in the Investigator’s Brochure (IB). Briefly, the plasmids used in the master cell banks (MCB) were synthesized using human preferred codons as previously

described [47]. The plasmids were then transferred to the VPP and their sequences confirmed before use. Each plasmid was used to transform the *Escherichia coli* bacterial host strain, DH5α, in order to produce individual MCB. Each MCB was expanded in culture and inoculated into a fermentor for production. Bacterial cell growth was dependent upon the cellular expression of the kanamycin resistance protein encoded by a portion of the plasmid DNA. Following growth of bacterial cells harboring the plasmid, the plasmid DNA was purified from cellular components, concentrated, filtered, and stored until formulation of the study vaccine. The final vaccine product will meet lot release specifications prior to administration.

The study vaccine is manufactured at a 4 mg dose in phosphate buffered saline (PBS). Vials are aseptically filled to a volume of 1.2 mL with 4 mg/mL plasmid.

2.2 Formulation and Manufacturing of Seasonal Trivalent Influenza Vaccine

Study sites will refer to the manufacturing package insert for complete product information. The TIV used for the booster injection will be a licensed Northern Hemisphere (NH) product approved by the U.S. FDA for administration during the 2012-2013 influenza season, composed of three strains: A/California/7/2009 (H1N1)pdm09-like, A/Victoria/361/2011 (H3N2)-like and B/Wisconsin/1/2010-like).

2.3 Pre-Clinical Studies of HA DNA Vaccines

No preclinical pharmacology, toxicology, pharmacokinetic, or metabolism studies were conducted for the seasonal influenza DNA vaccine VRC-FLUDNA063-00-VP. There is extensive clinical experience with licensed seasonal influenza antigens, as well as human clinical trial experience with DNA vaccines constructed using the same plasmid backbone and CMV/R promoter, including other seasonal influenza DNA vaccines.

FDA recommendations permit manufacturers with established seasonal flu vaccines to file supplemental applications each year after changing one or more of the three influenza strains in the vaccine. This reflects the large safety experience with similar influenza vaccine products as well as the severe time constraints on design and manufacture of both investigational and licensed vaccines due to the seasonality of influenza infections. Although VRC is evaluating investigational vaccines based on the DNA platform, the influenza gene inserts mimic the antigens found in the yearly seasonal TIV and our approach has been similar in this regard.

Refer to the Investigator's Brochure for more information about preclinical studies.

3 STUDY OBJECTIVES

3.1 Primary objective:

- To evaluate the safety and tolerability of a single injection of the recombinant DNA vaccine VRC-FLUDNA063-00-VP (administered IM with a Biojector at dosages of 1 mg or 4 mg) followed at week 18 by the 2012/13 seasonal influenza TIV in children 6-11 years and adolescents 12-17 years old as compared to age-matched groups that receive two TIV injections 18 weeks apart.

3.2 Secondary objective:

- To evaluate the frequency of HAI titers that are ≥1:40 or 4-fold greater than baseline at 4 weeks after the TIV boost in each group.

- To evaluate the frequency of strain specific H1, H3 and B neutralizing antibodies that are 4-fold greater than baseline at 4 weeks after the TIV boost in each group.

3.3 EXPLORATORY ANALYSES:

- To evaluate the presence of anti-stem antibodies pre- and post-vaccination at 4 weeks after the seasonal influenza TIV boost in each group.
- To evaluate antibody or T cell responses in the HA DNA vaccine-TIV and TIV-TIV groups at timepoints throughout the study.

4 STUDY DESIGN

This is a Phase I, dose escalation study in healthy adolescents and children (6-17 years). The hypothesis is that the 2012/2013 seasonal influenza DNA vaccine (HA DNA) prime-TIV boost regimen will be safe for administration to children (6-11 years) and adolescents (12-17 years) and result in a broader and more durable immune response than is observed in age-matched comparator TIV-TIV groups. The study schema is shown in **Table 4-1**. The groups in this study are defined by prime vaccine administered and age. Enrollments will follow a dose escalation plan by age range such that the older age group is the first to receive each dosage of HA DNA vaccine. The decisions for starting the next stage of enrollments will be based on a Data and Safety Monitoring Board (DSMB) safety review.

Group numbers 1 and 2 indicate the HA DNA prime dosages of 1 mg and 4 mg, respectively. Group 3 indicates a TIV prime-TIV boost comparator group. Subgroups labeled "A" indicate the older age range (12-17 years) and "B" indicates the younger age range (6-11 years). Enrollments into Group 1 are not associated with randomization into the comparator group. Randomizations into Group 2 and Group 3 for a given age group will occur simultaneously in a 1:1 ratio.

Table 4-1. VRC 702 Study Schema

Study Group	Subjects / Group	Stratification by age	Age (years)	Subjects	Prime Day 0	Boost Week 18±2 wks
1	10	1 A	12-17	5	HA DNA (1 mg)	TIV (2012/2013)
		1 B	6-11	5	HA DNA (1 mg)	TIV (2012/2013)
2	30	2 A	12-17	15	HA DNA (4 mg)	TIV (2012/2013)
		2 B	6-11	15	HA DNA (4 mg)	TIV (2012/2013)
3	30	3 A	12-17	15	TIV (2012/2013)	TIV (2012/2013)
		3 B	6-11	15	TIV (2012/2013)	TIV (2012/2013)
*Target Accrual	70	All HA DNA injections are administered IM by Biojector, TIV administered IM by needle and syringe				

- Begin enrollment of Group 1A
- DSMB Review after 5 in Group 1A have 1 week follow-up; if satisfactory, begin Groups 1B and randomizations to Groups 2A and 3A.
- DSMB Review after 5 in Group 1B have 1 week follow-up; if satisfactory, begin randomizations to Group 2B and 3B

*See **Section 6.4** for the allowance on over-enrollment.

The expected duration of time on study per subject is approximately 42 weeks. Whenever possible, randomization and first study injection will occur on Day 0. However, for the TIV-TIV comparator group, if the 2012/2013 TIV supply is not yet available on day of randomization; then Day 0 visit will be scheduled upon TIV availability. The projected study timeline includes the staged randomizations in June-August 2012 with TIV boosts for DNA prime groups in September-December 2012. For the TIV prime group, the TIV primes will occur once TIV supplies are available and the interval of 18 weeks between prime and boost will be maintained. The protocol includes contingencies for early administration of the 2012/2013 TIV if there is known potential exposure to influenza circulating in the community.

Follow-up for durability of immune responses is expected to be completed in July 2013.

4.1 STUDY POPULATION

Children and adolescents will be enrolled upon completion of informed consent by a parent or legal guardian and assent by the minor child. The screening and education process prior to enrollment should ensure that the legal adult representative of the child and the child, to an age appropriate level, understand the purpose of the study. The specific eligibility requirements for this study will be confirmed prior to enrollment of the subject. The following eligibility criteria will be used:

4.1.1 Inclusion Criteria

A volunteer must meet all of the following criteria:

1. Children/adolescents aged 6 to 17 years inclusive and at least 20 kg in weight.

2. Able to provide proof of identity to the satisfaction of the study clinician completing the enrollment process.

3. Willing to have blood drawn 5 times over 42 weeks, including blood stored for research purposes.

4. In good general health as assessed by medical history, vital signs and targeted physical examination; stable medical conditions that, in the opinion of the investigator, will not compromise the subject's participation in the study are acceptable.

5. Capability of the legal adult representative of the minor to understand and comply with planned study procedures.

6. Capability of the legal adult representative of the minor to provide written informed consent; assent will be obtained from the child/adolescent per requirements of the site IRB.

7. For female adolescent of child-bearing potential (as defined by onset of menses): agrees to avoid becoming pregnant and to use effective method of contraception or practice abstinence for at least 21 day prior to the first study vaccine administration, until at least 4 weeks after the second study vaccination.

8. Within 70 days prior to enrollment, hemoglobin within institutional normal limits,

creatinine ≤ upper limit of normal (ULN) and ALT ≤1.5 X ULN for respective age group.

4.1.2 Exclusion Criteria

A subject will be excluded if one or more of the following conditions apply:

1. History of Guillain-Barré syndrome.
2. Active neoplasm or history of cancer.
3. On-going immunosuppressive therapy or known to be immunosuppressed at the time of enrollment.
4. Immunoglobulin (or similar blood product) therapy within 3 months prior to enrollment.
5. Known to have HIV, hepatitis B or hepatitis C infection.
6. Acute or chronic illness that, in the opinion of the investigator, precludes participation in the study.
7. Developmental delay, neurologic disorder, or seizure disorder requiring ongoing medical management (note: history of febrile seizure is not an exclusion).
8. Acute febrile and/or respiratory illness within one week prior to enrollment.
9. Idiopathic urticaria within the year prior to enrollment.
10. Allergy treatment with antigen injections, unless on maintenance schedule and allergy shots could be staggered with the study vaccinations, within 14 days (2 weeks) prior to enrollment.
11. Asthma that is severe, unstable or required emergent care, urgent care, hospitalization or intubation during the previous two years or that is expected to require the use of oral, intravenous or high dose inhaled corticosteroids.
12. Vaccination of any type within 2 weeks prior to enrollment or receipt of any of the licensed 2012/2013 seasonal influenza vaccines any time prior to enrollment.
13. Participating in or planning to begin participation in another investigational study during the projected time during which the subject would be in this study.
14. Factors related to the legal representative that in the judgment of the investigator may affect the objective decision-making of the legal representative.
15. For a female adolescent of child-bearing potential: breast-feeding, known pregnancy or positive urine or serum pregnancy test on day of study enrollment.
16. Any 6, 7 or 8 year old child enrolling on or after July 16, 2012 who did not receive at least two licensed influenza vaccinations between July 1, 2010 and June 30, 2012.

4.2 STUDY SCHEDULE

The study schedule is presented in the form of a Table in **Appendix III**. After Day 0, deviations from the visit windows in completing study visits are discouraged and will be recorded as protocol deviations, but are permitted, at the discretion of the IoR (or designee) in the interest of obtaining subject safety and immunogenicity evaluations following study vaccinations. Any TIV boost administered outside of the target interval window, in consultation with the IND Sponsor, to ensure that the subject has been vaccinated at least once with the licensed TIV before community exposure to influenza is likely to occur, will not be recorded as protocol deviation. This is a contingency permitted by the protocol as being in the best interests of the subject.

During or following any visit, if there is any concern about the well-being of the subject, the clinical study site will conduct appropriate medical evaluations by history, physical, laboratory or other indicated testing.

4.2.1 Pre-enrollment

Potential study subjects whose parent or guardian verbally agrees to discuss the child's medical history may complete a scripted interview questionnaire by telephone or in person that covers protocol inclusion and exclusion criteria that are based on key self-reported history information to identify potential study volunteers.

4.2.2 Screening Visit(s)

Screening for this study may be completed through a general screening protocol after signing consent to be screened or screening consent may be incorporated into a VRC 702-specific consent/assent form. A screening segment for the study will be included in data collection to provide information on reasons for non-enrollment to begin study vaccinations. Evaluations will be done according to eligibility criteria and clinical assessment at screening. Screening evaluations for specific eligibility criteria must be completed within the time interval specified prior to enrollment (see Appendix III), but may be repeated as needed to confirm eligibility.

As part of the screening process, an Assessment of Understanding (AoU) will be completed by the parent/guardian and incorrect answers will be discussed as part of the consent process; participation by the child/adolescent will be included to assist with the assent process.

Screening records will be kept to document the reason why an individual was screened but not enrolled into the clinical trial.

4.2.3 Enrollment Visit

Prior to study enrollment, the eligibility criteria will be reviewed by a study clinician and informed consent/assent process completed.

For all groups, enrollment is defined as the day of the first study injection. For Groups 1 and 2 (HA DNA-TIV) randomization and enrollment will occur on the same day (Day 0). For Group 3 (TIV-TIV) it is possible that randomization to the group may occur early in Summer 2012 before TIV is available; therefore the day of randomization and day of enrollment may be separate days. The timing of the TIV prime injection for this group will depend upon TIV availability, although it is expected that all TIV primes in Group 3 will be administered primarily in August 2012. Once randomized into a TIV-TIV group, eligibility assessments do not need to be repeated prior to a TIV prime injection even if the screening visit occurred greater than 70 days prior to administration of the TIV because TIV is not an investigational product.

The day of the prime study injection is designated Study Day 0 for all groups. Day 0 evaluations prior to the first injection are the baseline for subsequent safety assessments; however, for any evaluation not performed on Day 0 (e.g., creatinine, ALT, hemoglobin), the baseline will be the screening evaluation.

4.2.4 Administration of Study Injections:

Pregnancy test results for adolescents of reproductive potential must be confirmed as negative prior to the Day 0 and Week 18 study injections.

HA DNA injections will be administered into deltoid muscle in a 0.25 mL volume for 1 mg dose (Group 1) and 1 mL volume for 4 mg dose (Group 2) using a Biojector 2000® needle-free injection system (Biojector; Bioject Medical Technologies Inc., Portland, OR). The Biojector will be used as directed by the manufacturer. The Biojector uses sterile, single-use syringes for administration of volume up to 1 mL. The study agent is delivered under pressure by a compressed CO_2 gas cartridge that is stored inside the Biojector. Neither the material being injected nor injection site skin preparation requires deviation from standard procedures. The CO_2 does not come in contact with the injectate and the syringe design prevents any back splatter or contamination of the device by tissue from the subject.

The first study injections for Group 3 subjects and the second injection in all Groups will be the 2012/13 seasonal influenza TIV. The TIV injections will be administered IM at the standard dosage of 45 mcg in a 0.5 mL volume into deltoid muscle by needle and syringe, according to the manufacturer's directions.

It is recommended, but not required, that study injections be administered into the non-dominant arm. When choosing an arm for the injection, clinicians should consider whether there is an arm injury, local skin problem or significant tattoo that precludes administering the injection or will interfere with evaluating the arm after injection.

Following study injection, subjects will be observed for a minimum of 30 minutes after the DNA vaccine injection, and for a minimum of 15 minutes after the TIV injection. Temperature, blood pressure, pulse and respiratory rate will be taken between 30 and 60 minutes post-injection for HA DNA and 15 to 60 minutes for TIV. The injection site will be inspected for evidence of local reaction. If erythema is present, the largest perpendicular diameters will be measured and recorded. Acute medical care will be provided to subjects for any immediate allergic reactions or other injury resulting from participation in this research study.

7-Day Diary Card and Follow-up: Subjects (through their legal adult representative) will be given a "Diary Card", to use as a memory aid, on which to record temperature and symptoms daily for 7 days following each injection. The site may use the electronic or written (paper) diary card as a source document or clinician notes obtained by telephone interview as the source of reactogenicity information recorded in the study database. The solicited signs and symptoms on the diary card will include the parameters: unusually tired/feeling unwell, muscles aches (other than at injection site), headache, chills, nausea, and pain/tenderness at injection site. Subjects will also record the day's highest measured temperature and measurement of largest diameter for redness and swelling at injection site.

As an exploratory method for assessing pain, while in the clinic at the following two timepoints; 1) 30 ± 15 minutes after each injection and 2) at the Day 7 follow-up visit that occurs after prime injections, the child or adolescent subject will be asked to assess **pain at the injection site** by

choosing a face from the "FACES Pain Rating Scale." As this is exploratory to evaluate the tool for possible future use, the subject will **not** be asked to complete this assessment for post-vaccination Days 1 to 6.

Before dismissal from the clinic, the subject's adult legal representative will be asked to contact the clinic if the subject has any concerning signs or symptoms. A clinic visit will be scheduled, if indicated, for the following: rash, urticaria, fever of 38.5˚C (Grade 2) or higher lasting greater than 24 hours, or significant impairment in the activities of daily living (ADL). Additionally, other clinical concerns may prompt a study visit based on the judgment of a study clinician.

4.2.5 Blood Sample Collection

Blood Drawing Limitations: At intervals throughout the study, blood will be drawn for safety and immunologic assays. Blood will be drawn from the arm veins of subjects by standard phlebotomy procedures.

In this pediatric study, no more than 3 mL/kg may be drawn over any 8-week period.

Only children who cooperate willingly will continue to participate. Children who resist participating in the blood draw will not be restrained for the purposes of obtaining samples. Topical anesthetics may be used to facilitate venipuncture following the site-specific procedures. Pregnancy is not a contraindication to blood drawing and pregnancy testing is not a requirement before collection of blood samples. However, as pregnancy increases the potential for anemia, no additional research samples will be collected after it is learned that an enrolled female subject is pregnant.

4.2.6 Concomitant Medications and Procedures

Current concomitant medications are recorded in the study database at enrollment. Concomitant medications will be updated in the study database if there is an occurrence of an adverse event that requires expedited reporting. Treatment for influenza with antiviral drugs will be recorded on an Influenza Endpoint Case Report Form. Sites should work with study subjects with regard to the timing of FDA-approved vaccines such that, when possible, they are scheduled to be administered at least 14 days before or at least 28 days after a study vaccination. Receipt of a licensed vaccine at any time is not a protocol violation. Vaccinations received during study participation will be recorded in the study database. Otherwise, a record of concomitant medication changes throughout the study will not be recorded in the study database.

4.2.7 Protocol Considerations Related to the Licensed TIV

Contingencies Related to Potential Exposure to Influenza in the Community: If exposure to communities with circulating influenza is likely to occur before a subject in the HA DNA prime group has received TIV (such as in case of an earlier than expected local outbreak of influenza or if the subject will be traveling to the SH), then in consultation with the IND Sponsor, the vaccination with TIV will be offered ahead of the targeted schedule timepoint in order to ensure that the subject has been vaccinated at least once with the licensed TIV before community exposure to influenza is likely to occur. If the concern is such that the schedule adjustment will apply to multiple subjects, then guidance will be provided by the IND sponsor regarding the prime-boost interval in the TIV-TIV group as well so that the intervals in the relevant "comparator groups" remain comparable to the intervals in HA DNA prime groups.

Contingencies Related to Children 6-8 Years of Age in VRC 702: In June 2012, after the VRC 702 clinical trial was in progress, the Advisory Committee on Immunization Practices (ACIP) met and recommended the following for the 2012/2013 influenza season for children ages 6 months to 8 years: Those who have received a total of at least two seasonal influenza vaccinations since July 2010 need to receive only one dose of the 2012/2013 influenza vaccine. Providers may consider giving one dose in 2012/2013 if a child in this age range had received influenza vaccine prior to July 2010. Otherwise, children in this age range should receive two influenza vaccinations at least 4 weeks apart in the 2012/2013 season. In the VRC 702 study, the above recommendation affects only the 6, 7, and 8 year olds. An eligibility criterion for this age range was added in consideration of this recommendation under the Version 2.0 protocol to avoid the need to administer a second TIV injection to a 6-8 y.o. child who is not randomized to a TIV prime-TIV boost group. Any 6-8 y.o. in Group 1B already enrolled under protocol Version 1.0, who does not meet the amended eligibility criterion may be offered a second TIV vaccination at 4 weeks after the planned TIV vaccination at the discretion of the site PI if deemed in the best interest of the child.

4.3 Criteria for Dose Escalation

The NIAID Intramural Data and Safety Monitoring Board (DSMB) will conduct two interim safety data review and assess the data as showing no significant safety concerns before specific subsequent groups may begin enrollment.

Interim safety data review #1: This review is for Group 1A (12-17 years) and will be conducted when at least 5 subjects have completed 1 week follow-up after HA DNA prime vaccination.

If the safety review for Group 1A (age 12-17 years, 1 mg HA DNA dose) is satisfactory, enrollment in Group 1B (age 6-11 years, 1 mg dose) and randomizations in Groups 2A and 3A (both age 12-17 years), 4 mg HA DNA dose and TIV, respectively, will begin.

Interim safety data review #2: This review is for Group 1B (6-11 years) and will be conducted when at least 5 subjects have completed 1 week follow-up after HA DNA prime vaccination.

If the safety review for Group 1B (age 6-11 years, 1 mg dose) is satisfactory, randomizations into Groups 2B and 3B (age 6-11 years), 4 mg HA DNA dose and TIV, respectively, will begin.

Subsequently, the DSMB will conduct reviews at their regularly scheduled meetings until study completion.

The site IRBs will be provided with documentation of the DSMB reviews.

4.4 Criteria for Discontinuing Subject Participation

In general, subjects who receive the Day 0 study injection will continue to be followed according to the schedule of safety and immunogenicity evaluations. The second study injection is a licensed seasonal influenza TIV. Therefore, this study does not require discontinuation of the injection schedule unless circumstances have arisen that constitute a contraindication to administering the TIV injection. Pregnancy is not a contraindication to a single TIV injection, but research blood draws will be discontinued as indicated in **Appendix III** and a second TIV

injection for those on a TIV-TIV schedule should not be administered. A subject may be discontinued from protocol participation for the following reasons:

1. Subject or guardian decides to discontinue participation.
2. Subject develops a medical condition that is a contraindication to continuing study participation.
3. The Sponsor or regulatory authority stops the protocol.
4. The Site Investigator of Record (IoR) assesses that it is not in the best interest of the subject to continue participation in the study or that the subject's compliance with the study is not sufficient.

4.5 Pausing the Study

4.5.1 Criteria for Pausing the Study

The Protocol Chair and UNI-CPSC Medical Monitor will closely monitor and analyze study data as they become available. The VRC Medical Officer will provide an independent review of adverse events on a regular basis. The administration of study injections and new enrollments will be paused according to the following criteria:

- One (or more) subject experiences serious adverse event (SAE) of any grade that is possibly, probably, or definitely related to HA DNA vaccine.
- Two (or more) subjects experience an SAE regardless of relationship to study vaccine.
- Two (or more) subjects experience the same Grade 3 adverse event assessed as possibly, probably or definitely related to the HA DNA vaccine. Investigator attributions of relationship to vaccination are not collected for the 7-day solicited reactogenicity parameters, however, any Grade 3 reactogenicity reported in the 7 days after vaccination will automatically be assumed to have some possible relationship to the vaccination and will count towards a pause.

4.5.2 Plan for Review of Pauses and Resuming Rules

The study injections and enrollments would resume only if review of the adverse events that caused the pause resulted in a recommendation to permit further study injections and study enrollments. The reviews to make this decision will occur as follows:

Pauses for Grade 4 or 5 events: The IND Sponsor (VRC) will notify the FDA and obtain concurrence on the decision to resume or discontinue the study vaccinations.

Pauses for Grade 3 events (or SAEs that are not Grade 4 or 5): The IND Sponsor (VRC) and UNI-CPSC Medical Monitor, in consultation with the site IoR, will conduct the review and make the decision to resume or close the study for the Grade 3 events that meet the criteria for pausing the study. As part of the pause review, the reviewers will also advise on whether the study needs to be paused again for any subsequent events of the same type.

Changes in study status will be communicated to the study sites promptly by the UNI-CPSC. The site IRBs will be promptly informed with safety data reports and changes in study status in

accordance with institutional policy.

5 SAFETY AND ADVERSE EVENTS

5.1 Adverse Events

5.1.1 Adverse Event (AE) Definition

An AE is any untoward or unfavorable medical occurrence in a human subject, including any abnormal sign (e.g., abnormal physical exam or laboratory finding), symptom, or disease temporally associated with the use of study treatment, whether or not considered related to the study treatment.

5.1.2 Adverse Event Reporting in the Study Database

Each adverse event will be graded according to the table for grading severity of adverse events (see **Appendix IV**).

Solicited adverse events will be recorded in the study database separately with data collection for 7 days after both the first vaccination and the TIV boost injection without the collection of attribution assessments. All unsolicited AEs will be recorded in the study database through 28 days after each study injection. At other time periods during study participation, only SAEs (as detailed in **Section 5.2**), new chronic medical conditions, and influenza or influenza-like illness will be recorded through the last study visit. However, cases of influenza or influenza-like illness will be recorded on an influenza endpoints form rather than on an adverse events form.

Any adverse events associated with TIV that meet the criteria for reporting under the Vaccine Adverse Events Reporting System (VAERS) system, are the responsibility of the IoR to report in accordance with the guidance on reportable events available at the website http://vaers.hhs.gov/professionals/index). Refer to the brochure with description and guidance to the VAERS system, which is co-sponsored by the Centers for Disease Control and Prevention (CDC) and the Food and Drug Administration (FDA): http://vaers.hhs.gov/resources/VAERS_Brochure.pdf.

5.2 Serious Adverse Events

5.2.1 Serious Adverse Event Definition

The term “Serious Adverse Event” (SAE) is defined in the 21 CFR 312.32 in terms of the following outcomes: Death, a life-threatening adverse event, inpatient hospitalization or prolongation of existing hospitalization, a persistent or significant incapacity or substantial disruption of the ability to conduct normal life functions, or a congenital anomaly/birth defect. Important medical events that may not result in death, be life-threatening, or require hospitalization may be considered serious when, based upon appropriate medical judgment, they may jeopardize the patient or subject and may require medical or surgical intervention to prevent one of the outcomes listed in this definition. Examples of such medical events include allergic bronchospasm requiring intensive treatment in an emergency room or at home, blood dyscrasias or convulsions that do not result in inpatient hospitalization, or the development of drug dependency or drug abuse.”

An SAE will be considered "life-threatening" if, in the view of either the investigator or sponsor, its occurrence places the patient or subject at immediate risk of death. It does not include an

adverse event or suspected adverse reaction that, had it occurred in a more severe form, might have caused death.

5.2.2 Reporting Serious Adverse Events to the IND Sponsor

Adverse events that meet Serious Adverse Event (SAE) Reporting Requirements must be reported and submitted by the clinical site on an expedited basis to the IND sponsor, VRC/NIAID/NIH, according to sponsor guidelines as follows:

- death
- life-threatening
- results in persistent or significant disability/incapacity
- requires unplanned inpatient hospitalization or prolongation of existing hospitalization
- a congenital anomaly/birth defect in the offspring of a study subject
- an important medical event that may jeopardize the patient or may require intervention to prevent one of the other outcomes listed above.

In addition, any event, regardless of severity, which in the judgment of a site investigator represents a serious adverse event, may be reported on an expedited basis.

A site investigator will communicate an initial SAE report within 24 hours of site awareness of occurrence to VRC (IND Sponsor) through the communication methods provided by the Data Coordinating Center, EMMES Corporation (Rockville, MD).

Any SAE entered into the study database will generate automatic email notification to the UNI-CPSC Medical Monitor and VRC Medical Officer. This or a written report by the study site sent to the attention of the UNI-CPSC Medical Monitor (Email: uniflu@emmes.com or Fax: 301-576-3558) must be submitted within 3 working days in order for the sponsor to comply with regulations mandating sponsor notification of specified SAEs to the FDA within 7 calendar days.

The investigator must submit additional information as it becomes available.

5.2.3 IND Sponsor Reporting to the FDA

It is the responsibility of the IND Sponsor to make the determination of which SAEs are “serious and unexpected suspected adverse reactions” (SUSARs) as defined in 21 CFR 312.32.

- *Suspected adverse reaction* means any adverse event for which there is a reasonable possibility that the drug caused the adverse event.
- *Unexpected Adverse Event* means an AE that is not listed in the Investigator’s Brochure or is not listed at the specificity or severity that has been observed.

All SUSARs, as determined by the IND Sponsor, will be reported to FDA as IND Safety Reports and IND Safety Reports will be provided to all participating Investigators by the UNI-CPSC.

The IND Sponsor will also submit an IND Annual Report of the progress of the investigation to the FDA as defined in 21 CFR 312.33.

5.3 REPORTING TO SITE IRBS

Each site IoR is responsible for reporting adverse events to the site IRB in accordance with their IRB's requirements for expedited reporting and continuing review reporting.

Site-specific data reports will be made available to facilitate this continuing review reporting. If there is an IND Safety Report, these will be provided to all sites with instruction as to whether or not any actions need to be taken, such as amendment of consent. Investigators must maintain documentation of compliance with actions required for IND safety reports.

5.4 DATA AND SAFETY MONITORING BOARD

The Protocol Safety Review Team (PSRT) (see **Section 8.8**), will have the primary responsibility for the real-time oversight of safety data, SAE reviews and study pause reviews. The IND Sponsor will notify the DSMB of any SAEs assessed as possibly, probably or definitely related to the HA DNA vaccine and of any study pauses within 1 business day of awareness of the event.

The NIAID Intramural DSMB will review cumulative study data to advise on dose escalation and twice per year on regular schedule to evaluate safety, study conduct, and scientific validity and integrity of the trial. As part of this responsibility, DSMB members must be satisfied that the timeliness, completeness, and accuracy of the data submitted to them for review are sufficient for evaluation of the safety and welfare of study subjects. The DSMB will also assess the performance of overall study operations and any other relevant issues, as necessary. Following each review, the DSMB will provide its recommendations to the study sponsor, including whether the study should continue without change, be modified, or be terminated.

6 STATISTICAL CONSIDERATIONS AND SAMPLE ANALYSIS

6.1 OVERVIEW

This study is a multi-center trial to assess the safety and tolerability of a prime-boost schedule that includes the investigational recombinant DNA vaccine VRC-FLUDNA063-00-VP (administered IM with a Biojector at dosages of 1 mg and 4 mg), followed at week 18 by the licensed 2012/13 seasonal influenza TIV as compared to a control group that receives two injections of the 2012/13 seasonal influenza TIV 18 weeks apart. A preliminary assessment of immunogenicity will also be performed.

6.2 OBJECTIVES

The primary objective relates to safety of the HA DNA vaccine. The secondary objectives concern HAI and neutralizing antibody response at 4 weeks after the TIV boost (Study Week 22). The exploratory objectives concern HAI and neutralizing antibody response at other study time points, presence of anti-stem antibodies, and cellular immune response.

6.3 ENDPOINTS

6.3.1 Primary Endpoints: Safety

Assessment of product safety will include clinical observation and monitoring of hematological and chemical parameters. Safety will be closely monitored after injection and evaluated by clinical visits through Study Week 42.The following safety endpoints will be assessed for study groups:

- Occurrence of solicited local reactogenicity signs and symptoms for 7 days following each vaccination
- Occurrence of solicited systemic reactogenicity signs and symptoms for 7 days following each vaccination
- Mean change from baseline for safety laboratory measures at 4 weeks after administration of the prime injection
- Incidence of adverse events of all severities through 28 days after the prime injection and through 28 days after TIV boost
- Incidence of serious adverse events and new chronic medical conditions through last study visit
- Incidence of influenza or influenza-like illness events through last study visit

6.3.2 Secondary Endpoints: Immune Responses

- Proportion of subjects with either a baseline HAI titer < 1:10 and a Week 22 HAI titer ≥ 1:40 or a baseline HAI titer ≥ 1:10 and a minimum four-fold rise from baseline in HAI titer at Week 22.
- Proportion of subjects with a four-fold or greater rise from baseline in specific H1, H3 and B neutralizing antibodies at Week 22.

6.3.3 Exploratory Endpoints: Immune Responses

- Proportion of subjects with either a baseline HAI titer < 1:10 and a post vaccination HAI titer ≥ 1:40 or a baseline HAI titer ≥ 1:10 and a minimum four-fold rise from baseline in post-vaccination HAI titer at Weeks 4, 18, and 42
- Proportion of subjects with a four-fold or greater rise from baseline in specific H1, H3 and B neutralizing antibodies at Weeks 4, 18, and 42
- Proportion of subjects with anti-stem antibodies at Week 22
- Proportion of subjects with positive HA-specific T-cell responses (as measured by ICS assay and ELISpot assay) at Week 22.

6.4 SAMPLE SIZE AND ACCRUAL

The study design is to enroll a total 70 healthy children and adolescents 6-17 years of age in three study groups; 10 subjects will receive 1 mg DNA influenza vaccine prime dose; 30 subjects will receive 4 mg HA DNA influenza vaccine prime dose; 30 subjects will receive TIV prime dose, and all subjects will receive TIV boost 18 weeks following the prime dose. Accrual will be stratified by age group (6-11 or 12-17) with an equal number of subjects enrolled in each age group. For Groups 2 and 3, subjects will be randomized with equal allocation to receive either HA DNA influenza vaccine prime or TIV prime. As shown in Table 4.1, Groups 1 and 2 indicate receipt of HA DNA prime dosage of 1 mg, 4 mg, respectively; Group 3 indicates the group receiving TIV prime; the A designation indicates the older age range 12-17 years and the B designation indicates the younger age range 6-11 years.

The study will be conducted as a dose escalation trial to access the safety, tolerability and immunogenicity of two dosage levels (1 mg and 4 mg) of the trivalent HA DNA influenza vaccine, VRC-FLUDNA063-00-VP, administered to 6-17 year old healthy children and adolescent volunteers and followed by TIV injection 18 weeks later. Accrual will follow a staged accrual plan by age described in Table 4.1 such that the older age group is the first to receive each dosage of vaccine. The decision to stop study accrual for each group will be made by the VRC Protocol Chair.

Accrual may occur rapidly at more than one site. The EMMES Corporation will carefully monitor study accrual and notify all sites and the VRC Protocol Chair when the completion of accrual is near in order to end recruitment of that age group and to plan how to fairly accommodate, to the degree possible, eligible volunteers that have already been recruited. Enrollment of 1 subject in each age group over target accrual is permitted for the 1 mg HA DNA prime schedule and enrollment of 2 subjects per age group over target accrual is permitted in groups randomized to the 4 mg HA DNA prime or TIV prime schedules. Therefore, a total of 76 subjects is permitted to accommodate screened volunteers and may also compensate for potential loss to follow up over the time course of the study. The study plan does not include provision for replacing subjects with incomplete vaccination or visit schedules. If a subject is assigned to a vaccine schedule but withdraws before receiving the prime vaccine, this subject does not count toward the target accrual; an additional subject of the same age group may be enrolled if the minimum target accrual for the age group has not yet been met. Additional enrollments are not done for discontinuations after the prime injection is administered.

6.4.1 Power Calculations for Evaluation of Safety

The goal of the safety evaluation for this study is to identify safety concerns associated with injections of the investigational vaccine. Primary power calculations for safety are expressed in terms of the ability to detect safety or reactogenicity events within each vaccination schedule (n=10 for 1 mg study group; n=30 for 4 mg study group). Power calculations for comparing safety rates between the Groups 2 and 3 are similar to the calculations for immunogenicity shown in Section 6.4.3.

The ability of the study to identify safety events will be expressed in terms of the probability of observing a certain number of events. Useful values are the minimum true event rate such that the probability of observing at least one event is at least 90% and the maximum true event rate such that the probability of not observing any event is at least 90%. In Group 1, there is over 90% chance to observe at least one event if the true rate is at least 0.21; In Groups 2 or 3 there is over 90% chance to observe at least 1 event if the true rate is at least 0.074. Probabilities of observing no events or more than 1 event within each study group are presented in **Table 6-1** for a range of possible true event rates. These calculations provide a more complete picture of the sensitivity of this study design to identify potential safety concerns with the vaccine.

Table 6-1: Probability of Observing Events in Each Study Group

True Event Rate	Study Group 1 (n=10)		Study Groups 2 or 3 (n=30)	
	Pr (0 events)	Pr (>1 event)	Pr (0 events)	Pr (>1 event)
0.005	0.95	0.00	0.86	0.01
0.01	0.90	0.00	0.74	0.04
0.03	0.74	0.03	0.40	0.23
0.05	0.60	0.09	0.21	0.45
0.10	0.35	0.26	0.04	0.82
0.20	0.11	0.62	0.00	0.99

Table 6-2 gives the upper and lower bounds for 95% exact (Clopper-Pearson) binomial confidence intervals of the true event rates for a range of observed event rates in each study group. If none of the 10 vaccinees in Group 1 experience the event of interest, the 95% exact 2-sided upper confidence bound for the event rate is 0.308. If none of the 30 vaccinees in Group 2 or in Group 3 experiences the event of interest, the 95% exact 2-sided upper confidence bound for the event rate is 0.116.

Table 6-2: 95% Confidence Intervals for the True Event Rate for A Range of Observed Rates in each Study Group

	Study Group 1 (n = 10)		Study Groups 2 or 3 (n=30)	
Observed Rate	Lower bound	Upper bound	Lower bound	Upper bound
0.00	0.000	0.308	0.000	0.116
0.10	0.003	0.445	0.021	0.265
0.20	0.025	0.556	0.077	0.386
0.30	0.067	0.652	0.147	0.494
0.40	0.122	0.738	0.227	0.594
0.50	0.187	0.813	0.313	0.687
0.60	0.262	0.878	0.406	0.773
0.70	0.348	0.933	0.506	0.853
0.80	0.444	0.975	0.614	0.923
0.90	0.555	0.997	0.735	0.979
1.00	0.692	1.000	0.884	1.000

6.4.2 Power Calculations for Evaluation of Immune Responses

Table 6-1 gives the probabilities of observing no subjects with immune response or at least 2 subjects with immune responses over a range of underlying response rates. For example, if the true response rate at a particular time point is 0.10, then in Group 2 there is a probability of 0.96 to observe at least one response and a probability of 0.82 to observe at least two responses among the 30 vaccinees.

Table 6-2 is also applicable to the immunogenic response rates, and gives the exact 95% confidence intervals over the range of true response rates for each study group. For example, if

we observe 6 responders among the 30 vaccinees in Group 2, the observed rate is 0.2 with 95% exact binomial confidence interval of 0.777 to 0.386.

6.4.3 Power Calculations for Immunogenicity Comparisons

The secondary objectives are to compare the rates of immune response, in terms of positive HA-specific antibody response or strain specific neutralizing antibody, between Group 2 who receive a 4 mg dose of investigational DNA vaccine VRC-FLUDNA061-00-VP followed by the licensed TIV influenza vaccine and Group 3 who receive two doses of licensed TIV.

Table 6-3 gives the power of the likelihood ratio test to compare the two regimens over a range of possible response rates. **Table 6-4** presents the minimum difference in response rates that can be detected with 80% power and 30 subjects in each study group.

Table 6-3: Power (%) to Detect Difference in Response Rates between Two Study Groups using a Likelihood Ratio Test

	Group 2 DNA-TIV (n=30)					
	Response Rate	0.5	0.6	0.7	0.8	0.9
Group 3 TIV-TIV (n=30)	0.4	12	35	67	91	99
	0.5	-	12	36	71	96
	0.6	12	-	14	42	82
	0.7	36	14	-	16	54
	0.8	71	42	16	-	21

Table 6-4: Minimum Detectable Difference in Response Rates between Study Groups using a Likelihood Ratio Test with 80% Power and Type I Error of 5%

Response Rate		
Group 3 TIV-TIV (n = 30)	Group 2 DNA-TIV (n = 30)	Difference
0.4	0.75	0.35
0.5	0.83	0.33
0.6	0.89	0.29
0.7	0.95	0.25
0.8	0.99	0.19

The exploratory objectives are to compare the proportion of subjects with positive anti-stem antibody or T cell under either the vaccination regimen of the investigational DNA vaccine followed by the licensed TIV or the TIV-TIV regimen, within each age subgroup. The power calculations presented in Tables 6-3 and 6-4 are applicable to these comparisons.

6.5 Statistical Analysis

All subjects who receive at least one vaccination will be included in the safety analysis. All statistical analyses will be performed using Statistical Analysis System (SAS) or R statistical software.

No formal multiple comparison adjustments will be employed for safety endpoints or secondary endpoints.

6.5.1 Analysis Variables

The analysis variables consist of baseline characteristics, safety events, reactogenicity, influenza infections or influenza-like illnesses, and immunogenicity variables for primary and secondary objective analyses.

6.5.2 Baseline Characteristics

Baseline characteristics including demographics, medical history, concomitant medications and laboratory measurements will be summarized using descriptive statistics.

6.5.3 Safety Analysis

Reactogenicity: The number and percentage of subjects experiencing each sign or symptom and type (local or systemic) of reactogenicity will be tabulated by severity. For a given sign or symptom or type, each subject's reactogenicity will be counted once under the maximum severity for all assessments.

Adverse Events: Adverse Events (AEs) will be coded into Medical Dictionary for Regulatory Activities (MedDRA) preferred terms. The number and percentage of subjects experiencing each specific adverse event will be tabulated by severity and relationship to treatment. For the calculations in these tables, each subject's adverse event will be counted once under the maximum severity or strongest recorded causal relationship to treatment.

A complete listing of AEs for each subject will provide details including severity, relationship to treatment, onset, duration and outcome.

Safety Laboratory Values: Safety laboratory values will be summarized as the mean change from baseline (day of first vaccination) along with 95% confidence interval at each timepoint measured in the study. Boxplots of safety laboratory values will be generated for baseline values and for values measured during the course of the study. Each boxplot will show the 1st quartile, the median, and the 3rd quartile, with values smaller than the 1st quartile or larger than the 3rd quartiles plotted as outliers. If appropriate, horizontal lines representing boundaries for abnormal values will be plotted.

6.5.4 Analysis of Immune Responses

Blood draws for assessment of immunogenicity occur at timepoints shown in Appendix III. The statistical analysis of the secondary and exploratory immunogenicity endpoints will employ the intent-to-treat principle, i.e., all data from subjects who received at least one vaccine will be used and analyzed according to the randomized vaccination regimen.

In the final analysis of immunogenicity, a per-protocol analysis may also be performed in which the following would apply:

- if there are cases of a subject receiving a regimen different from the assignment, they will be analyzed as-treated.
- if there are subjects who receive an additional TIV to meet the ACIP recommendation (as described in section 4.2.7), any immune results collected after the additional TIV is received will be excluded from analysis.

If assay data are qualitative (i.e., positive or negative) then analyses will be performed by tabulating the frequency of positive response for each assay at each time point that an assessment is performed. Binomial response rates will be presented with their corresponding exact 95% confidence interval estimates. Fisher's exact tests will be used to compare any two vaccine groups to each other. Missing responses will be assumed to be missing at random, i.e., conditional on the observed data the missingness is independent of the unobserved responses, and no imputations will be performed.

Graphical descriptions of the longitudinal immune responses will also be given.

Some immunologic assays have underlying continuous or count-type readout that is often dichotomized into responder/nonresponder categories. For these assays, graphical and tabular summaries of the underlying distributions will be made. These summaries may be performed on transformed data (e.g., log transformation) to better satisfy assumptions of symmetry and homoscedasticity.

6.5.5 When Analyses May Be Performed

Independent Safety Reviews: The protocol safety review team will review safety data routinely throughout the study. The study will utilize both electronic database features and reviews by designated safety review personnel to identify in a timely manner if any of the safety pause rules of the study are met. The NIAID Intramural DSMB will provide an independent safety review for starting enrollment of the younger age group and dose escalation and at scheduled intervals to coincide with their biannual meeting schedule.

Immunogenicity Review: The analysis of immunogenicity may be performed when the HA HAI assays at 4 weeks after the TIV boost injections have been completed for the study, which may occur prior to completion of all study follow-up. Reports providing results by schedule will be provided to VRC for the purpose of informing decisions related to future trials in a timely manner. The results should in no way influence the conduct of the VRC 702 trial in terms of early termination or later safety or immunogenicity endpoint assessments. Analyses of other immunogenicity assays may also be performed when samples from 4 weeks after TIV boost injections are completed.

6.5.6 Randomization of Treatment Assignments

Randomizations will be done online using the enrollment module of The EMMES Corporations Internet Data Entry System (IDES). The randomization code will be prepared by statisticians at The EMMES Corporation.

Back-up manual randomization procedures and instructions will be provided for use in the event that the site temporarily loses access to the Internet or the online enrollment system is unavailable to a study site.

7 PHARMACY AND VACCINE ADMINISTRATION PROCEDURES

The study groups and vaccination schedules are shown in **Table 4.1**. Refer to **Section 2** for information about manufacturing of study agents.

7.1 STUDY AGENTS

The investigational study agent supplies may only be dispensed by the site pharmacy after the protocol is IRB-approved at the study site. This study includes one investigational vaccine and one non-investigational seasonal TIV vaccine as follows:

- VRC-FLUDNA063-00-VP 4 mg/mL (HA DNA vaccine)
- 2012-2013 Seasonal Influenza TIV

The TIV vaccine will be from the 2012-2013 season and will be from a commercially available vaccine prepared for the Northern Hemisphere (NH).

7.2 STUDY AGENT PRESENTATION AND STORAGE

7.2.1 Study Agent Labels

At the time of delivery of the study agent to the pharmacy, the labels for study agent VRC-FLUDNA063-00-VP (HA DNA vaccine) will have specific product information (e.g., part number, lot number, fill volume, storage temperature) included on the product vial labels. The labels will contain an Investigational Use Statement ("Caution: New Drug – Limited by Federal Law to Investigational Use") and manufacturer information.

The 2012-2013 Seasonal Influenza TIV will be a commercial, licensed vaccine in its original manufacturer's packaging.

7.2.2 Study Agent Storage

Temperature excursions that are outside of the normal allowance for the storage device in which each type of product is kept will be reported to the study sponsor via the study coordinating center (The EMMES Corporation). The excursion must be evaluated and investigated and action must be taken to restore and maintain the desired temperature limits. The site IoR is ultimately responsible for notification of the sponsor, but may delegate this responsibility to a pharmacist. Pending the outcome of the investigation, the site will be informed if continued clinical use of the product is acceptable.

VRC-FLUDNA063-00-VP: Upon release by VRC/NIAID/NIH, the HA DNA vaccine vials will be shipped within the recommended temperature range using appropriate shipping configurations, to the study pharmacist, and will be stored until use at -45°C to -10°C in a qualified, continuously monitored, temperature-controlled freezer.

2012-2013 TIV: The seasonal influenza TIV will be stored according to the label instructions and released by the pharmacist to the designated clinical staff for administration to study subjects.

7.3 PREPARATION OF STUDY AGENT FOR INJECTION

This section describes how the site pharmacist or designee will prepare the HA DNA vaccine and TIV injections. Clinician instructions on how to select an arm and administer the injection are in **Section 4.2.2**.

7.3.1 Preparation of VRC-FLUDNA063-00-VP

The HA DNA vaccine is supplied as a 2 mL glass vial containing a clear colorless isotonic sterile solution. Each vial contains 20% over the amount to be injected in cGMP grade phosphate-buffered saline. Vials are intended for single use only, and thus, do not contain a preservative. They should not be refrozen after thawing. Each vial (4 mg/mL) contains a volume of 1.2 mL (4.8 mg).

Refer to the group assignment for the study subject. For subjects to whom the HA DNA vaccine is to be administered, remove a vial from the freezer. Allow the vial to equilibrate to room temperature (15 to 30º C). Swirl the contents gently. Using aseptic technique, withdraw 0.25 mL or 1 mL of the DNA vaccine from the vial into the Biojector syringe for 1 mg or 4 mg dose respectively, remove air bubbles and cap the syringe. Label the syringe prior to delivery to the clinic with the subject identifier and the date and time allowance for administration.

The injection must be administered within 8 hours after the vial is removed from the freezer.

7.3.2 Preparation of 2012-2013 TIV Injection

Each 0.5 mL dose of the seasonal influenza TIV contains 45 mcg total comprised of 15 mcg of influenza virus hemagglutinin of 3 different strains as approved by the FDA for the 2012-13 influenza season. Each injection is administered in the clinic in accordance with the package insert instructions.

7.4 STUDY AGENT ACCOUNTABILITY

7.4.1 Documentation

Each study site will be responsible for maintaining an accurate record of the codes, inventory, and an accountability record of the investigational vaccine supplies for this study at their site. Electronic documentation as well as paper copies may be used.

7.4.2 Disposition

The empty vials and the unused portion of a vial will be discarded in a biohazard containment bag that will be incinerated or autoclaved. Any unopened vials that remain at the end of the study will be discarded at the discretion of the VRC in accordance with policies that apply to investigational agents. Partially used vials or expired prepared doses cannot be administered to other subjects nor used for *in vitro* experimental studies and will be discarded as indicated above.

8 HUMAN SUBJECTS PROTECTION

8.1 INSTITUTIONAL REVIEW BOARD

A copy of the protocol, proposed informed consent and assent forms, other written subject information, and any proposed advertising material will be submitted to the site IRB for review and approval.

The Site IoR will submit and, where necessary, obtain approval from the IRB for subsequent protocol amendments and changes to the informed consent document. The Site IoR is responsible for ensuring proper IRB notification of deviations from the protocol or serious adverse events occurring at the site and other adverse event reports received from the VRC, NIAID, in accordance with the protocol and local IRB policies. The IoR will be responsible for

obtaining annual IRB approval/renewal throughout the duration of the protocol. Documentation of the IRB approval and FWA number will be provided for the Sponsor's records.

8.2 Subject Recruitment and Enrollment

Subjects for this study will be recruited by the sites in accordance with their site IRB standard for recruitment practices.

8.2.1 Participation of Children

This study meets the Department of Health and Human Services regulations (45 CFR 46, Subpart D, 401-409) for inclusion and protections for children who participate in research. Healthy children can participate in research studies considered as "not greater than minimal risk" and can participate in research with greater than minimal risk only when it presents the prospect of direct benefit to the individual child or is likely to yield generalizable knowledge about the child's disorder or condition and meets the criteria in 45 CFR 46, Subpart D.

Participation in this study will not provide any benefits directly to individual children; however, this study is likely to yield generalizable knowledge that is considered necessary to development of an influenza vaccine regimen that would provide durable immune responses and be protective against multiple influenza strains. In accordance to the 45 CFR 46, Subpart D, research involving greater than minimal risk and no prospect of direct benefit to the individual subjects should be scientifically sound and significant, and have the following conditions met to be approved:

(a) The risk represents a minor increase over minimal risk;
(b) The intervention or procedure presents experiences to subjects that are reasonably commensurate with those inherent in their actual or expected medical, dental, psychological, social, or educational situations;
(c) The intervention or procedure is likely to yield generalizable knowledge about the subjects' disorder or condition which is of vital importance for the understanding or amelioration of the subjects' disorder or condition; and
(d) Adequate provisions are made for soliciting assent of the children and permission of their parents or guardians, as set forth in 45 CFR 46.408

The conditions listed above have been considered and incorporated in this protocol for minimization of risk to children who may participate. The study schedule has been adjusted for time constrains such that children will receive their seasonal influenza vaccination on the recommended timeline.

8.3 Informed Consent

The provided template informed consent and assent documents (**Appendix I)** will be used to guide development of the site-specific consent forms. Only an IRB-approved consent/assent form will be used to consent subjects for participation in the study. The changes in the informed consent/assent template by the site should be approved with the VRC Program Officer before submission to respective IRB. The written informed consent/assent documents will be prepared in the language(s) of the potential subject population. Before a subject's participation in the protocol, it is the investigator's responsibility to ensure that written informed consent/assent is obtained from the subject after adequate explanation of the aims, methods, anticipated benefits, and potential hazards of the protocol.

The acquisition of informed consent/assent should be documented in the subject's records, as

required by 45 CFR 46.117, and the informed consent form should be signed and personally dated by the subject and by the person who conducted the informed consent discussion. An original signed informed consent/assent form should be retained by the site and a signed copy of the consent/assent form should be provided to the subject.

8.3.1 Minor Assent

For enrollment of children and adolescents in the protocol, an assent from the minor and consent for study participation from parent or guardian, as consistent with site IRB and institutional requirements, will be obtained. Study participation is not recommended for children who are excessively anxious about needles and blood drawing, and objection to participation from the child will be considered binding.

Depending on age and comprehension level of the minor as determined by the site IoR or designee, information will be verbally communicated to the child and/or a written assent document will be used. If assent is obtained verbally, this should be documented in a manner consistent with institutional and IRB requirements.

8.4 SUBJECT CONFIDENTIALITY

The investigators at each site must ensure that the subject's anonymity is maintained. Subjects will not be identified in any reports of this study. All records will be kept confidential to the extent provided by federal, state and local law. Medical records will be made available for review when required by authorized agencies and regulatory authorities only under the guidelines set by the Federal Privacy Act. Direct access includes examining, analyzing, verifying, and reproducing any records and reports that are important to the evaluation of the study. The above named representatives will review study-related records without violating the confidentiality of the subjects. Stored study research samples will be labeled by a code (such as a number) that only the site clinical study team can link to the subject. The requirement to maintain subject confidentiality and inform subjects about review of study-related records is included in the study informed consent documents.

8.5 RISKS AND BENEFITS

There is no known benefit to the subject for participating in this protocol, but children and adolescents may benefit from the knowledge gained from this research. The DNA vaccine alone is not expected to provide protection from influenza.

8.5.1 Risks of Blood Collections:

The blood collection procedures are common in routine medical practice. The risks of blood sample collection are minimal and consist of mild discomfort at the sample collection site. The procedure may cause mild pain, bruising, fainting, and, rarely, infection at the site where the blood is taken.

8.5.2 Risks of the DNA Vaccine:

This is the first study in healthy children of any type of DNA vaccine. The risks noted are based on risks from the earlier HA DNA vaccine studies of similar vaccines conducted in adults, as well as risks of vaccines in general and results of previous studies with other investigational DNA vaccines.

Subjects may exhibit general signs and symptoms associated with administration of a vaccine injection including fever, chills, rash, aches and pains, nausea, headache, dizziness and fatigue. These side effects, when they occur, are generally short term, mild to moderate severity, and usually do not require treatment.

In previous VRC DNA vaccine studies, placebo and vaccine recipients were noted to have occasional asymptomatic and self-limited changes in the laboratory tests routinely followed during the clinical trial. Urticaria has been reported as an infrequent adverse event possibly related to DNA vaccines.

Investigational DNA vaccines administered via Biojector have been associated with mild, superficial skin lesions (0.5-1.0 cm diameter) at the vaccination site. In these cases, a small scab formed within 1-2 weeks after immunization and came off after a few days. The skin healed without treatment within a few weeks. One skin biopsy was obtained on Day 6 post vaccination. It showed subcutaneous and dermal perivascular lymphocytic inflammation. There were rare eosinophils and rare giant cells noted, and the infiltrate was composed entirely of CD3 positive cells. It included both $CD4^+$ and $CD8^+$ T cells. The process appears to be primarily a subcutaneous inflammatory response to vaccination with cutaneous manifestations.

There may be other unknown side effects.

8.5.3 Risks of the Seasonal TIV Vaccine:

Occasionally, recipients of seasonal influenza TIV may develop influenza-like reactions such as fever, body aches, headache, malaise, myalgia and/or nausea. These reactions are usually greatest within the first 24 hours after vaccination and last for 1 to 2 days. Some subjects may develop reactions at the site of vaccination (redness, swelling, pain, or tenderness). Analgesics (e.g., ibuprofen and acetaminophen) and rest will generally relieve or moderate these symptoms. These reactions should go away in 1 to 4 days and not require additional treatment.

Acute and potentially life-threatening allergic reactions are also possible. Since the vaccine may contain limited quantity of egg protein, this protein can induce immediate hypersensitivity reactions among person who have severe allergy. Allergic reactions include hives, angioedema, allergic asthma, and systemic anaphylaxis.

During the swine influenza vaccine campaign of 1976, about 1 per 100,000 vaccine recipients developed a paralytic illness called Guillain-Barré Syndrome. This has not been seen consistently with other influenza vaccines. Most persons who develop Guillain-Barré Syndrome recover completely.

There may be other unknown side effects.

8.5.4 Other Risks:

The effect of the investigational DNA vaccine on a fetus or nursing baby is unknown; sexually active female adolescents of reproductive potential will be required to agree to use birth control for sexual intercourse beginning 21 days prior to enrollment and continue through 4 weeks after the last study injection. Adolescents who are pregnant or nursing will be excluded from enrollment into the study.

The licensed seasonal influenza TIV is approved for administration during pregnancy and may be offered to subjects who become pregnant that have not yet received the 2012/13 season TIV. However, because this is a research study, adolescents of reproductive potential will be asked to

notify the site immediately upon learning of a pregnancy during this study and will be tested for pregnancy prior to administration of TIV. As pregnancy increases the potential for anemia, no additional research samples will be collected after an enrolled female subject reports pregnancy, but all safety follow-up visits will be completed if possible. The subject will be contacted to ask about the outcome of a pregnancy that begins during the study.

It is possible that the standard medical tests performed as part of this research protocol will result in new diagnoses. Depending upon the medical findings and consequences of being provided with the new medical information about health status, the study subject/legal guardian may view this aspect of study participation as either a risk or a benefit. Any such information will be shared and discussed with the subject/legal guardian and, if requested by the subject/legal guardian, will be forwarded to the subject's primary health care provider for further workup and management.

8.5.5 Benefits:

Study subjects may have no direct benefit from participation in this study. This protocol is not designed to provide treatment for any condition. The TIV vaccine may provide protection against influenza. The DNA priming vaccine may allow for an improved response to the TIV.

8.6 Plan for Use and Storage of Biological Samples

To be eligible for this protocol, subjects must be willing to allow stored specimens to be used in the future for studying infectious diseases, immune function, vaccine responses and other medical conditions. If tests performed at a study site show evidence of any acute or chronic condition, subjects will be informed of the results and advised to seek appropriate medical care for the condition. In general, testing performed at a research laboratory is not for diagnostic purposes and results will not be available to the study site or study subject.

Intended Use of the Samples/Specimens/Data:

Samples, specimens and data collected under this protocol may be used to study infectious diseases such as influenza, immune function and vaccine responses.

How Samples, Specimens and Data from Sample Use Will Be Stored:

All of the stored study research samples will be labeled by a code (such as a number) that only the study site can link to the subject. Samples will be stored in secure facilities with controlled access at the sites, a central repository maintained by NIH or at central laboratories associated with the study. Samples collected for research may be transferred for testing to the approved collaborators. Data will be kept secure. Only approved investigators or their designees will have access to samples and data. The NIAID Vaccine Immune T-Cell and Antibody Laboratory (NVITAL) in Gaithersburg, MD, under the direction of the VRC, NIAID, NIH (Bethesda, MD) and research labs at or contracted to the VRC or The EMMES Corporation will be involved in conducting assays with stored samples.

In the future, other investigators (both at NIH and outside) may wish to study these samples and/or data. Regulatory approval through the proper human subjects protection agency will be sought prior to any sharing of samples that constitutes human subject research. The research use of stored, unlinked or unidentified samples may be exempt from the need for IRB review and approval. When appropriate, exemption may be obtained through the proper regulatory procedures.

8.7 Compensation

Compensation for study visits and procedures will be provided to offset the time and inconvenience of participation. Subjects will be compensated in accordance with site-specific IRB approval.

8.8 SAFETY MONITORING

8.8.1 Protocol Safety Review Team

Each site IoR (or designee) is responsible for ensuring daily review of the site's clinical safety data as it becomes available. The Protocol Safety Review Team (PSRT) includes the Protocol Chair, IND Sponsor Medical Officer, the UNI-CPSC Medical Monitor and each site IoR or designee. The PSRT will review the summary study safety data reports weekly until 4 weeks after all subjects have completed the HA DNA vaccine study injection in order to be certain that the investigational vaccine has an acceptable safety profile. The PSRT will be notified and convened to review any study pauses. The PSRT will be notified and convened to review a perceived need to begin all TIV boosts in HA DNA prime groups earlier than the target interval due to likely exposure to influenza cases in the community. The Protocol Chair, IND Sponsor Medical Officer, the UNI-CPSC Medical Monitor will continue to monitor the cumulative study safety data reports on at least a monthly basis through completion of the last study visit.

8.8.2 DSMB

As described in Section 5.4, the DSMB will review safety data for dose escalation, SAEs assessed as related to the HA DNA vaccine, any study pauses, and twice per year at their regularly scheduled meetings.

9 ADMINISTRATION AND LEGAL OBLIGATIONS

9.1 PROTOCOL INITIATION, AMENDMENTS AND TERMINATION

Each site must receive IRB approval and approval of The EMMES Corporation before initiating the study at the site. All amendments will also be submitted to the site IRBs for approval. The VRC, NIAID, NIH reserves the right to terminate the study. Each IoR will notify the respective site IRB of study termination in writing and provide documentation to The EMMES Corporation.

9.2 STUDY DOCUMENTATION AND STUDY RECORDS RETENTION

The site IoR will maintain a list of appropriately qualified persons to whom trial duties have been delegated. The site IoR is responsible for ensuring that staff maintains a comprehensive and centralized filing system of all study-related (essential) documentation, suitable for inspection at any time by representatives from the VRC, IRB, The EMMES Corporation and/or applicable regulatory authorities. Elements include but are not limited to:

- Subject files containing completed informed consent forms and supporting copies of source documentation
- Study files containing the protocol with all amendments and copies of all correspondence with the IRB

In addition, all original source documentation must be maintained and readily available.

The EMMES Corporation is responsible for ensuring that records and documents pertaining to the conduct of this study, including CRFs, source documents, consent forms, laboratory test results, and medication inventory records, are retained by the investigator for at least 2 years

following submission of a Biologics License Application or until VRC, NIAID authorizes transfer or destruction of study records. No study records will be destroyed without prior authorization from NIAID.

9.3 Data Collection and Protocol Monitoring

9.3.1 Data Capture Methods

Clinical research data will be collected and recorded by the study sites in a timely fashion in a secure electronic web-based clinical data management system (CDMS) provided by The EMMES Corporation as defined by the contract. Immunological testing on collected, coded blood samples may be performed in batches at central laboratories. Extracted data without subject identifiers will be sent to the statisticians for statistical analysis as needed. The final study database and statistical evaluations will be transferred to the VRC, NIAID at the study completion.

9.3.2 Source Documents and Access to Source Data/Documents

Each participating site will maintain appropriate medical and research records for this trial, in compliance with ICH-GCP, regulatory and institutional requirements for the protection of confidentiality of subjects. As part of participating in the NIAID-sponsored study, each site will permit authorized representatives of the VRC, NIAID, and regulatory agencies to examine (and when required by applicable law, to copy) clinical records for the purposes of quality assurance reviews, audits and evaluation of the study safety and progress.

Source data are all information, original records of clinical findings, observations, or other activities in a clinical trial necessary for the reconstruction and evaluation of the trial. Examples of these original documents and data records include, but are not limited to, medical records, laboratory reports, pharmacy records and other research records maintained for the clinical trial.

9.3.3 Protocol Monitoring

The study data integrity and compliance with the protocol will be assured by the monitoring of the study documentation and study conduct at the sites by The EMMES Corporation. Routine data monitoring and protocol compliance will be performed by the site investigators and study coordinator on an ongoing basis. The study clinical monitoring plan and the data quality monitoring plan will be developed and followed by The EMMES Corporation in consultation with the VRC Program Officer.

9.4 Language

All written information and other material to be used by subjects and investigative staff must use vocabulary and language that are readily understood.

9.5 Policy Regarding Research-Related Injuries

The study site will provide immediate medical care for any injury resulting from participation in this research. In general, the VRC, the NIH, or the Federal Government will not provide long-term medical care or financial compensation for research-related injuries.

10 REFERENCES

1. CDC, *Serum cross-reactive antibody response to a novel influenza A (H1N1) virus after vaccination with seasonal influenza vaccine.* MMWR - Morbidity & Mortality Weekly Report, 2009. **58**(19): p. 521-4.
2. Wei, C.J., et al., *Induction of broadly neutralizing H1N1 influenza antibodies by vaccination.* Science, 2010. **329**(5995): p. 1060-4.
3. Ledgerwood, J.E., et al., *DNA priming and influenza vaccine immunogenicity: two phase 1 open label randomised clinical trials.* Lancet Infect Dis, 2011. **11**(12): p. 916-924.
4. Subbarao, K., B.R. Murphy, and A.S. Fauci, *Development of effective vaccines against pandemic influenza.* Immunity, 2006. **24**(1): p. 5-9.
5. Luke, C. and K. Subbarao, *Vaccines for pandemic influenza.* Emerging Infectious Diseases, 2006. **12**(1): p. 66-72.
6. WHO, *Influenza (Seasonal) Fact Sheet*, 2009: Geneva, Switzerland.
7. Kilbourne, E., *Influenza pandemics of the 20th century.* Emerg Infect Dis [serial on the Internet]. 2006. **available from http://www.cdc.gov/ncidod/EID/vol12no01/05-1254.htm**.
8. WHO. *Recommendations for influenza vaccines*. Available from: http://www.who.int/csr/disease/influenza/vaccinerecommendations/en/index.html.
9. Fiore, A.E., et al., *Prevention and control of seasonal influenza with vaccines: recommendations of the Advisory Committee on Immunization Practices (ACIP), 2009.* MMWR Recomm Rep, 2009. **58**(RR-8): p. 1-52.
10. Targonski, P.V., R.M. Jacobson, and G.A. Poland, *Immunosenescence: role and measurement in influenza vaccine response among the elderly.* Vaccine, 2007. **25**(16): p. 3066-9.
11. Harper, S.A., et al., *Prevention and control of influenza: recommendations of the Advisory Committee on Immunization Practices (ACIP).* MMWR Recomm Rep, 2004. **53**(RR-6): p. 1-40.
12. Novel Swine-Origin Influenza, A.V.I.T., *Emergence of a Novel Swine-Origin Influenza A (H1N1) Virus in Humans.* N Engl J Med, 2009. **360**(25): p. 2605-2615.
13. Du, L., Y. Zhou, and S. Jiang, *Research and development of universal influenza vaccines.* Microbes and Infection, 2010. **12**(4): p. 280-286.
14. Alexander, J., et al., *Identification of broad binding class I HLA supertype epitopes to provide universal coverage of influenza A virus.* Human Immunology, 2010. **71**(5): p. 468-474.
15. Wang, T.T., et al., *Broadly Protective Monoclonal Antibodies against H3 Influenza Viruses following Sequential Immunization with Different Hemagglutinins.* PLoS Pathog, 2010. **6**(2): p. e1000796.
16. Wei, C.-J., et al., *Cross-Neutralization of 1918 and 2009 Influenza Viruses: Role of Glycans in Viral Evolution and Vaccine Design.* Science Translational Medicine, 2010. **2**(24): p. 24ra21.
17. Thomas, P.G., et al., *Cell-mediated protection in influenza infection.* Emerging Infectious Diseases, 2006. **12**(1): p. 48-54.
18. Doherty, P.C., *Toward a broadly protective influenza vaccine.* The Journal of Clinical Investigation, 2008. **118**(10): p. 3273-3275.
19. Jameson, J., J. Cruz, and F.A. Ennis, *Human Cytotoxic T-Lymphocyte Repertoire to Influenza A Viruses.* The Journal of Virology, 1998. **72**(11): p. 8682-8689.

20. McElhaney, J.E. and J.P. Dutz, *Better influenza vaccines for older people: what will it take?* J Infect Dis, 2008. **198**(5): p. 632-4.
21. McElhaney, J.E., et al., *T Cell Responses Are Better Correlates of Vaccine Protection in the Elderly.* J Immunol, 2006. **176**(10): p. 6333-6339.
22. Poehling, K.A., et al., *The underrecognized burden of influenza in young children.* N Engl J Med, 2006. **355**(1): p. 31-40.
23. Weycker, D., et al., *Population-wide benefits of routine vaccination of children against influenza.* Vaccine, 2005. **23**(10): p. 1284-93.
24. Hoft, D.F., et al., *Live and Inactivated Influenza Vaccines Induce Similar Humoral Responses, but Only Live Vaccines Induce Diverse T-Cell Responses in Young Children.* Journal of Infectious Diseases, 2011. **204**(6): p. 845-853.
25. Luce, B.R., et al., *Cost-effectiveness of live attenuated influenza vaccine versus inactivated influenza vaccine among children aged 24-59 months in the United States.* Vaccine, 2008. **26**(23): p. 2841-8.
26. CDC, *Prevention and control of influenza with vaccines: recommendations of the Advisory Committee on Immunization Practices (ACIP), 2011.* MMWR Morb Mortal Wkly Rep, 2011. **60**(33): p. 1128-32.
27. Walter, E.B., et al., *Influenza Vaccine Immunogenicity in 6- to 23-Month-Old Children: Are Identical Antigens Necessary for Priming?* Pediatrics, 2006. **118**(3): p. e570-e578.
28. Vesikari, T., et al., *Oil-in-water emulsion adjuvant with influenza vaccine in young children.* N Engl J Med, 2011. **365**(15): p. 1406-16.
29. Siegrist, C.A., *Potential advantages and risks of nucleic acid vaccines for infant immunization.* Vaccine, 1997. **15**(8): p. 798-800.
30. Tavel, J.A., et al., *Safety and immunogenicity of a Gag-Pol candidate HIV-1 DNA vaccine administered by a needle-free device in HIV-1-seronegative subjects.* J Acquir Immune Defic Syndr, 2007. **44**(5): p. 601-5.
31. Graham, B.S., et al., *Phase 1 safety and immunogenicity evaluation of a multiclade HIV-1 DNA candidate vaccine.* J Infect Dis, 2006. **194**(12): p. 1650-1660.
32. Catanzaro, A.T., et al., *Phase I clinical evaluation of a six-plasmid multiclade HIV-1 DNA candidate vaccine.* Vaccine, 2007. **25**(20): p. 4085-92.
33. Koup, R.A., et al., *Priming Immunization with DNA Augments Immunogenicity of Recombinant Adenoviral Vectors for Both HIV-1 Specific Antibody and T-Cell Responses.* PLoS One, 2010. **5**(2): p. e9015.
34. Kibuuka, H., et al., *A phase 1/2 study of a multiclade HIV-1 DNA plasmid prime and recombinant adenovirus serotype 5 boost vaccine in HIV-Uninfected East Africans (RV 172).* J Infect Dis, 2010. **201**(4): p. 600-7.
35. Jaoko, W., et al., *Safety and immunogenicity study of Multiclade HIV-1 adenoviral vector vaccine alone or as boost following a multiclade HIV-1 DNA vaccine in Africa.* PLoS One, 2010. **5**(9): p. e12873.
36. Churchyard, G.J., et al., *A Phase IIA Randomized Clinical Trial of a Multiclade HIV-1 DNA Prime Followed by a Multiclade rAd5 HIV-1 Vaccine Boost in Healthy Adults (HVTN204).* PLoS One, 2011. **6**(8): p. e21225.
37. Koblin, B.A., et al., *Safety and immunogenicity of an HIV adenoviral vector boost after DNA plasmid vaccine prime by route of administration: a randomized clinical trial.* PLoS One, 2011. **6**(9): p. e24517.

38. Martin, J.E., et al., *A DNA vaccine for Ebola virus is safe and immunogenic in a phase I clinical trial.* Clin Vaccine Immunol, 2006. **13**(11): p. 1267-77.
39. Martin, J.E., et al., *A SARS DNA vaccine induces neutralizing antibody and cellular immune responses in healthy adults in a Phase I clinical trial.* Vaccine, 2008. **26**(50): p. 6338-43.
40. Martin, J.E., et al., *A West Nile virus DNA vaccine induces neutralizing antibody in healthy adults during a phase 1 clinical trial.* J Infect Dis, 2007. **196**(12): p. 1732-40.
41. Ledgerwood, J.E., et al., *A West Nile virus DNA vaccine utilizing a modified promoter induces neutralizing antibody in younger and older healthy adults in a phase I clinical trial.* J Infect Dis, 2011. **203**(10): p. 1396-404.
42. Graham, B.S., et al. *Evaluation of candidate DNA HIV-1 vaccine delivery by Biojector or needle and syringe in healthy adults (VRC 008).* in *AIDS Vaccine 2007*. 2007. Seattle, WA.
43. Palma, P., et al., *The PEDVAC trial: Preliminary data from the first therapeutic DNA vaccination in HIV-infected children.* Vaccine, 2011.
44. Betts, M.R., J.P. Casazza, and R.A. Koup, *Monitoring HIV-specific CD8+ T cell responses by intracellular cytokine production.* Immunol Lett, 2001. **79**(1-2): p. 117-25.
45. Helms, T., et al., *Direct visualization of cytokine-producing recall antigen-specific CD4 memory T cells in healthy individuals and HIV patients.* J Immunol, 2000. **164**(7): p. 3723-32.
46. Barouch, D.H., et al., *A human T-cell leukemia virus type 1 regulatory element enhances the immunogenicity of human immunodeficiency virus type 1 DNA vaccines in mice and nonhuman primates.* J Virol, 2005. **79**(14): p. 8828-34.
47. Yang, Z.Y., et al., *A DNA vaccine induces SARS coronavirus neutralization and protective immunity in mice.* Nature, 2004. **428**(6982): p. 561-4.

Appendix I: Informed Consent Form for Parent/Guardian and Minor Assent Templates

The sample informed consent and assent forms are provided to guide development of site-specific forms. Only IRB-approved consent/assent forms will be used during conduct of the study.

Template Informed Consent Form

STUDY TITLE: VRC 702: An Open-Label, Dose-Escalation, Phase I Study of the Safety, Tolerability and Immunogenicity of the Prime-Boost Regimen of the Investigational 2012/13 Seasonal Influenza DNA Vaccine, VRC-FLUDNA063-00-VP, Followed by the 2012/2013 Seasonal Influenza Trivalent Inactivated Vaccine (TIV) Compared to TIV Prime-TIV Boost in Children and Adolescents Ages 6-17 Years

This informed consent applies to Parents or Legal Guardian

Name of participant: ___________________________________ Age: ___________

The following is given to you to tell you about this research study. Please read this form with care and ask any questions you may have. Your questions will be answered. You will be given a copy of this consent form.

1. What is the purpose of this study?

We are asking you to allow your child to take part in this study because he or she is in the age range of 6 years through 17 years of age.

This research study compares the regular influenza (flu) vaccine, "TIV," with a new experimental vaccine. The experimental vaccine is a "DNA" vaccine. The DNA vaccine is not approved by the Food and Drug Administration (FDA). The TIV vaccine is approved by the FDA for use in the 2012/2013 influenza season.

DNA is used by the body as instructions (a code) for making protein. After the DNA vaccine is injected, the code is used by your child's body to make three flu proteins. The regular TIV flu vaccine has proteins from the flu virus in it. Your child can't get a flu infection from the vaccines.

This study will see if the DNA vaccine is safe and if there are any side effects. This research will also compare if getting one shot of the DNA vaccine and then one shot of the TIV vaccine about 18 weeks later protects your child better than getting two shots of the regular TIV vaccine 18 weeks apart. The DNA vaccine alone is not expected to provide protection from influenza.

Researchers plan to enroll 70 children and/or adolescents at several sites in the United States. We plan to enroll about [insert number] volunteers here at [insert site]. The study is sponsored by the National Institutes of Health (NIH).

2. What will happen and how long will your child be in the research study?

To take part in the study, your child must be in good general health, must be able to tolerate vaccine shots and blood drawing. You and your child must be willing for your child's blood samples to be used in medical research. This research will be about flu, vaccines and the body's responses to infection and vaccines.

Your child may be eligible to be in this study if he or she:

- is 6 years through 17 years old

- has completed the screening process
- has a physical exam and blood test results that meet eligibility
- agrees not to be in another research study of a study product or has research blood drawn for another study while in this study
- you provide written consent by reviewing and signing this consent document
- your child between the age of [**enter age range that applies**] signs an assent document

Screening

The screening process consists of the following:

- Your child will have a physical exam and blood tests to check general health status.
- If your child is a female and has had her first period, she will be asked about her health related to becoming pregnant and birth control use. She will receive a pregnancy test.
- Your child may provide a blood sample to store for research
- We will ask you and your child about your child's general health and flu history. We will ask about any medicines your child is taking and recent vaccinations.
- We will review the screening results with you and tell you if the results may show that your child is not eligible to join the study.

If your child is eligible, he or she will take part in the study for about 10 months (42 weeks).

Study Procedures

At each visit, your child will be checked for any health changes or problems. You will be asked how your child is feeling and if you have given him/her any medications. If your child is a female and has had her first period, she will be asked about her health related to becoming pregnant and birth control use. She will receive a pregnancy test prior to receiving each vaccine.

Three groups of children and adolescents will be enrolled in the study. Group 1 will be enrolled first. After that, Group 2 and Group 3 will be enrolled by random assignment (by chance, like flipping a coin).

- Group 1 will include 10 participants who receive 1 mg of the DNA vaccine and a TIV shot 18 weeks later.

  First, 5 adolescents (12-17 years old) will receive 1 mg of the DNA vaccine. If well tolerated by the adolescents, then 5 children (6-11 years old) will receive 1 mg of the DNA vaccine.

- Group 2 will include 30 participants who receive 4 mg of the DNA vaccine and a TIV shot 18 weeks later.
- Group 3 will include 30 participants who receive a TIV shot followed by another TIV shot 18 weeks later.

Group 2 and Group 3 will each have15 adolescents (12-17 years old) and 15 children (6-11 years old). First, adolescents 12-17 years of age will begin to be randomly assigned to these groups. If the 4 mg dose of the DNA vaccine is well tolerated by the first 5 adolescents, then children (6-11 years old) will begin to be randomly assigned to Group 2 and Group 3.

Each participant will complete follow-up for about 10 months after receiving the first vaccine shot.

Each flu season's TIV vaccine is usually available in August. If your child is randomized to Group 3 and the 2012/2013 seasonal TIV is not yet available, the first vaccine visit will be scheduled once TIV become available.

The DNA vaccine injections will be given using a needleless system called the Biojector 2000®. This device delivers the vaccine through the skin and into muscle without the use of a needle. It uses the pressure of carbon dioxide (CO_2) instead of a needle to inject the vaccine. The Biojector 2000® has FDA clearance for delivering vaccine injections into muscles in children and adults. The TIV shot will be given using a needle and syringe in the usual way.

After each vaccination, your child will need to remain in the clinic for between15 to 60 minutes after the shot for observation.

Phone Calls and Diary cards

One to two days after each vaccination, the study staff will contact you to check on how your child is doing. You will be asked to help your child complete a diary card for 7 days at home. You may use a paper or a secure electronic diary card over the internet. If you choose using internet, the clinic staff will train you and give you a username and password. Completing a diary card will require recording your child's temperature and symptoms. You will need to look at the injection site on his/her arm each day. The study staff will provide you with contact information to report any unexpected side effects. If your child has any usual symptoms, it may be necessary to come to the clinic for extra visits.

Blood Sample Collections

Blood will be drawn at scheduled visits (see chart below) to check on your child's health and to study immune responses. You will be told promptly if any of the test results show a health problem. The blood drawn for research tests of "immune response" are not used to check on your child's health and the results will not be given to you during the study.

The amount of blood drawn will vary from about 1 tablespoon (15 mL) to about 3 tablespoons (45 mL), depending on the visit. Your child may also be asked to have tests between regular visits if needed to evaluate a change in his/her health.

Blood will be drawn from veins in the arms. The study staff will discuss the blood draw plan with you and your child before starting.

The total volume of blood collected for all reasons, such as testing done by your doctor, must be considered before we collect research samples so as not to go over allowed limits. You must notify the study staff if your child has blood drawn for another reason so that your study sample collections can be scheduled properly.

The study schedule is shown in the following table:

**Week of Study**	Screen for study	Start	Week 1 /Day 1 or 2	Week 1 /Day 7	Week 4	Week 18	Week 19	Week 22	Week 42
Informed Consent / Assent	X								
Health review and check-up	X	X		X	X	X		X	X
Vaccinations		DNA or TIV				TIV			
Begin 7-Day Diary Card		X				X			
Telephone contact			X				X		
Pregnancy test (if applicable)	X	X			X	X			
Blood drawn; including samples stored for research	X				X	X		X	X

<u>Blood Sample Storage and Future Use</u>

If you agree to allow your child to take part in this study, your child's blood samples will be stored and used for research related to flu, vaccines, the immune system and related medical research. We may also store some of the samples to be used in future research. The blood may be used to learn more about vaccines, other infectious diseases and the immune system in general.

The results from the research done with your child's stored samples will not be given to you or your private doctor, and will not be in your child's medical record. Your child may not be in this study if you are not willing to have his/her blood samples stored for future research testing.

The stored samples will be labeled by a code (such as a number). Only the study team can link the samples back to your child. Any identifying information about you and your child will be kept confidential to the extent permitted by law.

In the future, other investigators at NIH or outside of NIH may wish to study your child's stored samples. All uses must be in compliance with the approved uses for the samples. When the study team shares your stored samples, they will be marked with a code that will not identify your child. Some information about your child, such as gender, age, health history, or ethnicity may be shared with other investigators.

Your stored samples will be used only for research and will not be sold. The research done with the samples may be used to develop new products in the future but you will not receive payment for such products.

3. Costs to you/your child if you take part in this study:

There is no cost to you or your child for taking part in this study.

4. Side effects and risks that your child can expect if you take part in this study:

While in the study, your child may be at risk for some side effects. You should discuss these with the study doctor and/or your regular doctor. There also may be other side effects that we cannot predict. Most side effects go away shortly after the flu shot is given, but rarely, side effects of vaccines can be serious or long lasting.

Vaccine Injections
People who receive vaccines by injection can experience pain, soreness, stinging, redness, swelling, bruising or itchiness at the injection site. There is a very small chance of infection. General symptoms from vaccine may include fever, chills, rash, aches and pains, nausea, headache, dizziness, and fatigue. Vaccine reactions are usually seen within the first 24 hours after receiving the vaccine. Reactions rarely start later or last more than 3 days. Over-the-counter medicine, such as acetaminophen (Tylenol), will generally help relieve symptoms. An allergic reaction is also a risk for any vaccine. These usually happen soon after the injection. This is why we will ask you to wait in the clinic with your child for observation after the injection.

DNA Vaccine
DNA vaccines made by NIH have been tested against a variety of infections. Between 1 and 4 injections of a DNA vaccine have been given to about 2300 adults over the past 10 years. In clinical studies, some people had a temporary drop in white blood cell count, sore arm, skin rash, or hives. Some people get a small scab for a few days where the shot is given.

TIV Vaccine
TIV is the traditional licensed influenza vaccine. Some people have fever, muscle aches, general body aches, headache, tiredness, and nausea. A severe allergic reaction can occur especially in people that are allergic to eggs. In 1976, a small number of people who got an inactivated swine flu vaccine developed a severe nerve weakness called Guillain-Barré syndrome. Guillain-Barré has not been linked to any other flu vaccines.

Blood draws
Having blood taken can cause discomfort. The discomfort is temporary but may cause fainting.

Having blood taken can cause bruising. Bruising can be prevented or reduced by putting pressure on the blood draw site for a few minutes after the blood is taken. It is possible to get an infection from having blood taken or receiving a shot. This is not very likely. To reduce the risk of infection, the area where the blood will be drawn will be cleaned, and sterile equipment will be used.

Blood Donation
Your child may not donate blood at a blood bank while being in this study, or for one year after the date of the last injection of the DNA vaccine.

Risk to pregnancy

We do not know the effects that the experimental DNA vaccine may have on an unborn child. If your daughter is of reproductive age and takes part in this study, she must use an approved birth control method starting at least 3 weeks prior to joining the study and until 4 weeks after she gets the last study vaccination. The birth control methods that work well enough to be safe while you are on this study are abstinence, condoms, effective intrauterine devices (IUDs), hormonal shots or hormonal implants, oral contraceptives, contraceptive patches, NuvaRing or other licensed hormonal products that are given by prescription.

If your daughter cannot abstain or use an approved birth control method, she cannot take part in this study. If you child becomes pregnant during the restricted time of the study, you must report this to the research doctor right away.

The licensed TIV flu vaccine is approved and recommended for use during pregnancy.

5. Risks that are not known:

The DNA vaccine used in this study is experimental. There is a chance that there are risks that are unknown. Study staff will update you and your child in a timely way on any new information that may affect your child's health, welfare, or decision to stay in this study. Please let the study doctor know of any side effects that your child may have.

6. Payment in case your child is injured because of this research study:

The study site will provide immediate medical care for any injury resulting from your child's participation in this research study. There are no plans for [study site] to pay for the costs of any additional care or to give you or your child money for the injury.

No long-term medical care or financial compensation for research-related injuries will be provided by the National Institutes of Health (NIH) or the Federal Government. However, you have the right to pursue legal remedy if you believe that your injury justifies such action.

7. **Good effects that might result from this study:**

- The benefits to science and humankind that might result from this study.
  The information gathered during this study may help researchers make better flu vaccines.

- The benefits your child might get from being in this study.
  Your child may not receive any benefit from taking part in this study. Receiving the TIV vaccine may prevent your child from getting the flu. Receiving the DNA priming vaccine may improve the overall response to the TIV vaccine.

8. Other treatments your child could get if you decide for them to not be in this study:

You may choose not to allow your child to be in this study, without changing your child's healthcare, services or other rights. Your child can receive the TIV flu vaccine from his or her primary care doctor or other places it is given every year.

9. Payments for your time spent taking part in this study or expenses:

Your child will receive compensation for each visit completed:
Screening Visit = $xx
Enrollment Visit = $xx
Follow-up Visits = $xx each
Planned phone call after vaccination = $xx each (for 2)

For a total of up to **$xxx** for completing all planned study visits.

10. Reasons why the study doctor may take your child out of this study:

The study doctor may decide to stop your child from taking part in this study at any time. Your child could be removed from the study for reasons related to the child (for example, if you move to another city or if your child has a serious reaction to the flu shot). The reason that your child may be withdrawn will be explained to you. Also, the sponsor, the study doctor or Institutional Review Board (IRB) may stop the study at any time.

11. What will happen if you decide to stop your child from being in this study?

If your child has received the first vaccine, but for any reason is no longer taking part in the study, we will ask you to allow your child to continue with follow-up visits. You will be compensated for the study visits that your child completes.

12. Who to call for any questions or in case your child is injured:

If you or your child has any questions about this research study or if you or your child feels they have been hurt by being a part of this study, please feel free to contact [Principal Investigator] and/or the study staff at [phone number].

For additional information about giving consent or your rights as a person in this study, to discuss problems, concerns, and questions, or to offer input, please feel free to call the [insert site Institutional Review Board Office and/or Patient Advocate contact information].

13. Clinical Trials Registry:

A description of this clinical trial will be available on www.clinicaltrials.gov, as required by U.S. Law. This web site will not include information that can identify you. At most, the web site will include a summary of the results. You can search this web site at any time.

14. Confidentiality:

All efforts, within reason, will be made to keep your child’s personal information and their research record confidential. Total confidentiality cannot be guaranteed. A risk of participation is that the confidentiality of your child’s personal health information may be lost. Records and documents about the conduct of this study are kept in locked files. Study records are labeled

with a code identifying your child only by study number and will not be labeled with your child's name. Personal data such as your child's name and contact information are stored by a secure method.

This study will be monitored by a group of physicians and scientists associated with the National Institutes of Health. This group will review the information from the study and will pay close attention to harmful reactions.

Access to study documents will be limited to [study site], the FDA, the IRB, the National Institutes of Health (NIH), and its associates. The FDA, the NIH, and its authorized contractors, and the IRB may have access to your name if they ask to inspect the Informed Consent; no other identifiable information will be shared.

The information from the research study may be published; however, your child will not be identified. The publication will not contain information about you that would enable someone to identify your child unless you give permission.

15. Authorization to Use/Disclose Protected Health Information:

All efforts, within reason, will be made to keep your child's protected health information (PHI) private. PHI is your child's health information that is, or has been gathered or kept by, [study site] as a result of their healthcare. This includes data gathered for research studies that can be traced back to your child. Using or sharing ("disclosure") such data must follow federal privacy rules.

By signing the consent for this study, you are agreeing (giving "authorization") to the use and likely sharing of your child's PHI. If you decide to allow your child to be in this research study, you are also agreeing to let the study team use and share your child's PHI as described below.

The sponsor may give your child's health data, without your child's name, to others or use it for other research projects.

Your child's research record will be kept at [site] after the study is finished for at least 3 years unless NIH and [site] approves its destruction or transfer. Any research data that has been put into your child's medical record will be kept for an unknown length of time.

Your consent to use or share your child's PHI does not expire. If you or your child has a change of mind, we ask that you contact [PI; contact information] to state in writing that you and/or your child have withdrawn consent. At that time, we will stop getting any more data about your child. But, the health data we stored before you withdrew consent may still be used for reporting and research quality.

<u>If you decide not to allow your child to take part in this research study, it will not affect your child's treatment, payment or enrollment in any health plans or affect your child's ability to get benefits. You will get a copy of this form after it is signed**.**</u>

STATEMENT BY PERSON AGREEING TO BE IN THIS STUDY

I have read this consent form and the research study has been explained to me verbally. All my questions have been answered, and I freely and voluntarily choose to allow my child to take part in this study.

Minor Child's Name (print)	Date
Parent or Legal Guardian Name (print)	
Signature	
Investigator name (print)	Date
Signature	
Witness name (print)	Date
Signature	

Child's Verbal Assent (if applicable):

The information in the above consent was described to the minor child who is legally under my care and he/she agrees to participate on the study.

Minor Child's Name (print)	Date
Parent or Legal Guardian Name (print)	
Signature	
Investigator name (print)	Date
Signature	
Witness name (print)	Date
Signature	

Minor Assent Template for VRC 702 Study

Study Title: VRC 702: An Open-Label, Dose-Escalation, Phase I Study of the Safety, Tolerability and Immunogenicity of the Prime-Boost Regimen of the Investigational 2012/13 Seasonal Influenza DNA Vaccine, VRC-FLUDNA063-00-VP, Followed by the 2012/2013 Seasonal Influenza Trivalent Inactivated Vaccine (TIV) Compared to TIV Prime-TIV Boost in Children and Adolescents Ages 6-17 Years

What is Research?

We are asking you to be in a research study. Research is a way to test new ideas. Research helps us learn new things. Being in research is your choice. You can say Yes or No. Whatever you decide is OK. We will still take good care of you.

Why is this Research Study Being Done?

The doctors want to understand why kids, become sick with the "flu" (influenza). The doctors want to learn how to protect kids better with vaccines.

What Will You Do in this Study?

In this study, a new flu vaccine is going to be tested along with the regular flu vaccine. If you say yes to be in the study, you will have to come in about 7 times. You will get two study injections (shots) in your upper arm. You will also have blood collected 5 times for the study.

The research flu vaccine will be injected with a special device called a "Biojector". The regular flu vaccine will be injected using needle and syringe. If you want, your parent(s) or guardian may be with you in the room each time you get a shot or the blood is collected.

If you are a girl who is old enough to get pregnant, you must not have sex or you must use birth control to be in this study.

We will ask you to tell us how you feel every day for 7 days after each shot. You can use paper or a safe Internet site for reporting.

Your information and blood may help us to find better ways to stop people from getting the flu.

What Might Happen During this Study?

Blood will be taken from your arm with a needle. This may hurt for a short while or may bleed a few drops, but that stops quickly.

Your arm may hurt where you get the shots and you may have a small scab.

What Else Should You Know About the Research?

You can change your mind later about being in the research study. You can stop being in the study at any time. If you want to stop, tell one of the doctors or nurses.

It is also okay to ask more questions after you decide to be in the research. You can ask questions at any time.

I have had this study explained to me in a way that I understand. I have had the chance to ask questions. I agree to take part in this research study.

Minor name (print)	Date
Signature	
Parent/Guardian name (print)	Date
Signature	
Investigator name (print)	Date
Signature	
Witness name (print)	Date
Signature	

Appendix II: Contact Information

VRC Scientific/Medical Researchers:
Protocol Chair
Julie Ledgerwood, D.O.
Phone 301-594-8502
Vaccine Research Center, NIAID, NIH
Bethesda, MD 20892

Protocol Co-Chair
Uzma Sarwar, M.D.
Phone 301-402-9043
Vaccine Research Center, NIAID, NIH
Bethesda, MD 20892

IND Sponsor Medical Officer
Barney Graham, M.D., Ph.D.
Phone 301-594-8468
Alternate: Joseph Casazza, M.D., Ph.D.
Phone 301-594-8627
Vaccine Research Center, NIAID, NIH
Bethesda, MD 20892

Contracting Officers' Technical Representative (COTR):
Brenda Larkin, RN, BSN, CCRC.
Phone 301-594-8542
Vaccine Research Center, NIAID, NIH
Bethesda, MD 20892

Protocol Statistician:
Abbie Bellamy, Ph.D. 301-251-1161
The EMMES Corporation

VRC Protocol Operations Manager:
Mary E. Enama, M.A., PA-C, 301-594-8501
Vaccine Research Center, NIAID, NIH
Bethesda, MD 20892

VRC Protocol Specialist:
Galina Yamshchikov, M.S., 301-594-1064
Vaccine Research Center, NIAID, NIH
Bethesda, MD 20892

CRO Authorized to Conduct the Study on Behalf of the VRC/NIAID/NIH:
The EMMES Corporation
401 N. Washington Street, Suite 700
Rockville, MD 20850

Scientific and Laboratory Collaborators:
Vaccine Research Center, NIAID, NIH
40 Convent Drive
Bethesda, MD 20892
Gary Nabel, M.D., Ph.D.
Robert Bailer, Ph.D., 301-594-8481
Richard Koup, M.D., 301-594-8585
John Mascola, M.D., 301-594-8490
Mario Roederer, Ph.D., 301-594-8491
Daniel Douek, M.D., Ph.D., 301-594-8484
Robert Seder, M.D., 301-594-8483

Research Immunology Central Laboratory:
NVITAL (NIAID Vaccine Immune T-Cell and Antibody Laboratory)
9 West Watkins Mill Road, Suite 150
Gaithersburg, MD 20878

VRC Vaccine Production Program
Richard Schwartz, Ph.D. 301-594-8485
Vaccine Research Center, NIAID, NIH
Bethesda, MD 20892

Other Immunology Testing Labs:
Bioqual, Inc., 9600 Medical Center Dr.,
Rockville, MD 20850

UNI-CPSC Operations Management Center
Thad Zajdowicz, M.D., MPH,
Project Director and Medical Monitor
Alternate Med. Monitor: Robert Lindblad, M.D.
Phyllis Renehan, Project Manager
The EMMES Corporation, 301-251-1161
401 N. Washington Street, Suite 700
Rockville, MD 20850

IND Sponsor Regulatory Affairs
Michelle Conan-Cibotti, Ph.D. 301-451-2740
Florence A. Kaltovich, MS, MHS 301-402-2402
Vaccine Research Center, NIAID, NIH
Bethesda, MD 20892

IND Sponsor Authorized Representative
Jamie Winestone 301-251-1161 ext. 230
UNI-CPSC Regulatory Director

Serious Adverse Event Reporting:
Email: uniflu@emmes.com
Fax: 301-576-3558

VRC 702 Study Sites	Site Investigators of Record
Cincinnati Children's Hospital Medical Center;<br>Cincinnati, OH	David Bernstein, MD
Vanderbilt University;<br>Nashville, TN	Buddy Creech, MD
Saint Louis University;<br>St. Louis, MO	Edwin Anderson, MD
Emory University, Children 's Medical Center;<br>Atlanta, GA	Harry Keyserling, MD
Dartmouth Hitchcock Medical Center;<br>Lebanon, NH	Peter Wright, MD

Appendix III: Schedule of Evaluations

		Screen	VRC 702 Schedule of Evaluations							
**Visit**		01	02	02A	03	04	05	05A	06	07
**Week of Study**		-10 to 0	W 0	W 1	W 1	W 4	W 18	W 19	W 22	W 42
[1]**Day of Study**		-70 to 0	D 0	D2	D7	D 28	D 126	D 133	D 154	D 294
**Clinical Evaluations**	**Tube**									
[1]Informed Consent		X	X							
Physical exam for eligibility at screen; BP, pulse, temp, wt other visits; targeted exam.		X	X		X	X	X		X	X
Medical history targeted to eligibility at screen; interim history for AEs other visits		X	X		X	X	X		X	X
[2] Study Vaccinations			X*				TIV			
Begin 7-Day Diary Card			X				X			
Telephone contact; clinic visit if indicated				X				X		
If applicable, pregnancy prevention counseling			X		X	X				
Hemoglobin	EDTA	2				2				
[3] Pregnancy test: urine (or serum)		X	X			X	X			
Creatinine and ALT	SST	4				4				
[1]**Research Immunology**										
Antibody assays and serum storage	SST	8				8	8		8	8
PBMC and plasma for storage**	EDTA	[20]				[10]			[20]	
**Daily Volume (mL)**		**34**	**-**	**-**	**-**	**24**	**8**	**-**	**28**	**8**
**Max. Cumulative Volume (mL)**		**34**	**-**	**-**	**-**	**58**	**64**	**-**	**92**	**100**

[1] Screening informed consent may be signed more than 10 weeks prior to study enrollment; screening evaluations may be repeated if needed. Day 0 evaluations prior to first study injection are the baseline for assessing adverse events, except screening or medical history evaluations may be used for assessments not done on Day 0.
When possible, attempt to complete the screening blood draws within the 2 weeks prior to Day 0. The screening draw may be done on Day 0, if needed.
For Groups 1 and 2, Day 0=day of randomization and DNA vaccine injection, which is administered the same day.
For Group 3, Day 0=day of TIV prime injection, which may be scheduled after the day of randomization if 2012/2013 TIV is not yet available.
**PBMC and plasma collection volumes shown in square brackets [ ] are performed only at sites that have passed proficiency testing. All sites will collect serum samples for the antibody assays. The volumes shown are the estimated maximum and will be less at sites not collecting PBMC and plasma for storage.
[2] Complete post vaccination evaluations (BP, pulse and injection site assessment) at 30-60 min after HA DNA and at 15-60 min after TIV injection.
[3] Negative pregnancy test results must be confirmed for adolescent females of reproductive potential prior to administering the DNA vaccine injection. Licensed TIV is not contraindicated for pregnant women, but pregnancy testing will be conducted and results reported prior to administering TIV as part of this research protocol. If pregnant, research blood draws will be discontinued.
Visit windows: Schedule Visits 02A through Visit 05 with respect to Day 0. The following visit windows apply: 02A (+2 days); Visit 03 (+3 days); Visit 04 (±7 days); Visit 05 (±14 days). Schedule Visit 05A at 7 to 10 days after Visit 05 and Visit 06 and 07 at 4 and 24 weeks, respectively, after Visit 05. Visits 06 and 07 each have a ±7 day window. Visit 05 may be earlier than targeted if community exposure to influenza is likely; consult with IND Sponsor.
Blood drawing limitations: No more than 3 mL/kg may be drawn over any 8-week period.

Appendix IV: Assessment of Relationship to Vaccine and Adverse Event Severity Grading

<u>Assessment of Causality Relationship of an Adverse Event (AE) to Study Vaccine</u>:

The relationship between an AE and the vaccine will be assessed by the investigator on the basis of his or her clinical judgment and the definitions below.

- **Definitely Related**. The AE and administration of study agent are related in time, and a direct association can be demonstrated.
- **Probably Related**. The AE and administration of study agent are reasonably related in time, and the AE is more likely explained by study agent than other causes.
- **Possibly Related**. The AE and administration of study agent are reasonably related in time, but the AE can be explained equally well by causes other than study agent.
- **Not Related**. There is not a reasonable possibility that the AE is related to the study agent.

For purposes of preparing data reports in which AE attributions are limited to "**Related**" or "**Not Related**", in this protocol, the "Definitely, Probably and Possibly" attributions will be mapped to the "Related" category. The definitions that apply when these two categories alone are used are as follows:

- **Related** – There is a reasonable possibility that the AE may be related to the study agent.
- **Not Related** – There is not a reasonable possibility that the AE is related to the study agent.

Grading the Severity of Adverse Events:

The FDA Guidance for Industry (September 2007): "Toxicity Grading Scale for Healthy Adult and Adolescent Volunteers Enrolled in Preventive Vaccine Clinical Trials" is the basis for the severity grading of adverse events in this protocol. Several modifications were made to the table in order to adapt its use for children and adolescents as follows:

- "Emergency room visit" is not automatically considered a life-threatening event; these words have been removed from "grade 4' definition where they appear in the guidance document.
- Any laboratory value shown as a "graded" value in the table that is within the institutional normal range will not be severity graded or recorded as an adverse event.
- Severity grading for hemoglobin is based on a Division of AIDS, NIAID table and only absolute hemoglobin will be used to define grade 1; grades 2 and higher may be based on either absolute count or decrease from baseline. An increase in hemoglobin will not be recorded as an AE unless it is above the site ULN and will be graded based on the "not otherwise specified" lab criteria.
- Severity grading definitions for Erythema/Redness and Induration/Swelling are more conservative (sizes about half of the standard table) and for Grade 4, the table includes added text "requiring medical attention".
- Several of the infrequently evaluated lab parameters are removed from the table and a general definition based on clinical judgment for the not otherwise specified laboratory values is included.

When not otherwise specified in the table, the following guidance will be used to assign a severity grade:

Grade 1 (Mild): No effect on activities of daily living
Grade 2 (Moderate): Some interference with activity not requiring medical intervention
Grade 3 (Severe): Prevents daily activity and requires medical intervention
Grade 4 (Life-threatening): Hospitalization; immediate medical intervention or therapy required to prevent death.
Grade 5 (Death): Death is assigned a Grade 5 severity.
Only one adverse event assessed as the primary cause of death should be assigned "grade 5" severity.

Modified from the Toxicity Grading Scale for Healthy Adult and Adolescent Volunteers Enrolled in Preventive Vaccine Clinical Trials (FDA Guidance - September 2007)

A. Tables for Clinical Abnormalities

**Local Reaction to Injectable Product**	**Mild (Grade 1)**	**Moderate (Grade 2)**	**Severe (Grade 3)**	**Potentially Life Threatening (Grade 4)**
Pain	Does not interfere with activity	Repeated use of non-narcotic pain reliever > 24 hours or interferes with activity	Any use of narcotic pain reliever or prevents daily activity	Hospitalization
Tenderness	Mild discomfort to touch	Discomfort with movement	Significant discomfort at rest	Hospitalization
Pediatric ≤ 17 years<br>[1]Erythema/Redness	1 – 2.5 cm	2.6 – 5 cm	> 5 cm	Necrosis or exfoliative dermatitis requiring medical attention
Pediatric ≤ 17 years<br>[2] Induration/Swelling	1 – 2.5 cm and does not interfere with activity	2.6 – 5 cm or interferes with activity	> 5 cm	Necrosis requiring medical attention
**[3] Vital Signs**	**Mild (Grade 1)**	**Moderate (Grade 2)**	**Severe (Grade 3)**	**Potentially Life Threatening (Grade 4)**
[4] Fever (°C)<br>(°F)	38.0 – 38.4<br>100.4 – 101.1	38.5 – 38.9<br>101.2 – 102.0	39.0 – 40<br>102.1 – 104	> 40<br>> 104
[5]Hypertension (HTN)<br>Pediatric ≤ 17 years<br>(with repeat testing at same visit)	Prehypertension	Stage 1 HTN	Stage 2 HTN	Life-threatening consequences; malignant hypertension OR Hospitalization indicated

1. In addition to grading the measured local reaction at the greatest single diameter, the measurement should be recorded as a continuous variable.
2. Induration/Swelling should be evaluated and graded using the functional scale as well as the actual measurement.
3. Subject should be at rest for all vital sign measurements; Use clinical judgment when characterizing bradycardia among some healthy subject populations, for example, conditioned athletes.
4. Oral temperature; no recent hot or cold beverages or smoking.
5. Definitions as in the NHLBI "Pocket Guide to Blood Pressure Measurement in Children". http://www.nhlbi.nih.gov/health/public/heart/hbp/bp_child_pocket/bp_child_pocket.pdf

**Systemic (General)**	**Mild (Grade 1)**	**Moderate (Grade 2)**	**Severe (Grade 3)**	**Potentially Life Threatening (Grade 4)**
Nausea/vomiting	No interference with activity or 1 – 2 episodes/24 hours	Some interference with activity or > 2 episodes/24 hours	Prevents daily activity, requires outpatient IV hydration	Hospitalization for hypotensive shock
Diarrhea	2 – 3 loose stools or < 400 gms/24 hours	4 – 5 stools or 400 – 800 gms/24 hours	6 or more watery stools or > 800gms/24 hours or requires outpatient IV hydration	Hospitalization
Headache	No interference with activity	Repeated use of non-narcotic pain reliever > 24 hours or some interference with activity	Significant; any use of narcotic pain reliever or prevents daily activity	Hospitalization
Fatigue	No interference with activity	Some interference with activity	Significant; prevents daily activity	Hospitalization
Myalgia	No interference with activity	Some interference with activity	Significant; prevents daily activity	Hospitalization

**Systemic Illness**	**Mild (Grade 1)**	**Moderate (Grade 2)**	**Severe (Grade 3)**	**Potentially Life Threatening (Grade 4)**
Illness or clinical adverse event (as defined according to applicable regulations)	No interference with activity	Some interference with activity not requiring medical intervention	Prevents daily activity and requires medical intervention	Hospitalization

B. Tables for Laboratory Abnormalities

**Serum ***	**Mild (Grade 1)**	**Moderate (Grade 2)**	**Severe (Grade 3)**	**Potentially Life Threatening (Grade 4)**
Sodium – Hyponatremia mEq/L	132 – 134	130 – 131	125 – 129	< 125
Sodium – Hypernatremia mEq/L	144 – 145	146 – 147	148 – 150	> 150
Potassium – Hyperkalemia mEq/L	5.1 – 5.2	5.3 – 5.4	5.5 – 5.6	> 5.6
Potassium – Hypokalemia mEq/L	3.5 – 3.6	3.3 – 3.4	3.1 – 3.2	< 3.1
Glucose – Hypoglycemia mg/dL	65 – 69	55 – 64	45 – 54	< 45
Glucose – Hyperglycemia<br>Fasting – mg/dL<br>Random – mg/dL	 100 – 110<br>110 – 125	 111 – 125<br>126 – 200	 >125<br>>200	Insulin requirements or hyperosmolar coma
Liver Function Tests – ALT, AST increase by factor	1.1 – 2.5 x ULN	2.6 – 5.0 x ULN	5.1 – 10 x ULN	> 10 x ULN
**Hematology ***	**Mild (Grade 1)**	**Moderate (Grade 2)**	**Severe (Grade 3)**	**Potentially Life Threatening (Grade 4)**
Hemoglobin - gm/dL	10.0 – 10.9 g/dL	9.0 – 9.9 g/dL	7.0 – 8.9 g/dL	< 7.0 g/dL
Hemoglobin - decrease from baseline value - gm/dL	not applicable	1.6 – 2.0	2.1 – 5.0	> 5.0
WBC Increase - cell/mm^3	1.25xULN – 15,000	15,001 – 20,000	20,001 – 25, 000	> 25,000
WBC Decrease - cell/mm^3	2,500 – <LLN	1,500 – 2,499	1,000 – 1,499	< 1,000
Lymphocytes Decrease - cell/mm^3	750 – <LLN	500 – 749	250 – 499	< 250
Neutrophils Decrease - cell/mm^3	1,500 – <LLN	1,000 – 1,499	500 – 999	< 500
Platelets Decreased - cell/mm^3	125,000 – <LLN	100,000 – 124,999	25,000 – 99,999	< 25,000
Laboratory values *not otherwise specified* in this table	Abnormal, but requiring no immediate intervention	Sufficiently abnormal to require evaluation, but not of sufficient severity to warrant immediate changes in the study	Sufficiently severe to require evaluation and treatment, and consideration of the study pause rules	Life-threatening; Requires immediate evaluation, treatment, and hospitalization; and consideration of the study pause rules

* The laboratory values provided in the tables serve as guidelines and are dependent upon institutional normal parameters. Institutional normal reference ranges should be provided to demonstrate that they are appropriate.

“ULN” is the upper limit of the normal range; “LLN” is the lower limit of normal.
